# Network Pharmacology and Molecular Simulation Reveal Potential Targets and Pathways of Vine Tea in Feline Intestinal Inflammation

**DOI:** 10.3390/vetsci13090895

**Published:** 2026-08-31

**Authors:** Bochi Zhang, Rui Zhao, Liying Du, Kai Guo, Kai Zhang, Chunlei Yang, Xianyi Song

**Affiliations:** College of Animal Science, Shanxi Agricultural University, Taiyuan 030032, China

**Keywords:** vine tea, cats, intestinal inflammation, network pharmacology, molecular docking and molecular dynamics simulation

## Abstract

Feline intestinal inflammation is a common gastrointestinal disorder in cats and is associated with immune dysregulation, epithelial barrier injury, microbial imbalance, and persistent inflammatory signaling. This entirely in silico study investigated predicted molecular associations between vine tea (*Ampelopsis grossedentata*) constituents and feline intestinal inflammation using network pharmacology, molecular docking, and molecular dynamics simulation. No feline cells, tissues, animals, or clinical samples were used. Candidate compounds were screened and evaluated with SwissADME as a computational prioritization step, and potential targets were predicted using multiple databases. Human-centric database-derived genes associated with the broad intestinal-inflammation phenotype were subjected to feline ortholog mapping/verification and intersected with predicted vine tea targets. Protein–protein interaction, Gene Ontology, and Kyoto Encyclopedia of Genes and Genomes enrichment analyses were performed, followed by construction of a compound-target-pathway-disease network. Ten representative compounds were cross-docked against six core targets, producing 60 docking combinations, and the lowest-energy complex was subjected to a 100 ns molecular dynamics simulation. Seventeen candidate compounds, 966 predicted vine tea targets, and 102 candidate shared targets were retained in the archived workflow. IL6, TNF, IL1B, STAT3, CASP3, and TLR4 were prioritized as core targets. Enrichment analysis linked the shared-target set to inflammatory responses, innate immunity, cytokine activity, Th17 cell differentiation, and the IL-17, Toll-like receptor, NOD-like receptor, NF-kappa B, TNF, JAK-STAT, and PI3K-Akt signaling pathways. Molecular docking predicted favorable interactions between several compounds and the core targets, particularly IL6, and the IL6-cianidanol complex showed the lowest docking energy and relative stability during simulation. Overall, the analyses prioritize candidate compounds, targets, and pathways for subsequent feline-specific validation; they do not demonstrate therapeutic efficacy, target regulation, or biologically relevant exposure in cats.

## Simple Summary

Feline intestinal inflammation is a common and difficult-to-manage disorder that can cause vomiting, diarrhea, weight loss, and repeated relapse. Vine tea, produced from *Ampelopsis grossedentata*, contains multiple flavonoids and other natural compounds reported to have anti-inflammatory activity in non-feline experimental systems. In this entirely in silico study, network pharmacology, molecular docking, and molecular dynamics simulation were combined to computationally explore molecular associations between vine tea constituents and feline intestinal inflammation. No feline cells, tissues, animals, or clinical samples were used. Seventeen candidate compounds and 102 shared candidate targets were identified. IL6, TNF, IL1B, STAT3, CASP3, and TLR4 were highlighted as central targets, while the main enriched pathways involved inflammatory signaling, innate immunity, Th17-cell responses, and intestinal epithelial survival. Molecular docking predicted favorable interactions between several candidate compounds and the core targets, particularly IL6, and the IL6-cianidanol complex showed relative stability during a 100 ns molecular dynamics simulation. Overall, these computational results define a multi-component, multi-target, and multi-pathway hypothesis for future feline-specific testing rather than evidence of therapeutic efficacy. Experimental validation and feline pharmacokinetic and safety studies are required before vine tea can be considered for veterinary application.

## 1. Introduction

Feline intestinal inflammation is a common group of digestive diseases in cats. It includes food-responsive enteropathy, idiopathic inflammatory bowel disease, and low-grade small-cell lymphoma. Affected cats often show repeated vomiting, diarrhea, and weight loss [1]. Feline inflammatory bowel disease mainly features persistent or repeated immune-mediated inflammation in the gastrointestinal tract. The lesions can involve the stomach, small intestine, or colon. They can also occur together with inflammation of the pancreas and hepatobiliary system [2]. The clinical signs are not specific. They also overlap with food intolerance, parasite infection, and intestinal lymphoma. A clear diagnosis usually requires stepwise exclusion and intestinal mucosal biopsy [3]. Current treatment mainly uses prescription diets, glucocorticoids, and other immunosuppressive measures. Some cases respond poorly or relapse easily. Long-term drug use can also cause adverse effects [4,5]. Changes in mucosa-associated bacteria are closely related to tissue damage, inflammatory cytokine expression, and disease activity. These findings indicate that dysbiosis, immune disorder, and barrier injury may form a self-reinforcing disease cycle [6].

Recent studies on hydrolyzed protein diets, fecal microbiota, lipids, and bile acid metabolism show that FCE varies widely among cats. These studies also show that FCE is controlled at several biological levels. A single-target treatment cannot fully explain its onset or outcome [7,8,9]. Vine tea is made from the young stems and leaves of *Ampelopsis grossedentata*, a plant in the Vitaceae family. It is used as both food and medicine. It is rich in flavonoids, polyphenols, and organic acids [10]. Dihydromyricetin is one of the main flavonoids in vine tea. It has antioxidant, anti-inflammatory, and metabolic regulatory activities. Its stability, bioavailability, and action network still need deeper study [11]. Previous studies showed that vine tea water extract and dihydromyricetin can reduce oxidative stress and intestinal mucosal inflammation. They can also improve dysbiosis and bile acid metabolism. They can further support intestinal barrier repair [12,13,14]. Dihydromyricetin can promote IL-22 expression and restore tight junction proteins through AMPK/SIRT3/STAT3 signaling. This mechanism supports its role in mucosal immune balance [15].

Vine tea contains many chemical components. Reported anti-inflammatory activities of vine tea in non-feline experimental systems have been associated with oxidative stress, apoptosis, and several signaling axes, such as MAPK, Akt, NF-kappa B, and PPAR [16]. The traditional one-compound, one-target model cannot fully explain this mechanism. In rodent experimental colitis models, vine tea flavonoids such as quercetin, kaempferol, and taxifolin have been reported to reduce IL-1beta, IL-6, and TNF-alpha, inhibit TLR4/NF-kappa B signaling, alter microbiota composition, and support tight-junction integrity [17,18,19]. Network pharmacology can combine drug components, targets, and disease networks. It is suitable for studying the multi-component and multi-target effects of natural products [20]. This approach also fits the holistic regulation concept of traditional Chinese medicine. It can screen core targets, build protein interaction networks, and predict key biological processes and pathways [21,22,23,24]. Therefore, this entirely in silico study combined network pharmacology, molecular docking, and molecular dynamics simulation to prioritize candidate vine tea constituents, shared targets, and pathways associated with feline intestinal inflammation and to evaluate predicted docking modes and simulation stability of representative compound-target complexes. The study was designed to generate testable hypotheses for subsequent feline-specific experimental validation.

## 2. Materials and Methods

### 2.1. Study Design and Reporting Scope

This study was designed as an entirely in silico investigation integrating database mining, network pharmacology, bioinformatics enrichment, AlphaFold-based protein structure prediction, molecular docking, and molecular dynamics simulation. No live cats or other animals were enrolled, treated, experimentally challenged, sampled, or euthanized, and no newly collected animal tissues or biological specimens were used. Accordingly, all compound-target associations, core targets, enriched pathways, docking poses, binding energies, and dynamic stability parameters reported in this study are computational predictions or simulation outputs. They are intended to generate mechanistic hypotheses and prioritize candidate compounds and targets for subsequent validation. These results must not be interpreted as direct evidence that vine tea produces a therapeutic effect in cats, regulates the expression or activity of the predicted targets in vivo, or exhibits direct anti-inflammatory, antimicrobial, or anti-mycoplasma activity. Because no live animals or newly collected animal samples were used, institutional animal ethics approval was not applicable. Experimental confirmation in feline cells, organoids, ex vivo tissues, animal models, or clinical samples is required before biological or therapeutic conclusions can be established.

### 2.2. Screening of Candidate Compounds in Vine Tea and Prediction of Potential Targets

This study used vine tea (*Ampelopsis grossedentata*) as the research object. Candidate chemical components were collected from BATMAN-TCM, HERB, and related reports. Compound names, PubChem CIDs, molecular formulas, molecular weights, Canonical SMILES, InChI records, and three-dimensional structures were checked in PubChem. The compound structures were then imported into SwissADME for drug-likeness evaluation. The reconstructed retention rule that reproduced the archived 17-compound set was predicted gastrointestinal absorption = High AND (at least three of the five drug-likeness rules—Lipinski, Ghose, Veber, Egan, and Muegge—showing zero violations OR the maximum number of violations in any one of the five rules being ≤1). These human-oriented SwissADME rules were used only as pragmatic in silico prioritization filters and were not treated as evidence of oral bioavailability or therapeutic exposure in cats. Duplicate compounds, compounds with incomplete structural information, and compounds that could not be used for later target prediction were removed. The remaining compounds were defined as candidate compounds of vine tea. Because no physical vine tea batch, extract, or formulation was chemically analyzed in this study, database and literature provenance was interpreted only as evidence that a constituent or its parent compound had been reported for A. grossedentata, not as batch-specific confirmation or quantitative abundance. The complete 55-record screening audit and the provenance audit of the 17 retained compounds are provided in Supplementary Data S1 (Table S1A,B). Each active compound was submitted to TargetNet, SEA, BATMAN-TCM, and SwissTargetPrediction for potential target prediction. The screening thresholds were TargetNet probability ≥ 90%, SEA probability ≥ 0.30, BATMAN-TCM score ≥ 80, and SwissTargetPrediction probability > 0.10. The results from all databases were combined. UniProt was used to unify protein names and gene symbols. The study species was limited, and duplicate targets were removed. The final target set was used as the potential target set of active compounds in vine tea.

### 2.3. Collection of Targets Related to Feline Intestinal Inflammation and Screening of Shared Targets

The keyword Intestinal Inflammation was used to search GeneCards, Open Targets, and OMIM for disease-related genes. This broad search term was intentionally used to capture inflammatory mechanisms shared across the heterogeneous spectrum of feline chronic enteropathies rather than to represent a single clinical entity such as feline inflammatory bowel disease. GeneCards records with a relevance score ≥2 were retained. Open Targets records with an overall association score ≥0.01 were retained. All related genes recorded in OMIM were included. Gene names from different databases were standardized, combined, and deduplicated. The resulting genes were treated as database-derived genes associated with a broadly defined intestinal-inflammation phenotype, not as experimentally established feline IBD-specific disease genes. The complete retrieved gene set is provided in Supplementary Data S2 (Table S2B). These databases mainly contain human genes. Therefore, the biomaRt 2.62.1 package in R was used for ortholog mapping. Human gene symbols were mapped to reported *Felis catus* orthologs, with one-to-one or high-confidence mappings and clear feline gene symbols prioritized. Records without reliable mapping were excluded. Because conservation of gene identity does not establish equivalent disease relevance or pharmacological regulation across species, this mapping was treated as a pragmatic cross-species approximation. Feline-specific disease genes remain underrepresented in these predominantly human-oriented resources; consequently, this strategy may fail to capture targets that are uniquely relevant to the feline chronic-enteropathy spectrum. A revision-stage mapping audit of the shared targets and the archived feline STRING identifiers is provided in Supplementary Data S2 (Table S2C). The potential targets of vine tea were then intersected with feline intestinal inflammation-related targets. The intersecting genes were defined as candidate shared targets for vine tea against feline intestinal inflammation. Venn diagrams for database targets and drug-disease shared targets were drawn with the VennDiagram 1.7.3 package.

### 2.4. Construction of the Vine Tea-Compound-Shared Target Network

The links between active compounds in vine tea and shared targets were organized. These data were imported into Cytoscape 3.9.1 to build the vine tea-active compound-target-feline intestinal inflammation network. In the network, nodes represented vine tea, active compounds, candidate targets, and the disease. Edges represented potential links between compounds and targets. Network Analyzer was used to calculate topological parameters, including degree, betweenness centrality, and closeness centrality. Node size and color depth were used to show network importance. Active compounds with possible major roles were identified according to degree values.

### 2.5. Construction of the PPI Network and Screening of Core Targets

The shared targets between vine tea and feline intestinal inflammation were imported into the STRING database. The species was set as Felis catus. The minimum interaction confidence score was set to 0.40. Isolated nodes without interactions were hidden. Protein–protein interaction data were downloaded and imported into Cytoscape 3.9.1 to build the PPI network. The CytoNCA plug-in was used to calculate betweenness centrality, closeness centrality, degree centrality, eigenvector centrality, local average connectivity, and network centrality. The median value of each index was used as the first screening threshold. Nodes that met the criteria were screened through several rounds of iteration.

### 2.6. GO Functional Enrichment and KEGG Pathway Enrichment Analysis

The clusterProfiler 4.6.2 package in R 4.2.1 was used for enrichment analysis. Felis catus annotation information was used as the background. Gene Ontology (GO) functional enrichment and Kyoto Encyclopedia of Genes and Genomes (KEGG) pathway enrichment were performed for shared targets. GO analysis included biological process, molecular function, and cellular component terms. The species code for KEGG analysis was set as fca. Both nominal *p* values and multiplicity-adjusted *p* values were retained. The Results distinguish terms/pathways with nominal *p* < 0.05 from those remaining significant at adjusted *p* < 0.05, and the complete values are reported in Supplementary Data S2. Representative terms were selected according to significance and the number of enriched genes. The results were visualized with bar plots, dot plots, and circle plots.

### 2.7. Construction of the Compound-Target-Pathway-Disease Network

The top 30 pathways with high significance and close links to intestinal inflammation were selected from the KEGG results. The links among active compounds, shared targets, significant pathways, and the disease were organized. These data were imported into Cytoscape 3.9.1 to build the vine tea-active compound-target-pathway-feline intestinal inflammation network. Degree values and network connections were used to analyze cooperation among compounds, targets, and pathways. Sankey plots or Sankey-bubble plots were used to show how core targets were distributed across inflammation, immune, and cell survival-related pathways.

### 2.8. Receptor Structure Collection, AlphaFold Prediction, and Molecular Docking

Ten representative candidate compounds (gallic acid, palmitic acid, emodin, cianidanol, cedrol, aromadendrin, taxifolin, quercetin, kaempferol, and kaempferol oxoanion) were cross-docked against the six core targets (IL6, TNF, IL1B, STAT3, CASP3, and TLR4), resulting in 60 compound-target combinations. The compounds were selected according to compound-target network topology and representation among the screened candidates, while the six receptors were the PPI-prioritized core targets. Three-dimensional ligand structures were downloaded from PubChem, hydrogenated, energy-minimized, and converted to PDBQT format. Feline CASP3, IL1B, IL6, TLR4, and TNF receptor models were obtained from AlphaFold DB, and the predicted STAT3 structure retained in the docking archive was used for STAT3. Receptor and ligand preparation was performed with PyMOL 3.1 and AutoDock Tools, including hydrogen addition and charge assignment. AutoDock Vina 1.2.7 with the vina scoring function was used for docking. The archived configuration files recorded exhaustiveness = 50, CPU = 4, random seed = 1000, grid spacing = 0.375 Å, and nine output poses per docking run. A receptor-specific grid box was used for each target; the exact structure identifiers, grid centers, and grid sizes are provided in Supplementary Data S1 (Table S1F). The best-ranked pose was used for the reported binding-energy matrix, and six IL6-compound complexes with favorable predicted docking scores were subsequently selected for structural visualization.

### 2.9. Molecular Dynamics Simulation

Representative ligand-receptor complexes with low binding energy and reasonable conformations were selected for 100 ns molecular dynamics simulation in GROMACS. Topologies were generated according to protein and small-molecule force fields. Each complex was placed in a periodic water box. Counterions were added to neutralize the system. Production simulation was performed after energy minimization and NVT and NPT equilibration. RMSD, RMSF, radius of gyration, solvent-accessible surface area, hydrogen bond number, and ligand-receptor distance were calculated from the trajectory. These parameters were used to evaluate system stability. The MM/PBSA method was used to calculate binding free energy during the equilibrium stage.

### 2.10. Generative Artificial Intelligence Use

Generative artificial intelligence (ChatGPT-5.6) was used during manuscript revision only for routine language editing, including grammar, spelling, punctuation, wording, and formatting adjustments. It was not used for study design, data collection, data generation, data analysis, interpretation of results, or figure generation, nor was it used to replace any of the original computational analyses. All language edits were reviewed and approved by the authors, who take full responsibility for the final manuscript.

## 3. Results

### 3.1. Screening of Candidate Compounds in Vine Tea and Targets Related to Feline Intestinal Inflammation

**Figure 1:**
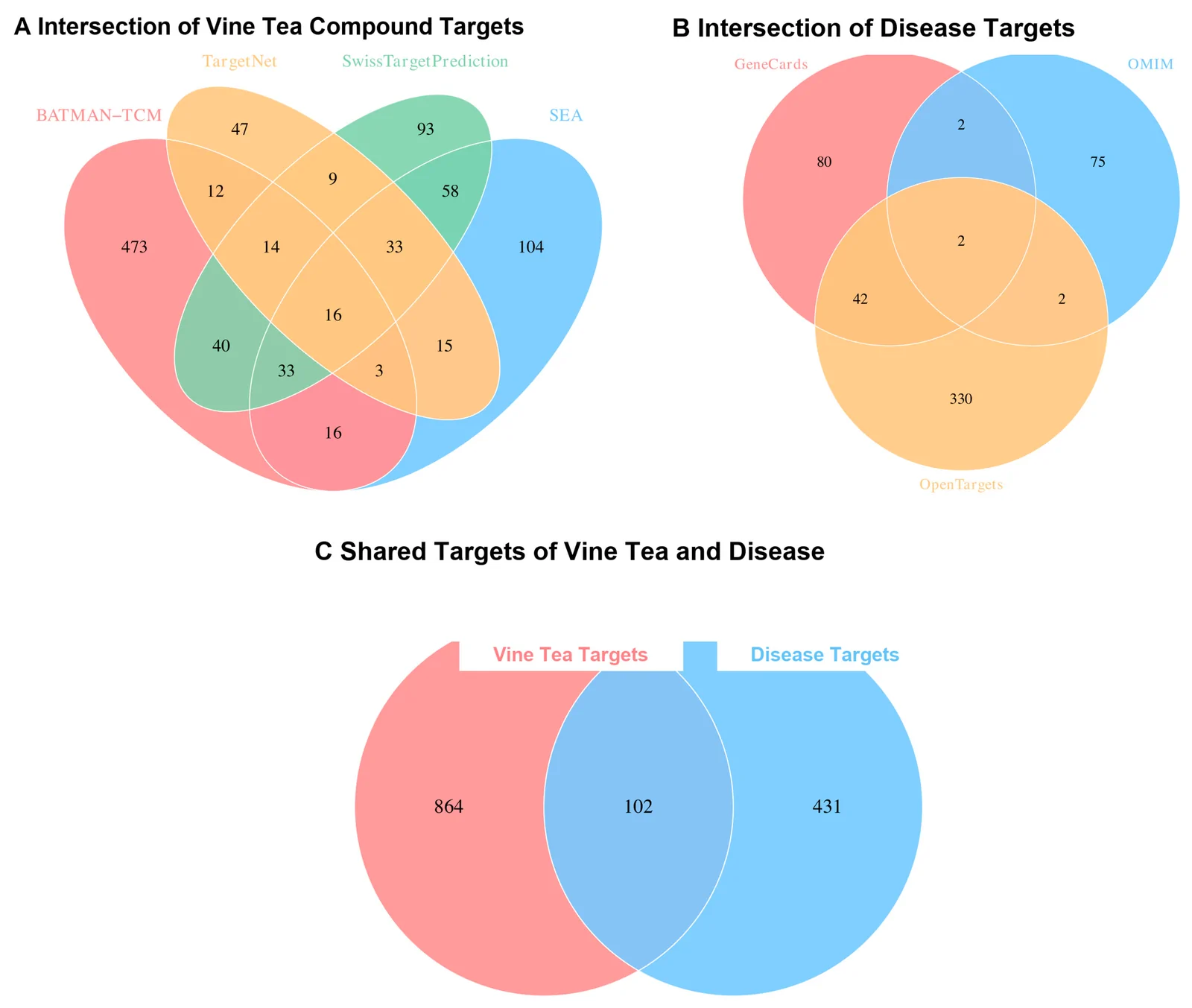
Screening results for vine tea compound targets, feline intestinal inflammation disease targets, and shared targets. (**A**): database intersection of vine tea compound targets. (**B**): database intersection of disease targets. (**C**): intersection of vine tea targets and disease targets.

After PubChem structural information was organized and SwissADME drug-likeness screening was completed, 17 candidate compounds were obtained from vine tea (*Ampelopsis grossedentata*, Xian Chi She Pu Tao). Target prediction results from BATMAN-TCM, SEA, TargetNet, and SwissTargetPrediction were then combined. After duplicate records were removed, 966 potential targets of vine tea were obtained. BATMAN-TCM, SwissTargetPrediction, SEA, and TargetNet provided 607, 296, 278, and 149 non-duplicate targets, respectively. These results showed a clear multi-target feature of active compounds in vine tea. The basic information for the active compounds and their predicted target counts is shown in Table 1. The target source counts and intersections across databases are shown in Figure 1A. The disease search retained 126 GeneCards records, 376 Open Targets records, and 81 OMIM records; after merging and deduplication, 533 unique database-derived intestinal-inflammation-associated genes were obtained. These genes represent a broad human-centric intestinal-inflammation phenotype rather than experimentally established feline IBD-specific genes. The complete disease-gene dataset and the revision-stage feline mapping audit are provided in Supplementary Data S2. The archived downstream workflow yielded 102 candidate shared targets for subsequent network analyses.

### 3.2. Construction of the PPI Network and Screening of Core Targets

**Figure 2:**
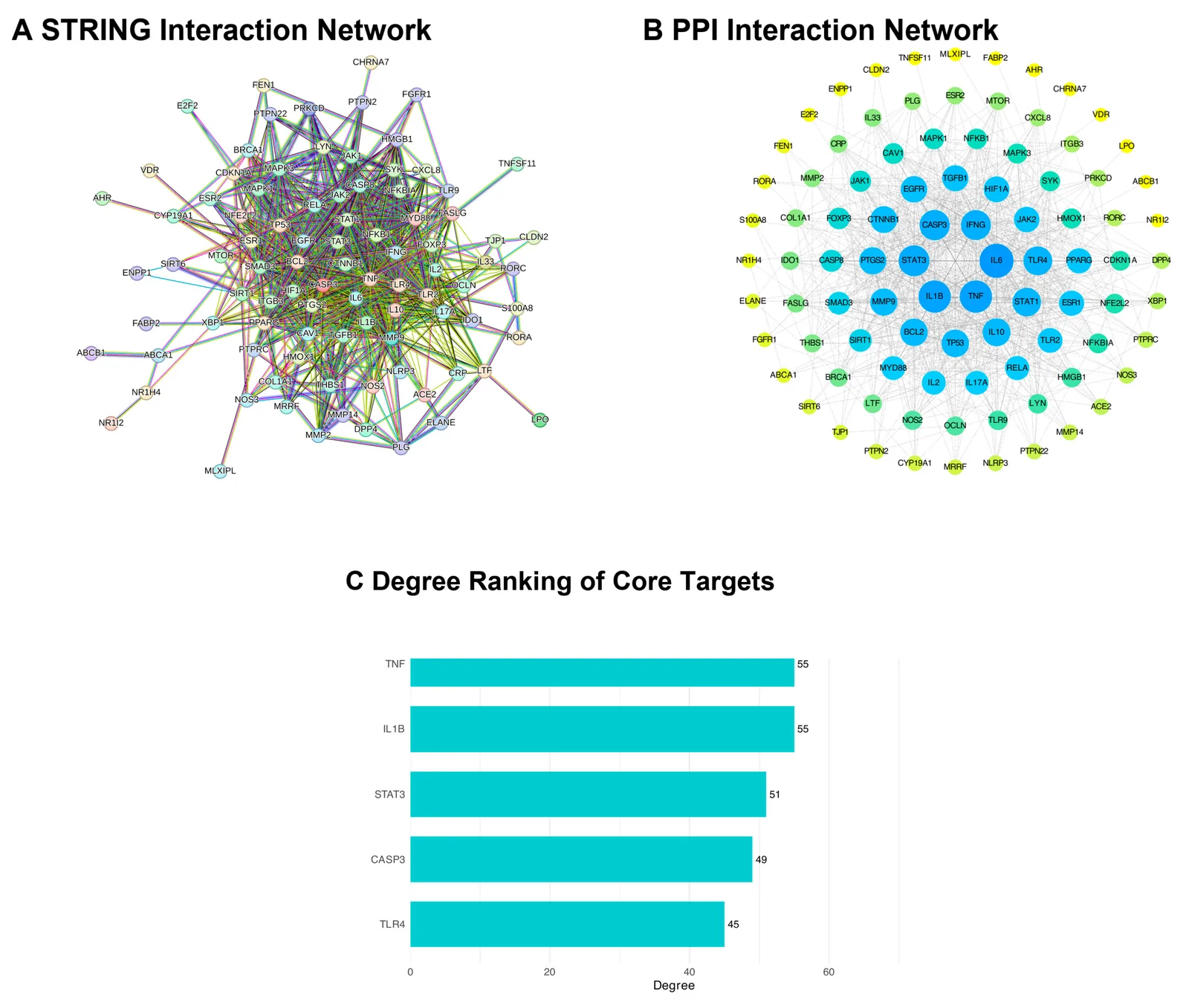
PPI network of shared targets and screening of core targets. (**A**): STRING interaction network. (**B**): Cytoscape PPI network. (**C**): Degree ranking of core targets.

The 102 shared targets were imported into STRING to build a protein–protein interaction (PPI) network. The network was then imported into Cytoscape for topological analysis. After isolated nodes were removed, the PPI network contained 92 nodes and 872 interaction edges. Several rounds of screening were performed with topological indexes, including Degree, Betweenness, and Closeness. IL6, TNF, IL1B, STAT3, CASP3, and TLR4 were finally identified as core targets. IL6 had the highest Degree value of 60. TNF and IL1B both had a Degree value of 55. The topological parameters of core targets are shown in Table 2. The PPI network structure and screening process are shown in Figure 2A–C. Within this archived PPI network, the six prioritized targets showed high centrality. A revision-stage audit of the feline mapping identifiers used for the shared-target set is provided in Supplementary Data S2 (Table S2C).

### 3.3. GO Functional Enrichment Analysis

**Figure 3:**
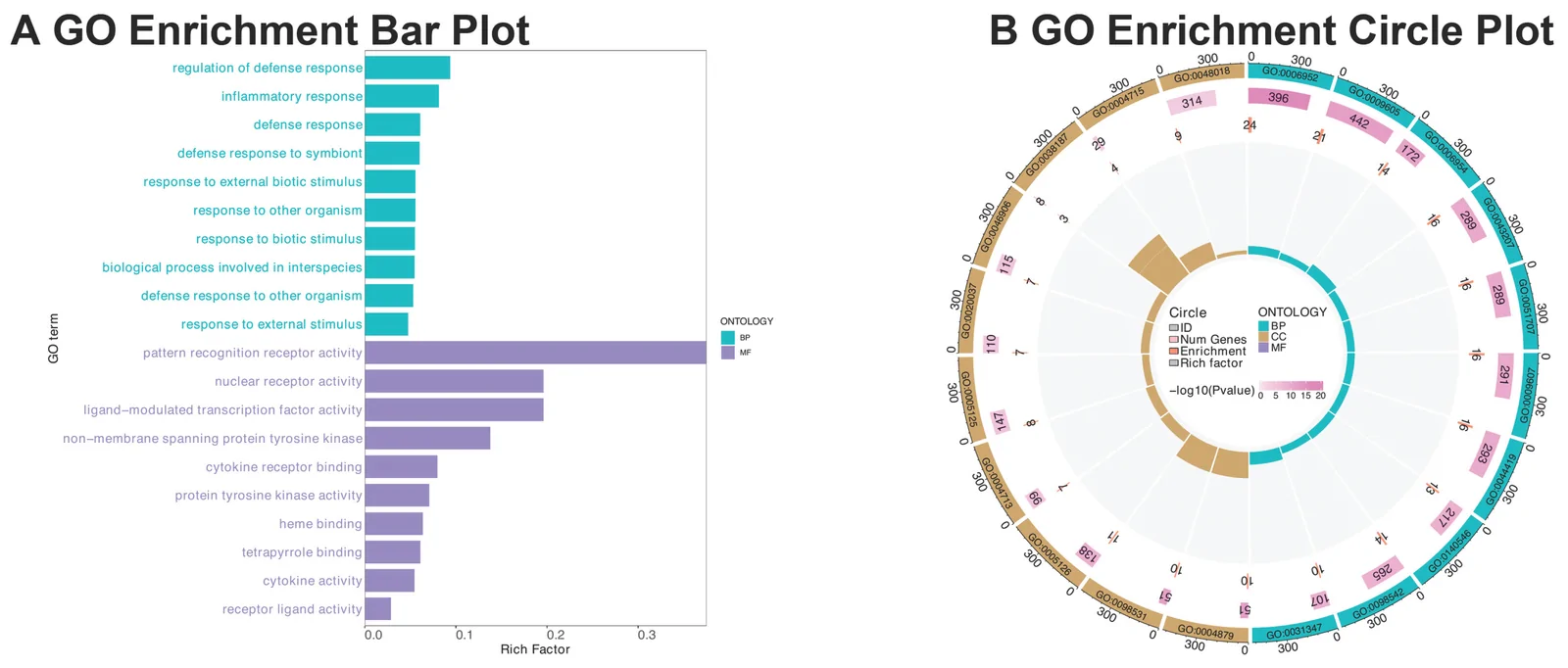
GO functional enrichment analysis of shared targets. (**A**): GO enrichment bar plot. (**B**): GO enrichment circle plot.

GO functional enrichment analysis was performed for the 102 candidate targets. At nominal *p* < 0.05, 195 terms were obtained, including 171 biological process (BP) terms and 24 molecular function (MF) terms; 107 of the 195 terms remained significant at adjusted *p* < 0.05. No cellular component (CC) term reached nominal *p* < 0.05. The BP terms mainly involved immune and inflammatory processes, such as defense response, response to external stimulus, inflammatory response, response to external biotic stimulus, and innate immune response. The MF terms mainly involved nuclear receptor activity, ligand-modulated transcription factor activity, cytokine receptor binding, protein tyrosine kinase activity, and cytokine activity. Representative terms are shown in Table 3. The overall enrichment distribution is shown in Figure 3A,B. The complete GO output, including nominal and adjusted *p* values, is provided in Supplementary Data S2.

### 3.4. KEGG Pathway Enrichment Analysis and Network Construction

**Figure 4:**
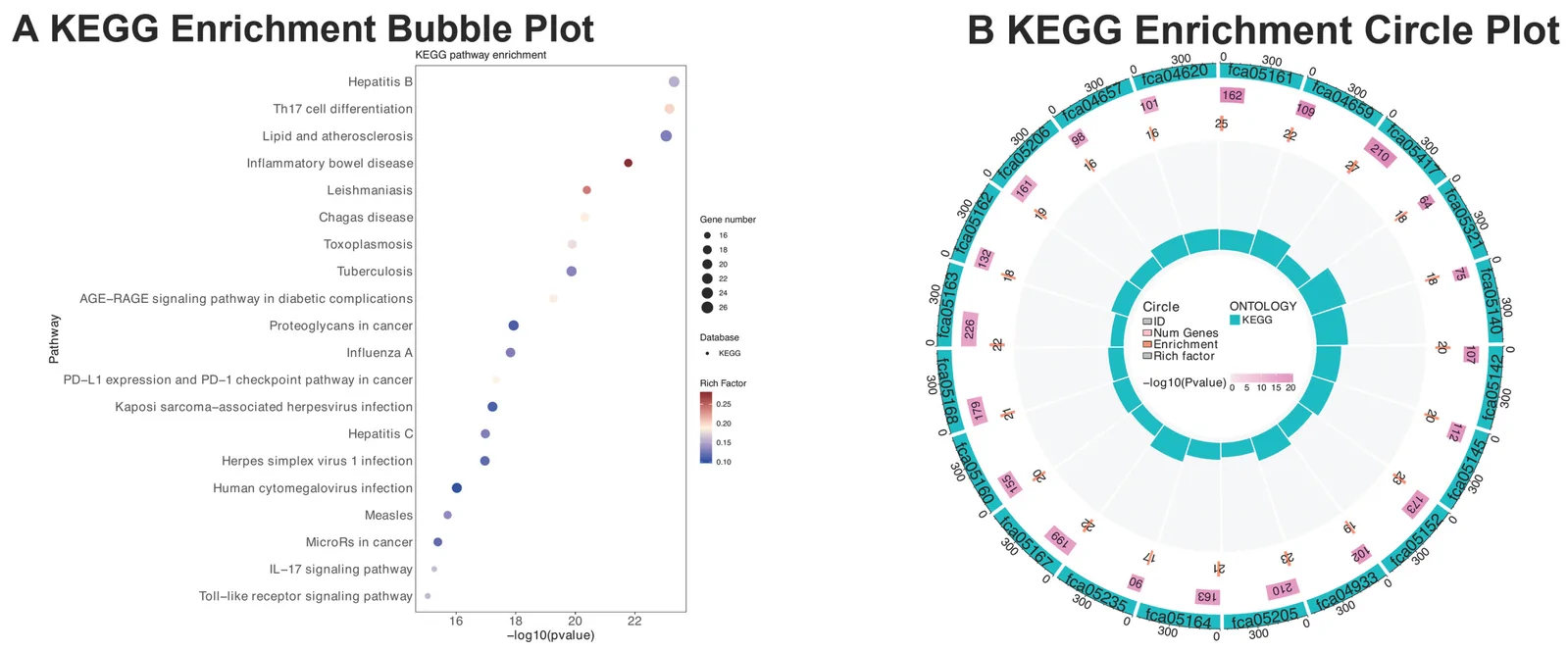

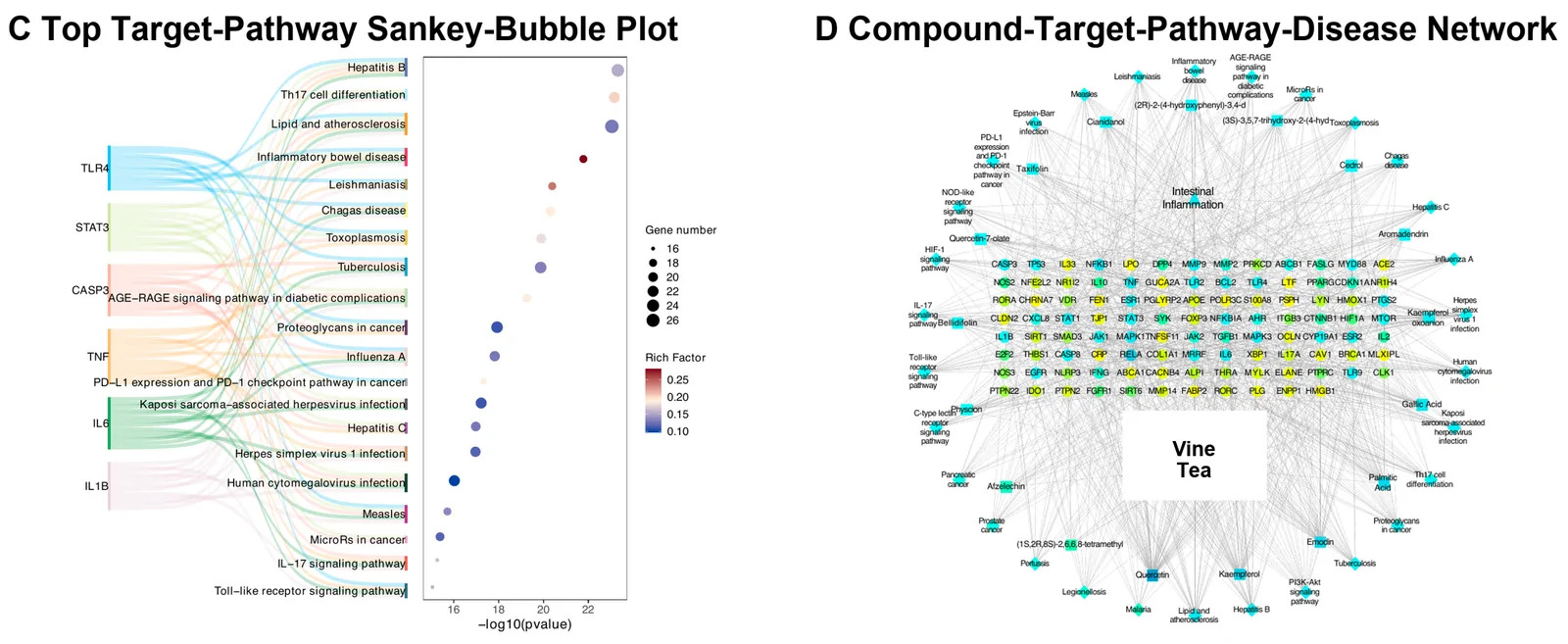
KEGG enrichment analysis of shared targets. (**A**): KEGG enrichment bubble plot. (**B**): KEGG enrichment circle plot. Relationship between top targets and top pathways and the compound-target-pathway-disease network. (**C**): Sankey-bubble plot of top targets and top pathways. (**D**): compound-target-pathway-disease network.

KEGG pathway enrichment analysis identified 173 pathways. Among them, 150 pathways had nominal *p* < 0.05, and 141 remained significant at adjusted *p* < 0.05. Pathways ranked highly by adjusted *p* value mainly included Hepatitis B, Th17 cell differentiation, Lipid and atherosclerosis, Inflammatory bowel disease, Leishmaniasis, Chagas disease, Toxoplasmosis, and Tuberculosis. Several pathways closely related to intestinal inflammation were also enriched, including Th17 cell differentiation, Inflammatory bowel disease, IL-17 signaling pathway, Toll-like receptor signaling pathway, NOD-like receptor signaling pathway, NF-kappa B signaling pathway, TNF signaling pathway, JAK-STAT signaling pathway, and PI3K-Akt signaling pathway. These computational enrichment results prioritize inflammation, innate immunity, and cell-survival-related pathways for subsequent experimental investigation rather than demonstrating pathway regulation by vine tea in cats. The top 12 pathways are shown in Table 4. The overall KEGG enrichment results are shown in Figure 4A,B, and the complete output is provided in Supplementary Data S2.

The top enriched pathways and their related targets were then selected to build the vine tea-compound-target-pathway-disease network. After database source nodes were removed, the network contained 151 nodes and 1025 edges. It included 17 active compounds, 102 shared targets, 30 significant pathways, the vine tea node, and the feline intestinal inflammation disease node. In this network, one active compound could act on several targets. The same target could also take part in several signaling pathways. These network relationships computationally support a multi-component, multi-target, and multi-pathway hypothesis that requires feline-specific experimental validation. The relationship between top targets and top pathways and the compound-target-pathway-disease network are shown in Figure 4C,D.

### 3.5. Molecular Docking Validation

**Figure 5:**
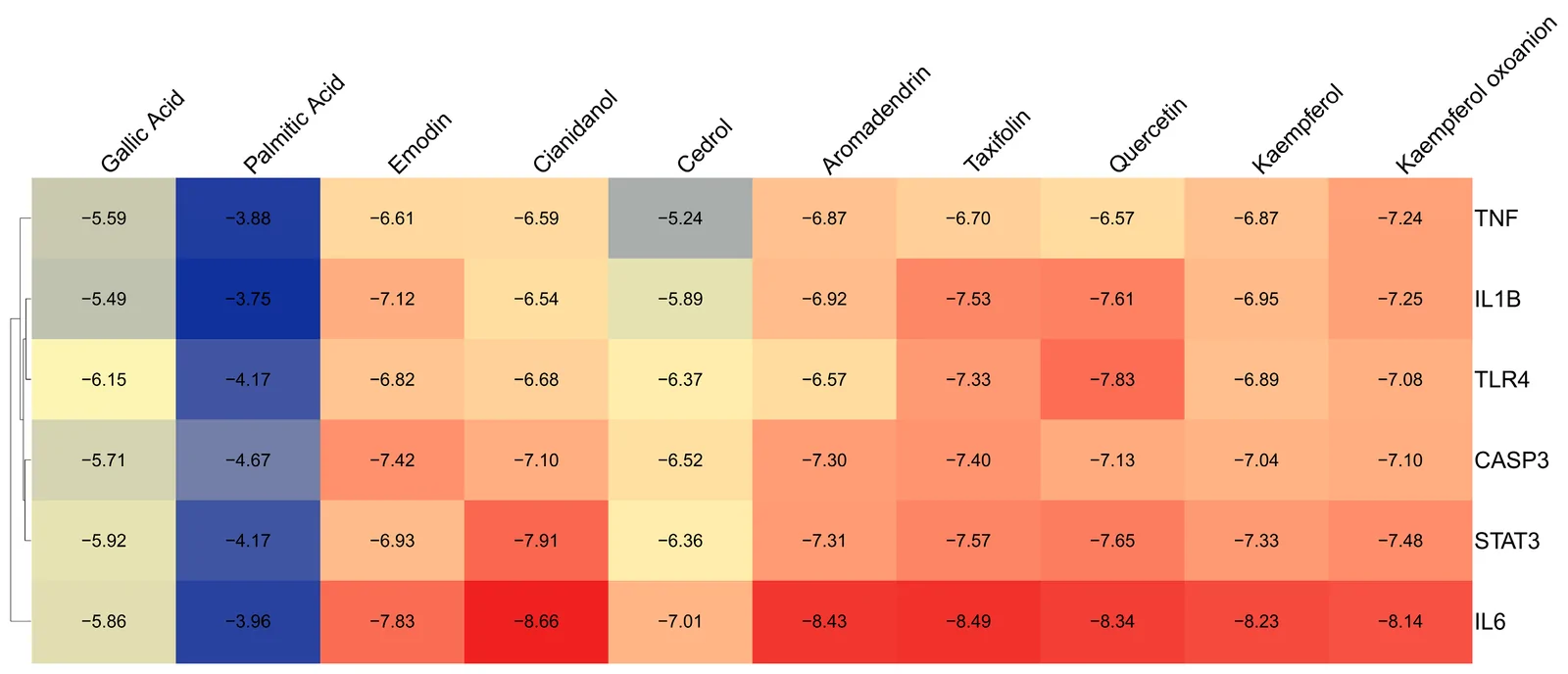

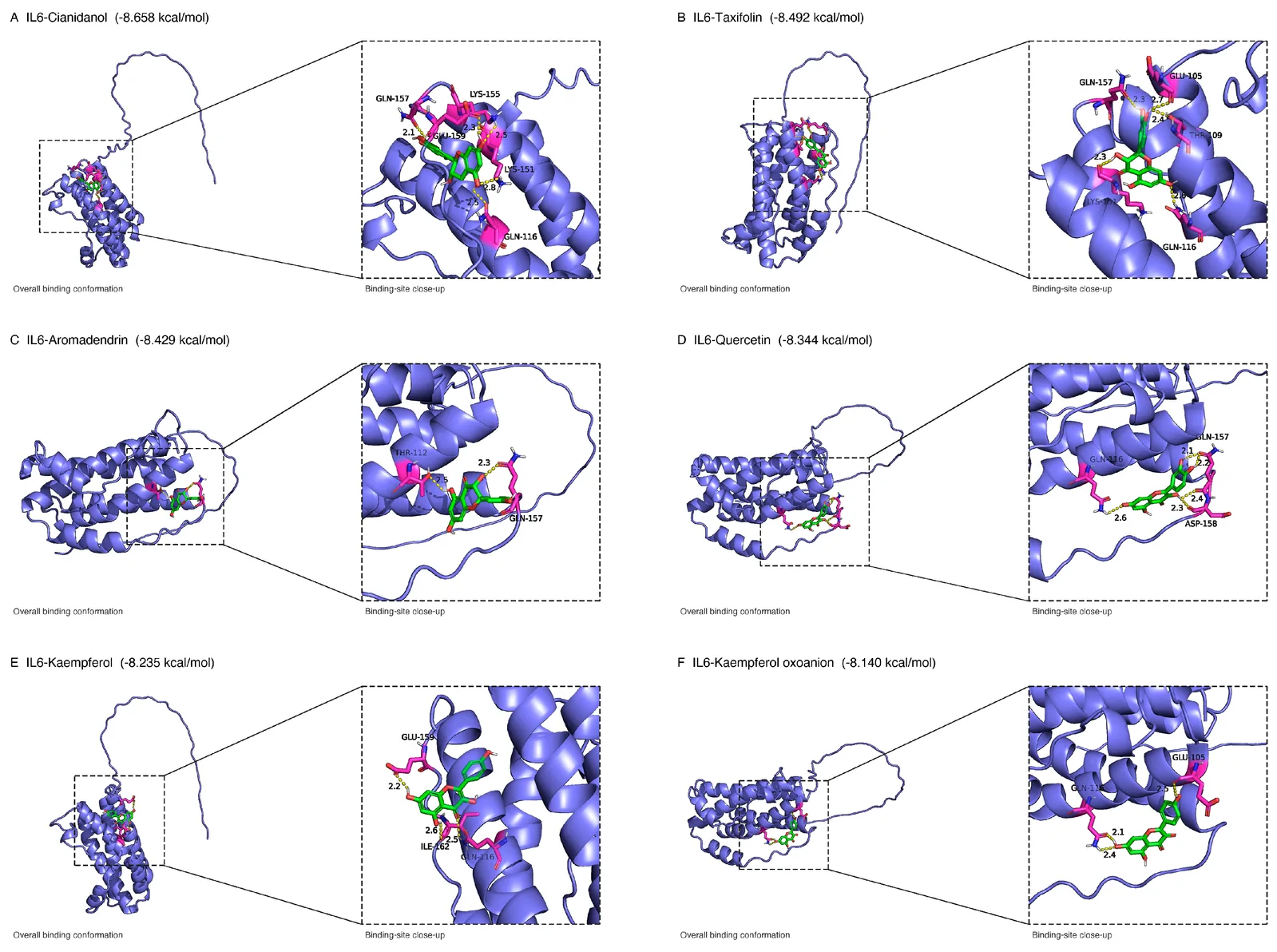
Heatmap of molecular docking binding energies between candidate compounds and core targets. Docking conformations of IL6 with six candidate compounds showing favorable predicted docking scores. (**A**–**F**): Cianidanol, Taxifolin, Aromadendrin, Quercetin, Kaempferol, and Kaempferol oxoanion.

Molecular docking comprised 10 representative compounds cross-docked against six core targets, yielding 60 combinations. This analysis was used to prioritize structurally plausible candidate interactions rather than to demonstrate experimental binding or efficacy. Binding energies ≤−5.0 kcal/mol and ≤−7.5 kcal/mol were used as heuristic cutoffs for favorable and strong predicted docking, respectively. Among them, 54 pairs had binding energies ≤−5.0 kcal/mol, and 13 pairs had binding energies ≤−7.5 kcal/mol. IL6 showed favorable predicted docking energies with several candidate compounds. The binding energies of IL6-Cianidanol, IL6-Taxifolin, IL6-Aromadendrin, IL6-Quercetin, IL6-Kaempferol, and IL6-Kaempferol oxoanion were −8.658, −8.492, −8.429, −8.344, −8.235, and −8.140 kcal/mol, respectively. STAT3-Cianidanol (−7.905 kcal/mol), TLR4-Quercetin (−7.829 kcal/mol), and IL1B-Quercetin (−7.613 kcal/mol) also showed favorable predicted docking energies. The distribution and values of binding energy for all pairs are shown in Figure 5. The docking conformations of six IL6-compound complexes with favorable predicted docking scores are shown in Figure 5A–F.

### 3.6. Molecular Dynamics Simulation

**Figure 6:**
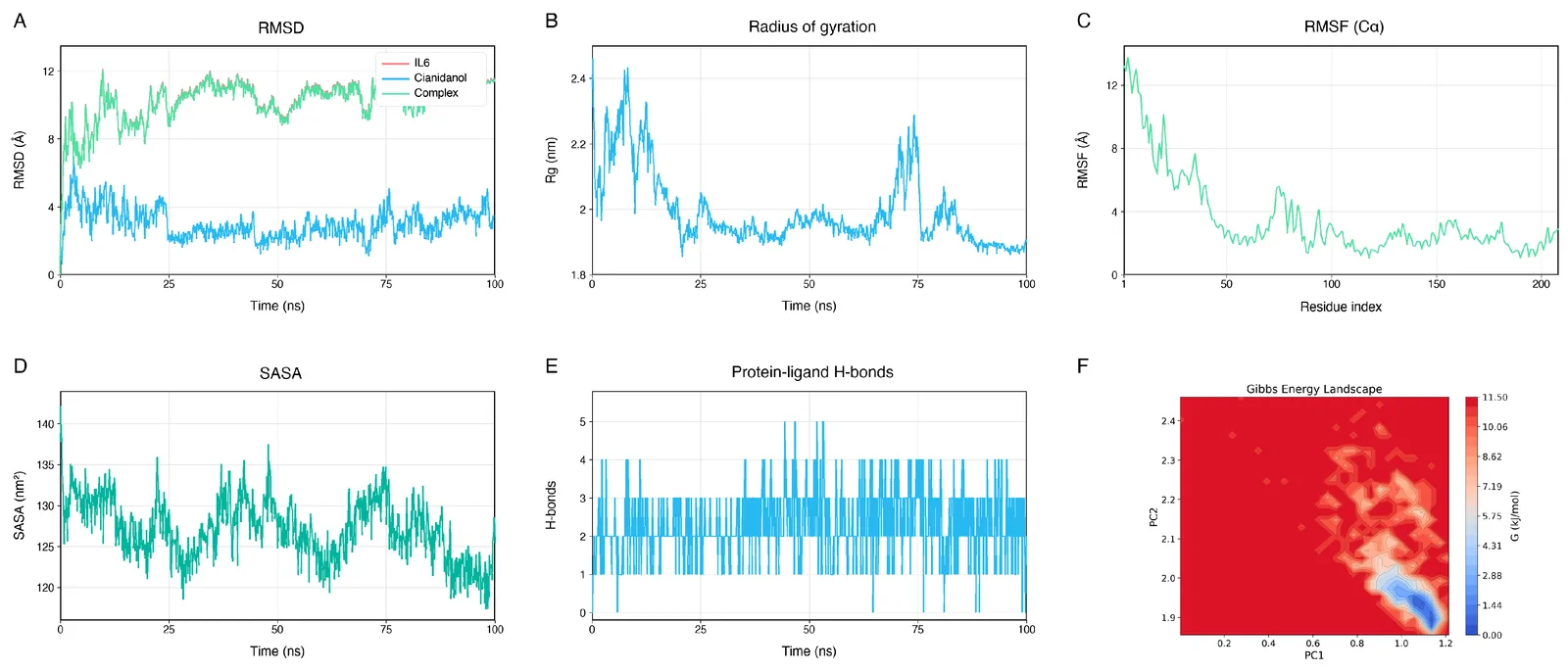
Molecular dynamics simulation analysis of the IL6-Cianidanol complex. (**A**): RMSD curves of the protein, ligand, and complex. (**B**): radius of gyration (Rg). (**C**): residue RMSF. (**D**): SASA. (**E**): number of protein-ligand hydrogen bonds. (**F**): two-dimensional Gibbs free energy landscape.

Based on the molecular docking results, the IL6-Cianidanol complex with the lowest binding energy was selected for 100 ns molecular dynamics simulation. This simulation was used to evaluate the dynamic stability of the protein-ligand complex. Figure 6A shows that the RMSD curves of the protein, ligand, and complex increased during the early stage and then entered a fluctuation plateau. The mean RMSD values of the protein and complex were 10.24 +/− 1.26 Å and 10.18 +/− 1.25 Å, respectively. The mean ligand RMSD was 2.97 +/− 0.86 Å, and the final-frame value was 4.40 Å. Within the simulation, these values suggested relative stability of the ligand pose in the modeled IL6 binding region. Figure 6B,D show that the mean Rg was 1.991 +/− 0.112 nm, and the final-frame value was 1.899 nm. The mean SASA was 126.9 +/− 3.7 nm^2^, and the final-frame value was 125.4 nm^2^. These results indicated no sustained drift in overall compactness or surface exposure during the simulated trajectory. Figure 6C shows that the mean C-alpha residue fluctuation was 3.57 Å. The maximum fluctuation was 13.75 Å, and the largest fluctuation was concentrated near the second residue in the N-terminal region. Figure 6E shows that the mean number of protein-ligand hydrogen bonds was 2.21 +/− 0.84, and the final-frame value was 2. This result indicated persistent modeled hydrogen-bond contacts during the trajectory. Figure 6F shows that the low-free-energy conformations were mainly located at PC1 = 1.0–1.2 and PC2 = 1.9–2.0. This result indicated a dominant low-energy conformational cluster within the simulated conformational space; it does not establish experimental target engagement.

## 4. Discussion

This study used network pharmacology, molecular docking, and molecular dynamics simulation to characterize predicted molecular associations between vine tea constituents and feline intestinal inflammation. The analysis covered chemical components, targets, pathways, and structural stability. The study identified 17 candidate compounds, 966 potential targets, and 102 shared drug-disease targets. It also built a connected regulatory network with multiple components, targets, and pathways. This feature fits the disease features of FCE. FCE involves mucosal immune imbalance, epithelial barrier injury, microbiota changes, and metabolic disorder. Previous studies found that cats with inflammatory bowel disease and low-grade small-cell lymphoma show reduced fecal bacterial diversity and changed levels of specific bacteria. The dysbiosis index can separate most cats with chronic enteropathy from healthy cats [25,26]. Disease-affected cats also show a clear decrease in microbial tryptophan-derived indole metabolites. Intestinal S100/calgranulin expression is related to the level of local inflammation [27,28]. Therefore, the broad target profile and immune-inflammatory enrichment results define a computationally inferred network connecting inflammatory signaling, mucosal-barrier processes, and microbiota-related pathways; they do not demonstrate that vine tea regulates these processes in cats.

Among the screened active compounds, quercetin, kaempferol, taxifolin, aromadendrin, gallic acid, and emodin had high target connectivity. These compounds represent candidate constituents underlying the predicted network associations of vine tea. Quercetin can inhibit TNF-induced Akt activation and the recruitment of the NF-kappa B transcription complex to inflammatory gene promoters. It can also reduce inflammatory mediator expression after intestinal metabolic conversion [29,30]. Kaempferol can reduce DSS-induced colonic mucosal injury and improve goblet cell function [31]. Taxifolin can reduce IL-6, IL-1beta, and TNF-alpha. It can also regulate the gut microbiota and increase butyrate levels. These effects indicate that taxifolin may support both immune regulation and microbiota improvement [32]. Gallic acid can inhibit p65 NF-kappa B and IL-6/STAT3 activation [33]. Emodin can relieve experimental colitis by regulating flagellin/TLR5/MyD88/NF-kappa B signaling. It can also activate PPARgamma and restore the low-oxygen environment in the colon [34,35]. These findings agree with the present result that flavonoids and polyphenols connect with several inflammatory targets and pathways. The number of network targets does not equal in vivo efficacy. Compound content, intestinal conversion, bioavailability, and feline metabolic features can all affect the real contribution. Accordingly, the network should be interpreted as a hypothesis that several constituents may contribute complementary associations at different steps, not as evidence of an in vivo cooperative therapeutic effect.

PPI topological analysis identified IL6, TNF, IL1B, STAT3, CASP3, and TLR4 as core targets. This topology prioritizes cytokine amplification, pattern recognition, T-cell differentiation, and epithelial cell fate as candidate processes for subsequent validation. IL-6 trans-signaling can activate STAT3 and anti-apoptotic molecules. It can increase the resistance of mucosal T cells to apoptosis and maintain chronic intestinal inflammation [36]. TNF is a major driver of the intestinal mucosal inflammatory cascade. Anti-TNF therapy can reduce disease activity in some patients with Crohn’s disease. This evidence shows the therapeutic value of this target [37]. IL-1beta not only promotes granulocyte recruitment. It also increases the accumulation and survival of IL-17A-secreting innate lymphoid cells and Th17 cells [38]. STAT3 directly takes part in the formation of inflammatory helper T cells and regulates Th17-related gene expression [39]. The high centrality of CASP3 indicates that epithelial cell apoptosis may be involved in disease progression. At the same time, intestinal epithelial cell death does not follow only one pathway. Caspase-8 deficiency can induce TNF-dependent necroptosis and terminal ileitis [40]. In the present in silico framework, these processes provide testable hypotheses concerning inflammatory signaling, epithelial injury, and cell-death/repair balance; no reduction in barrier damage by vine tea was directly demonstrated.

GO analysis was mainly enriched in defense response, response to external stimulus, inflammatory response, innate immune response, and cytokine receptor binding. KEGG analysis highlighted Th17 cell differentiation, IL-17, Toll-like receptor, NOD-like receptor, NF-kappa B, TNF, JAK-STAT, and PI3K-Akt pathways. These enrichment results prioritize links between innate recognition and adaptive immunity as candidate mechanisms. TLR4/MyD88 signaling takes part in inflammatory cell recruitment after injury and limits bacterial translocation. However, basal stimulation of TLRs by normal commensal bacteria also helps maintain epithelial balance. Therefore, this pathway is more about restoring a proper response than blocking all signaling [41,42]. NOD2 variants are linked to Crohn’s disease susceptibility. This link further shows that abnormal intracellular microbial recognition can drive a persistent NF-kappa B response [43]. IL-23 can promote both innate immune-driven and T cell-mediated enteritis. It can also maintain colitis through IL-17 and IL-6 [44,45]. RORgammat controls the Th17 differentiation program. STAT3 coordinates Th17/Treg balance, T-cell proliferation, and T-cell survival [46,47]. The PI3K-Akt pathway depends on cell type and timing. In monocytes, it can limit excessive LPS-induced MAPK, NF-kappa B, and inflammatory mediator expression [48]. Therefore, the enriched pathways can be viewed as a computational network linking microbial recognition, cytokine release, STAT3/Th17 amplification, and epithelial injury. Whether vine tea constituents modulate these nodes sufficiently to alter this network in cats remains to be experimentally determined.

The molecular docking results provided structural plausibility for selected network-predicted interactions. Most candidate pairs showed low predicted binding energy. IL6 showed favorable predicted docking energies with Cianidanol, Taxifolin, Aromadendrin, Quercetin, Kaempferol, and Kaempferol oxoanion, which prioritizes IL6 for experimental binding studies but does not establish affinity or target engagement. STAT3-Cianidanol, TLR4-Quercetin, and IL1B-Quercetin also showed favorable predicted docking energies. These results agree with experimental studies showing that several flavonoids can affect cytokines, NF-kappa B, and STAT3 signaling at the same time. Quercetin can also reduce endoplasmic reticulum stress and epithelial cell apoptosis [49]. IL-6 can upregulate claudin-2 through the MEK/ERK and PI3K pathways. It can also increase tight junction permeability in intestinal epithelial cells [50]. If these interactions are experimentally confirmed at biologically relevant exposure, IL6-related signaling and barrier permeability would be appropriate downstream endpoints to test; the present docking results alone do not demonstrate either effect. Docking binding energy does not directly equal the true binding constant or in vivo efficacy. However, it can help select component-target pairs from a complex network for later validation.

Based on docking screening, this study selected the IL6-Cianidanol complex with the lowest binding energy for 100 ns molecular dynamics simulation. The protein and complex RMSD values entered a stable plateau after early adjustment. The ligand RMSD remained at a low level. Rg and SASA showed limited overall fluctuation. These results indicate that the compactness and solvent exposure of the complex did not show continuous drift. Large C-alpha residue fluctuations were mainly located in the local N-terminal region. The protein-ligand complex maintained about two hydrogen bonds on average. The two-dimensional Gibbs free energy landscape formed a concentrated low-energy conformational cluster. Together, these simulation descriptors indicate relative conformational stability of the modeled IL6-Cianidanol complex over the sampled trajectory; they do not demonstrate experimental binding, target engagement, or in vivo activity. The absolute RMSD values of the protein and complex were high. This pattern may be related to flexible regions and overall conformational adjustment. However, the stable ligand site, converged Rg and SASA, and dominant low-energy cluster support further experimental testing of this complex. Future work can combine binding free energy decomposition, key residue mutation, and surface plasmon resonance. These methods can clarify the true binding site and affinity.

An important limitation is the cross-species nature of several computational resources used in this study. Compound-target predictions and disease-associated genes were derived largely from human-oriented databases and then interpreted through feline ortholog mapping and species-restricted network analysis. Orthologous gene identity does not guarantee equivalent target regulation, pharmacological sensitivity, or disease relevance between humans and cats. Cats also show species-specific xenobiotic disposition, including reduced or substrate-dependent glucuronidation capacity, species-dependent cytochrome P450 activity, and other differences in xenobiotic metabolism [51,52]. Feline absorption, distribution, metabolism, and excretion data are unavailable for most candidate flavonoids evaluated here. Therefore, the predicted compound-target relationships implicitly assume that sufficient intestinal or systemic exposure can be achieved in cats, an assumption that remains untested, and the revision-stage orthology/mapping audit should be interpreted as a cross-species approximation rather than proof of feline functional equivalence.

A second translational limitation concerns the biological and product context. DSS-, TNBS-, and other experimentally induced rodent colitis studies cited above were used only to provide mechanistic context; these acute or chemically induced models do not fully reproduce the chronic, heterogeneous, spontaneously occurring inflammatory enteropathies observed in cats. In addition, vine tea is not a standardized veterinary preparation. Its phytochemical composition can vary with harvest conditions, geographic origin, processing, storage, and extraction procedures [10,53], and no study-specific vine tea batch was chemically quantified here. Consequently, the concentrations of the candidate compounds in practical preparations, the dose required to achieve the predicted exposure, and safety margins in cats cannot be inferred from the present analysis. The current findings should therefore be considered hypothesis-generating rather than a basis for veterinary dosing or clinical use.

In summary, this study generated a computational framework that prioritizes IL6, TNF, IL1B, STAT3, TLR4, and CASP3 together with TLR/NOD, NF-kappa B, TNF, JAK-STAT, Th17/IL-17, and PI3K-Akt pathways for subsequent feline-specific investigation. Several flavonoids and phenolic acids were connected to these targets in the network, and the IL6-Cianidanol pair emerged as a representative candidate interaction from docking and simulation. These results do not establish anti-inflammatory efficacy, target regulation, or therapeutic benefit in cats. The next step should test target engagement, cytokine signaling, tight-junction proteins, apoptosis, compound exposure, and safety in feline intestinal epithelial cells, organoids, ex vivo tissues, animal models, or clinical samples.

## 5. Conclusions

This entirely in silico study integrated network pharmacology, molecular docking, and molecular dynamics simulation to prioritize candidate molecular associations between vine tea constituents and feline intestinal inflammation. Seventeen candidate compounds and 966 predicted targets were identified, and 102 candidate shared targets were carried forward in the archived workflow. Network topology prioritized IL6, TNF, IL1B, STAT3, CASP3, and TLR4, while GO and KEGG enrichment linked the shared-target set to inflammatory response, cytokine activity, Th17 cell differentiation, and the IL-17, Toll-like receptor, NOD-like receptor, NF-kappa B, TNF, JAK-STAT, and PI3K-Akt pathways. Molecular docking further prioritized several predicted IL6 interactions, with the IL6-Cianidanol pair showing the lowest docking energy among the tested combinations, and the subsequent 100 ns simulation indicated relative stability of this modeled complex. Taken together, these analyses provide a hypothesis-generating framework for selecting compounds, targets, and pathways for feline-specific experimental testing; they do not demonstrate that vine tea attenuates intestinal inflammation or produces therapeutic benefit in cats. Experimental studies are required to establish target engagement, feline bioavailability and pharmacokinetics, effective exposure, safety, and biological relevance before vine tea or its constituents can be considered for veterinary application.

## Figures and Tables

**Table 1:** Candidate compounds retained from vine tea and their predicted target counts.

CID	Candidate Compound	Target Count	Molecular Formula	Molecular Weight	XLogP
985	Palmitic Acid	416	C16H32O2	256.42	6.4
5280343	Quercetin	314	C15H10O7	302.23	1.5
5280863	Kaempferol	201	C15H10O6	286.24	1.9
122850	Aromadendrin	195	C15H12O6	288.25	1.8
3220	Emodin	169	C15H10O5	270.24	2.7
370	Gallic Acid	163	C7H6O5	170.12	0.7
25202062	Kaempferol oxoanion	154	C15H9O6-	285.23	2.5
439533	Taxifolin	148	C15H12O7	304.25	1.5
118855455	(3S)-3,5,7-trihydroxy-2-(4-hydroxyphenyl)-2,3-dihydrochromen-4-one	145	C15H12O6	288.25	1.8
65575	Cedrol	137	C15H26O	222.37	3.9
9064	Cianidanol	122	C15H14O6	290.27	0.4
9943440	(2R)-2-(4-hydroxyphenyl)-3,4-dihydro-2H-chromene-3,5,7-triol	115	C15H14O5	274.27	0.7
46906036	Quercetin-7-olate	111	C15H9O7-	301.23	2.2
442154	Afzelechin	109	C15H14O5	274.27	0.7
10639	Physcion	101	C16H12O5	284.26	3.0
5281623	Bellidifolin	95	C14H10O6	274.22	2.4
44123431	(1S,2R,8S)-2,6,6,8-tetramethyltricyclo[5.3.1.01,5]undecan-8-ol	79	C15H26O	222.37	3.9

**Table 2:** Topological parameters of core targets screened from the PPI network.

Core Target	Degree	Betweenness	Closeness	LAC	Network
IL6	60	639.781	0.734	22.00	55.11
TNF	55	563.766	0.705	22.36	48.02
IL1B	55	428.632	0.705	22.15	48.95
STAT3	51	346.212	0.674	23.14	44.63
CASP3	49	299.758	0.669	23.47	41.94
TLR4	45	330.245	0.641	21.69	36.83

**Table 3:** Representative significant terms from GO enrichment analysis.

Category	GO ID	Description	GeneRatio	Count	P.adjust
BP	GO:0006952	defense response	24/94	24	1.55 × 10^−13^
BP	GO:0009605	response to external stimulus	21/94	21	1.11 × 10^−9^
BP	GO:0006954	inflammatory response	14/94	14	6.10 × 10^−9^
BP	GO:0043207	response to external biotic stimulus	16/94	16	3.09 × 10^−8^
BP	GO:0051707	response to other organisms	16/94	16	3.09 × 10^−8^
MF	GO:0004879	nuclear receptor activity	10/99	10	6.53 × 10^−10^
MF	GO:0098531	ligand-modulated transcription factor activity	10/99	10	6.53 × 10^−10^
MF	GO:0005126	cytokine receptor binding	11/99	11	7.36 × 10^−7^
MF	GO:0004713	protein tyrosine kinase activity	7/99	7	1.25 × 10^−3^
MF	GO:0005125	cytokine activity	8/99	8	1.43 × 10^−3^

**Table 4:** Top 12 significant pathways from KEGG enrichment analysis.

KEGG ID	Pathway Name	GeneRatio	Count	P.adjust
fca05161	Hepatitis B	25/96	25	1.06 × 10^−21^
fca04659	Th17 cell differentiation	22/96	22	1.06 × 10^−21^
fca05417	Lipid and atherosclerosis	27/96	27	1.06 × 10^−21^
fca05321	Inflammatory bowel disease	18/96	18	1.49 × 10^−20^
fca05140	Leishmaniasis	18/96	18	2.88 × 10^−19^
fca05142	Chagas disease	20/96	20	2.88 × 10^−19^
fca05145	Toxoplasmosis	20/96	20	5.99 × 10^−19^
fca05152	Tuberculosis	23/96	23	5.99 × 10^−19^
fca04933	AGE-RAGE signaling pathway in diabetic complications	19/96	19	2.17 × 10^−18^
fca05205	Proteoglycans in cancer	23/96	23	4.22 × 10^−17^
fca05164	Influenza A	21/96	21	4.87 × 10^−17^
fca05235	PD-L1 expression and PD-1 checkpoint pathway in cancer	17/96	17	1.36 × 10^−16^

## Data Availability

The original contributions presented in this study are included in the article/Supplementary Materials. Further inquiries can be directed to the corresponding author.

## Supplementary Materials

The following supporting information can be downloaded at: https://www.mdpi.com/article/10.3390/vetsci13090895/s1. The datasets supporting the findings of this study are provided in the article and its Supplementary Materials. Supplementary Data S1 contains the complete compound-screening audit, compound-provenance information, database-specific compound-target prediction dataset, molecular-docking parameters, and all 60 docking scores. Supplementary Data S2 contains the complete database-derived intestinal-inflammation gene set, the revision-stage feline mapping audit for the shared-target set, and the complete GO and KEGG enrichment outputs with nominal and adjusted *p* values.
