# Supplementary material for: Network Pharmacology and Molecular Simulation Reveal Potential Targets and Pathways of Vine Tea in Feline Intestinal Inflammation
Source: Vet Sci. 2026 Aug 31;13(9):895. doi: 10.3390/vetsci13090895 (PMC13611877; doi:10.3390/vetsci13090895)
Supplement: Supplementary file 1 [file vetsci-13-00895-s001.zip › Supplementary S2.pdf]

Supplementary Data S2

Complete Disease-Gene Audit, Revision-Stage Human-to-Feline Mapping Audit, and GO/KEGG Enrichment Results for the 102 Shared Targets

Disease-gene scope: The search term “Intestinal Inflammation” was used as a broad phenotype rather than as a synonym for feline inflammatory bowel disease. The archived disease-target dataset contains human-oriented database records from GeneCards, Open Targets, and OMIM. These records should therefore be interpreted as database-derived genes associated with a broadly defined intestinal-inflammation phenotype, not as experimentally established feline IBD-specific genes.

Archived retrieval thresholds: GeneCards relevance score ≥2; Open Targets overall/global association score ≥0.01; all genes retained in the archived OMIM filtered result. After source merging and deduplication, 533 unique disease-associated gene symbols were retained. Their intersection with the vine-tea predicted-target set yielded 102 shared targets.

Enrichment-data scope: The complete archived GO and KEGG result files are included in this Supplementary Data S2. Both nominal P values and multiplicity-adjusted P values are retained so that the reported term/pathway counts can be interpreted without conflating the two thresholds. GO analysis used Felis catus annotation/background information, and KEGG pathway identifiers use the feline species code “fca”.

Table S2A. Disease-target retrieval summary and filtering rules.

Source / stage	Search scope	Filtering rule	Unique genes
GeneCards	Intestinal Inflammation	Relevance score ≥2	126
Open Targets	MONDO_0030308 / intestinal inflammation archive	Overall/global score ≥0.01	376
OMIM	Archived intestinal-inflammation-related gene map	All genes in filtered result	81
Merged nonredundant set	—	Deduplicated across sources	533
Drug–disease shared set	—	Intersection with 966 predicted vine-tea targets	102

Forty-eight disease-gene symbols were supported by more than one of the three source databases in the archived merged file.

Table S2B. Complete database-derived intestinal-inflammation gene set (n = 533).

No.	Gene symbol	Source database(s)	GeneCards relevance score	Open Targets overall score	OMIM	In 102 shared targets
1	1D	OMIM			Yes	No
2	A4GNT	OpenTargets		0.0341344	No	No
3	AAT4	OMIM			Yes	No
4	AAT7	OMIM			Yes	No
5	ABCA1	OpenTargets		0.0342305	No	Yes
6	ABCB1	GeneCards; OpenTargets	3.0554	0.0395598	No	Yes
7	ABCB4	OpenTargets		0.0354205	No	No
8	ABCC4	OpenTargets		0.0369728	No	No
9	ABCG1	OpenTargets		0.0317765	No	No
10	ABCG8	OpenTargets		0.0351175	No	No
11	ABL1	OpenTargets		0.0318652	No	No
12	ACE2	GeneCards; OpenTargets	4.0283	0.0305199	No	Yes
13	ACTA3	OMIM			Yes	No
14	ACTG2	OMIM			Yes	No
15	ACVRLK3	OMIM			Yes	No
16	ADAM17	OpenTargets		0.0309338	No	No
17	ADAMDEC1	OpenTargets		0.036204	No	No
18	ADAMTS18	OpenTargets		0.0312738	No	No
19	AGR2	OpenTargets		0.0425257	No	No
20	AHR	GeneCards; OpenTargets	3.4607	0.0428417	No	Yes
21	AIRE	OpenTargets		0.0531636	No	No
22	ALB	GeneCards	2.40915		No	No
23	ALK3	OMIM			Yes	No
24	ALPI	GeneCards; OMIM	3.26586		Yes	Yes
25	ANK1	OpenTargets		0.0366993	No	No
26	ANO1	OMIM; OpenTargets		0.0305347	Yes	No

No.	Gene symbol	Source database(s)	GeneCards relevance score	Open Targets overall score	OMIM	In 102 shared targets
27	ANO5	OpenTargets		0.0348514	No	No
28	AP1M2	OpenTargets		0.0319908	No	No
29	APC	GeneCards	2.27473		No	No
30	APOE	OpenTargets		0.0387468	No	Yes
31	ARHGAP17	OpenTargets		0.031843	No	No
32	ARPC1B	OpenTargets		0.0448762	No	No
33	ASAH1	OpenTargets		0.0484501	No	No
34	ATG16L1	GeneCards	4.36215		No	No
35	ATG7	GeneCards	2.16988		No	No
36	ATP8B1	OpenTargets		0.0397261	No	No
37	B2M	GeneCards	2.94675		No	No
38	B3GNT6	OpenTargets		0.0387246	No	No
39	B4GALT1	OpenTargets		0.0340161	No	No
40	BACH2	GeneCards; OpenTargets	2.57044	0.0418106	No	No
41	BBS4	OpenTargets		0.0319687	No	No
42	BCL2	OpenTargets		0.0327374	No	Yes
43	BCL2L11	OpenTargets		0.0370689	No	No
44	BCL6	OpenTargets		0.0453364	No	No
45	BIN3	OpenTargets		0.0311038	No	No
46	BLOC1S6	OpenTargets		0.0333509	No	No
47	BMI1	OpenTargets		0.0347627	No	No
48	BMPR1A	OMIM			Yes	No
49	BPI	OpenTargets		0.0345114	No	No
50	BRCA1	OpenTargets		0.0326191	No	Yes
51	BTNL2	GeneCards	2.12611		No	No
52	C3	GeneCards	2.06518		No	No
53	C9orf72	OpenTargets		0.0465301	No	No
54	CACNB4	OpenTargets		0.0392863	No	Yes
55	CAID	OMIM			Yes	No
56	CAMSAP3	OpenTargets		0.0325526	No	No
57	CARD11	OpenTargets		0.0461533	No	No
58	CASP1	GeneCards	5.14464		No	No
59	CASP3	GeneCards	2.39548		No	Yes
60	CASP8	GeneCards	3.14471		No	Yes
61	CAV1	OpenTargets		0.0359749	No	Yes
62	CBY1	OpenTargets		0.0353171	No	No
63	CCL28	OpenTargets		0.0339422	No	No
64	CCN2	OpenTargets		0.0309043	No	No
65	CCR1	OpenTargets		0.0309486	No	No
66	CCR2	OpenTargets		0.0365958	No	No
67	CCR5	OpenTargets		0.0321978	No	No
68	CCR7	OpenTargets		0.034127	No	No
69	CD59	OpenTargets		0.0360119	No	No
70	CDKN1A	OpenTargets		0.0343488	No	Yes

No.	Gene symbol	Source database(s)	GeneCards relevance score	Open Targets overall score	OMIM	In 102 shared targets
71	CDX2	GeneCards	5.07006		No	No
72	CEBPE	OpenTargets		0.0514751	No	No
73	CERNA3	GeneCards	2.32579		No	No
74	CFTR	GeneCards; OpenTargets	4.27814	0.0545866	No	No
75	CGAS	GeneCards	2.0647		No	No
76	CHAK2	OMIM			Yes	No
77	CHD2	OpenTargets		0.0367806	No	No
78	CHGA	GeneCards	2.11689		No	No
79	CHI3L1	GeneCards	3.16186		No	No
80	CHRD	OpenTargets		0.0313625	No	No
81	CHRNA7	GeneCards	2.08414		No	Yes
82	CHST5	OMIM			Yes	No
83	CHSY1	OpenTargets		0.0328335	No	No
84	CHTOP	OpenTargets		0.0312517	No	No
85	CIAS1	OMIM			Yes	No
86	CISD2	OpenTargets		0.0307638	No	No
87	CISH	OpenTargets		0.0348292	No	No
88	CLDN1	GeneCards	3.53095		No	No
89	CLDN2	GeneCards	2.16399		No	Yes
90	CLEC4D	OpenTargets		0.0328187	No	No
91	CLK1	OpenTargets		0.0303942	No	Yes
92	CLOCK	OpenTargets		0.0345705	No	No
93	COG6	OpenTargets		0.0390498	No	No
94	COL1A1	OpenTargets		0.0304977	No	Yes
95	COMMD3-BMI1	OpenTargets		0.0347627	No	No
96	COPA	OpenTargets		0.0334618	No	No
97	COXFA4L3	OpenTargets		0.0325452	No	No
98	CRIP	OMIM			Yes	No
99	CRIP1	OMIM			Yes	No
100	CRIP2	OMIM			Yes	No
101	CRP	GeneCards	6.0703		No	Yes
102	CRP2	OMIM			Yes	No
103	CSBS	OMIM			Yes	No
104	CSF2RA	OpenTargets		0.0312517	No	No
105	CTLA4	GeneCards; OpenTargets	2.62346	0.0395007	No	No
106	CTNNB1	GeneCards	3.85309		No	Yes
107	CTSL	OpenTargets		0.0320426	No	No
108	CTSV	OpenTargets		0.0320426	No	No
109	CVD1	OMIM			Yes	No
110	CX3CR1	GeneCards	2.18639		No	No
111	CXCL8	GeneCards	3.57455		No	Yes
112	CXCR2	OpenTargets		0.03409	No	No
113	CYLD	GeneCards; OpenTargets	3.03922	0.0372684	No	No
114	CYP19A1	OpenTargets		0.0360784	No	Yes

No.	Gene symbol	Source database(s)	GeneCards relevance score	Open Targets overall score	OMIM	In 102 shared targets
115	DFNA34	OMIM			Yes	No
116	DKC1	OpenTargets		0.0372832	No	No
117	DLL1	OpenTargets		0.0448725	No	No
118	DLX5	OpenTargets		0.0311482	No	No
119	DNAJC3	OpenTargets		0.0306899	No	No
120	DPC4	OMIM			Yes	No
121	DPH6	OpenTargets		0.0307712	No	No
122	DPP4	OpenTargets		0.0322348	No	Yes
123	DSG3	OpenTargets		0.0409199	No	No
124	DTX1	OpenTargets		0.0335579	No	No
125	DUOXA2	OpenTargets		0.0391163	No	No
126	DUSP22	OpenTargets		0.0382811	No	No
127	E2F2	OpenTargets		0.0360045	No	Yes
128	E2F4	OpenTargets		0.04432	No	No
129	EDN2	OMIM			Yes	No
130	EFS	OpenTargets		0.0341787	No	No
131	EGFR	GeneCards; OpenTargets	2.15036	0.0393972	No	Yes
132	ELANE	GeneCards	2.60515		No	Yes
133	ENPP1	OpenTargets		0.0365367	No	Yes
134	ENPP3	OpenTargets		0.0366623	No	No
135	EPCAM	OpenTargets		0.0316804	No	No
136	ERCC2	OpenTargets		0.0309708	No	No
137	ERRFI1	OpenTargets		0.0320574	No	No
138	ESR1	OpenTargets		0.0312147	No	Yes
139	ESR2	OpenTargets		0.0365071	No	Yes
140	EYA1	OpenTargets		0.032013	No	No
141	EYA4	OpenTargets		0.0316434	No	No
142	FAA4	OMIM			Yes	No
143	FABP2	OMIM			Yes	Yes
144	FADS2	OpenTargets		0.0336835	No	No
145	FAM20C	OpenTargets		0.0334174	No	No
146	FAM3D	OpenTargets		0.0411342	No	No
147	FAS	OpenTargets		0.0435809	No	No
148	FASLG	OpenTargets		0.0464044	No	Yes
149	FBN1	OpenTargets		0.0469348	No	No
150	FBXL3	OpenTargets		0.03375	No	No
151	FBXO11	OpenTargets		0.0319908	No	No
152	FBXO7	OpenTargets		0.0335431	No	No
153	FBXW12	OpenTargets		0.0307786	No	No
154	FCAS1	OMIM			Yes	No
155	FCGR2A	OpenTargets		0.0396411	No	No
156	FCGR2B	OpenTargets		0.0396411	No	No
157	FCGR2C	OpenTargets		0.0396411	No	No
158	FCU	OMIM			Yes	No

No.	Gene symbol	Source database(s)	GeneCards relevance score	Open Targets overall score	OMIM	In 102 shared targets
159	FEM1A	OpenTargets		0.0309634	No	No
160	FEN1	OpenTargets		0.0402103	No	Yes
161	FFAR2	OpenTargets		0.047491	No	No
162	FGF20	OpenTargets		0.0349327	No	No
163	FGF23	OpenTargets		0.0324565	No	No
164	FGFR1	OpenTargets		0.0331587	No	Yes
165	FGFR2	OpenTargets		0.0426089	No	No
166	FGFRL1	OpenTargets		0.030823	No	No
167	FGL2	OpenTargets		0.0350657	No	No
168	FGS2	OMIM			Yes	No
169	FHIP2B	OpenTargets		0.0404246	No	No
170	FLJ10261	OMIM			Yes	No
171	FLN1	OMIM			Yes	No
172	FLNA	OMIM			Yes	No
173	FMD	OMIM			Yes	No
174	FNIP1	OpenTargets		0.0341566	No	No
175	FOXF1	OpenTargets		0.0306751	No	No
176	FOXO3	OpenTargets		0.0366401	No	No
177	FOXP3	GeneCards; OpenTargets	5.29189	0.0640103	No	Yes
178	FPR2	GeneCards; OpenTargets	2.379	0.0349696	No	No
179	FSHR	OpenTargets		0.0316508	No	No
180	FSTL1	OpenTargets		0.0313034	No	No
181	GAST	OpenTargets		0.0336392	No	No
182	GATM	OpenTargets		0.0317839	No	No
183	GBA1	OpenTargets		0.0397372	No	No
184	GCNT3	OpenTargets		0.0321756	No	No
185	GFUS	OpenTargets		0.0357458	No	No
186	GIMAP1-GIMAP5	OpenTargets		0.0531493	No	No
187	GIMAP5	OpenTargets		0.0531493	No	No
188	GKN2	OpenTargets		0.0354944	No	No
189	GMDS	OpenTargets		0.0356053	No	No
190	GNAI2	OpenTargets		0.0512977	No	No
191	GPA33	OpenTargets		0.0417699	No	No
192	GPR132	OpenTargets		0.0365958	No	No
193	GPR15	OpenTargets		0.0352579	No	No
194	GPX1	GeneCards	2.2841		No	No
195	GPX2	GeneCards	2.14394		No	No
196	GSDMD	GeneCards	2.1425		No	No
197	GUCA2	OMIM			Yes	No
198	GUCA2A	OMIM			Yes	Yes
199	GUCY2C	GeneCards	2.69529		No	No
200	GUCY2C-AS1	GeneCards	2.3999		No	No
201	Gene/Locus	OMIM			Yes	No
202	HCK	OpenTargets		0.037165	No	No

No.	Gene symbol	Source database(s)	GeneCards relevance score	Open Targets overall score	OMIM	In 102 shared targets
203	HIF1A	GeneCards	4.66719		No	Yes
204	HMGB1	GeneCards	2.02461		No	Yes
205	HMOX1	GeneCards; OpenTargets	4.82677	0.036108	No	Yes
206	HNF1A	OpenTargets		0.0420822	No	No
207	HOMG1	OMIM			Yes	No
208	HOXA3	OpenTargets		0.0340235	No	No
209	HOXA5	OpenTargets		0.0436474	No	No
210	HTR3A	GeneCards	2.08049		No	No
211	HYCC1	OpenTargets		0.0305716	No	No
212	IBSP	OpenTargets		0.0325156	No	No
213	IDO1	GeneCards	3.48127		No	Yes
214	IDS	OpenTargets		0.0330922	No	No
215	IFNG	GeneCards; OpenTargets	4.57811	0.0368323	No	Yes
216	IGF2BP1	OpenTargets		0.0309634	No	No
217	IKZF1	OpenTargets		0.0535166	No	No
218	IKZF3	OpenTargets		0.0336761	No	No
219	IL10	GeneCards; OpenTargets	5.17384	0.0517726	No	Yes
220	IL10RA	OpenTargets		0.0309782	No	No
221	IL10RB	OpenTargets		0.0381185	No	No
222	IL15	OpenTargets		0.0325378	No	No
223	IL17A	GeneCards; OpenTargets	2.77124	0.0333361	No	Yes
224	IL17B	OpenTargets		0.0317839	No	No
225	IL17RB	GeneCards	3.69766		No	No
226	IL17RE	OpenTargets		0.0329887	No	No
227	IL18	GeneCards	2.72898		No	No
228	IL1B	GeneCards	4.60399		No	Yes
229	IL1RN	GeneCards; OpenTargets	2.76952	0.0307121	No	No
230	IL2	OpenTargets		0.0598999	No	Yes
231	IL22	OpenTargets		0.0371871	No	No
232	IL23A	GeneCards	2.91096		No	No
233	IL25	OpenTargets		0.0365071	No	No
234	IL2RA	GeneCards; OpenTargets	2.47662	0.0529054	No	No
235	IL2RB	OpenTargets		0.0331883	No	No
236	IL2RG	OpenTargets		0.0535817	No	No
237	IL33	GeneCards	4.14724		No	Yes
238	IL4	OpenTargets		0.0342527	No	No
239	IL4R	OpenTargets		0.0328335	No	No
240	IL6	GeneCards; OpenTargets	4.23312	0.0353171	No	Yes
241	IL6ST	OpenTargets		0.0503423	No	No
242	IMD82	OMIM			Yes	No
243	INDMS	OMIM			Yes	No
244	INHA	OpenTargets		0.033787	No	No
245	INPP5D	OpenTargets		0.0422079	No	No
246	IRGM	GeneCards	3.15945		No	No

No.	Gene symbol	Source database(s)	GeneCards relevance score	Open Targets overall score	OMIM	In 102 shared targets
247	IRS1	OpenTargets		0.0309708	No	No
248	ITCH	OpenTargets		0.0348366	No	No
249	ITF	OMIM			Yes	No
250	ITGAV	GeneCards	2.22602		No	No
251	ITGB2	OpenTargets		0.0447764	No	No
252	ITGB3	OpenTargets		0.0363371	No	Yes
253	ITGB7	GeneCards	2.32939		No	No
254	ITPKB	OpenTargets		0.0384733	No	No
255	JAK1	OpenTargets		0.0409494	No	Yes
256	JAK2	GeneCards	2.36649		No	Yes
257	JAK3	OpenTargets		0.0433979	No	No
258	JAM3	OpenTargets		0.0322791	No	No
259	JIP	OMIM			Yes	No
260	KCNE2	OpenTargets		0.0409421	No	No
261	KCNN4	OpenTargets		0.0361227	No	No
262	KEFH	OMIM			Yes	No
263	KITLG	OpenTargets		0.0378154	No	No
264	KL	OpenTargets		0.0493609	No	No
265	KLRC1	OpenTargets		0.0318282	No	No
266	KLRC4	OpenTargets		0.0318282	No	No
267	KRT8	OpenTargets		0.0380298	No	No
268	LAMC2	OpenTargets		0.0308969	No	No
269	LAT	OpenTargets		0.0455396	No	No
270	LCN2	GeneCards	3.55974		No	No
271	LEP	OpenTargets		0.0316878	No	No
272	LEPR	OpenTargets		0.042838	No	No
273	LGALS3	GeneCards; OpenTargets	2.30267	0.0361745	No	No
274	LGALS4	GeneCards	3.57674		No	No
275	LIF	OpenTargets		0.0396559	No	No
276	LIG4	OpenTargets		0.0401807	No	No
277	LINC-ROR	GeneCards	2.07815		No	No
278	LINC01672	GeneCards	3.61113		No	No
279	LINC02605	GeneCards	5.54649		No	No
280	LIPA	OpenTargets		0.0387394	No	No
281	LMNA	OpenTargets		0.0365441	No	No
282	LMOD1	OMIM			Yes	No
283	LPO	OpenTargets		0.0423169	No	Yes
284	LRIG1	OpenTargets		0.0320943	No	No
285	LRRC19	GeneCards	2.0351		No	No
286	LRRK2	GeneCards	2.9521		No	No
287	LTA	OpenTargets		0.0328335	No	No
288	LTB	OpenTargets		0.0365367	No	No
289	LTBP4	OpenTargets		0.0410973	No	No
290	LTBR	GeneCards	2.70072		No	No

No.	Gene symbol	Source database(s)	GeneCards relevance score	Open Targets overall score	OMIM	In 102 shared targets
291	LTF	GeneCards	2.37345		No	Yes
292	LYN	OpenTargets		0.0451072	No	Yes
293	LYST	OpenTargets		0.0340457	No	No
294	MAD1L1	OMIM			Yes	No
295	MADH4	OMIM			Yes	No
296	MAN2A1	OpenTargets		0.0335431	No	No
297	MAP3K14	OpenTargets		0.0421025	No	No
298	MAPK1	GeneCards	2.48751		No	Yes
299	MAPK3	GeneCards	2.68671		No	Yes
300	MARK2	OpenTargets		0.0385915	No	No
301	MBD2	OpenTargets		0.0361671	No	No
302	MEFV	OpenTargets		0.0392124	No	No
303	MEP1B	GeneCards	4.4606		No	No
304	MGAT2	OpenTargets		0.0330035	No	No
305	MIR139	OpenTargets		0.0321239	No	No
306	MIR146A	OpenTargets		0.0341122	No	No
307	MIR146B	GeneCards	2.64469		No	No
308	MIR155	OpenTargets		0.0367584	No	No
309	MLC2	OMIM			Yes	No
310	MLCK	OMIM			Yes	No
311	MLXIPL	OpenTargets		0.0307343	No	Yes
312	MMIHS1	OMIM			Yes	No
313	MMIHS3	OMIM			Yes	No
314	MMIHS4	OMIM			Yes	No
315	MMIHS5	OMIM			Yes	No
316	MMP14	OpenTargets		0.0317839	No	Yes
317	MMP2	OpenTargets		0.0322939	No	Yes
318	MMP7	OpenTargets		0.0323013	No	No
319	MMP9	GeneCards; OpenTargets	2.5203	0.03171	No	Yes
320	MNS	OMIM			Yes	No
321	MPZL3	OpenTargets		0.0456856	No	No
322	MTOR	GeneCards	2.4454		No	Yes
323	MUC13	GeneCards; OpenTargets	2.14394	0.0383402	No	No
324	MUC2	GeneCards; OMIM; OpenTargets	4.54033	0.0527772	Yes	No
325	MUC3A	OMIM			Yes	No
326	MUC3B	OMIM			Yes	No
327	MVA7	OMIM			Yes	No
328	MYD88	OpenTargets		0.0476924	No	Yes
329	MYH11	OMIM			Yes	No
330	MYHRS	OMIM			Yes	No
331	MYL9	OMIM			Yes	No
332	MYLK	GeneCards; OMIM	4.20675		Yes	Yes
333	MYMY7	OMIM			Yes	No
334	MYO5B	OpenTargets		0.0303721	No	No

No.	Gene symbol	Source database(s)	GeneCards relevance score	Open Targets overall score	OMIM	In 102 shared targets
335	MYSM1	OpenTargets		0.0325156	No	No
336	N4BP1	OpenTargets		0.0401068	No	No
337	NALP3	OMIM			Yes	No
338	NCKAP1L	OpenTargets		0.0397446	No	No
339	NDFIP1	OpenTargets		0.0327374	No	No
340	NEDD4	OpenTargets		0.0336318	No	No
341	NFE2L2	OpenTargets		0.0368619	No	Yes
342	NFIL3	OpenTargets		0.0480084	No	No
343	NFKB1	GeneCards; OpenTargets	18.9167	0.0535254	No	Yes
344	NFKB2	OpenTargets		0.0518268	No	No
345	NFKBIA	GeneCards	2.49063		No	Yes
346	NFKBIZ	OpenTargets		0.036958	No	No
347	NHBP	OMIM			Yes	No
348	NKX2-1	OpenTargets		0.0335135	No	No
349	NKX2-3	OpenTargets		0.0445418	No	No
350	NLRP1	OpenTargets		0.0345631	No	No
351	NLRP12	OpenTargets		0.0305199	No	No
352	NLRP3	GeneCards; OMIM; OpenTargets	4.64225	0.0381776	Yes	Yes
353	NLRP6	GeneCards; OpenTargets	2.52088	0.0486626	No	No
354	NOD2	GeneCards; OpenTargets	6.23562	0.0337648	No	No
355	NOS2	GeneCards	4.77152		No	Yes
356	NOS3	OpenTargets		0.0420397	No	Yes
357	NPC1	OpenTargets		0.0308525	No	No
358	NR1H4	GeneCards; OpenTargets	6.2473	0.0359675	No	Yes
359	NR1I2	GeneCards	3.5635		No	Yes
360	NR5A2	GeneCards	2.45284		No	No
361	OCLN	OpenTargets		0.0447446	No	Yes
362	OLFM4	GeneCards; OpenTargets	2.20291	0.0316582	No	No
363	OPD1	OMIM			Yes	No
364	OPD2	OMIM			Yes	No
365	PAX8	OpenTargets		0.0313108	No	No
366	PCSK1	OpenTargets		0.037298	No	No
367	PDCD1	GeneCards	2.11546		No	No
368	PDCD6IP	OpenTargets		0.0346296	No	No
369	PGLYRP1	OpenTargets		0.03409	No	No
370	PGLYRP2	OpenTargets		0.03409	No	Yes
371	PGLYRP3	OpenTargets		0.03409	No	No
372	PGLYRP4	OpenTargets		0.03409	No	No
373	PGR-AS1	GeneCards	3.39047		No	No
374	PIEZO1	GeneCards	2.39892		No	No
375	PIK3C2A	OpenTargets		0.0333361	No	No
376	PIK3CD	OpenTargets		0.0519777	No	No
377	PIP	OpenTargets		0.0332622	No	No
378	PIP4K2C	OpenTargets		0.043433	No	No

No.	Gene symbol	Source database(s)	GeneCards relevance score	Open Targets overall score	OMIM	In 102 shared targets
379	PITPNA	OpenTargets		0.0392697	No	No
380	PITX3	OpenTargets		0.0353244	No	No
381	PLG	OpenTargets		0.0462622	No	Yes
382	PLPPR5	OpenTargets		0.03307	No	No
383	PLS1	OpenTargets		0.0339422	No	No
384	PPARG	GeneCards	5.76592		No	Yes
385	PRF1	OpenTargets		0.0398333	No	No
386	PRKAB1	OpenTargets		0.0342453	No	No
387	PRKCD	OpenTargets		0.0430265	No	Yes
388	PRKDC	OpenTargets		0.0547531	No	No
389	PSMB10	OpenTargets		0.0360858	No	No
390	PSPH	OpenTargets		0.0359971	No	Yes
391	PTGS1	GeneCards; OpenTargets	2.68829	0.0318578	No	No
392	PTGS2	GeneCards; OpenTargets	7.74303	0.0383106	No	Yes
393	PTPN2	GeneCards; OpenTargets	2.38499	0.0470623	No	Yes
394	PTPN22	OpenTargets		0.0377341	No	Yes
395	PTPN6	OpenTargets		0.0496615	No	No
396	PTPRC	OpenTargets		0.0332622	No	Yes
397	PYCARD	OpenTargets		0.0347035	No	No
398	PYPAF1	OMIM			Yes	No
399	RAG2	GeneCards; OpenTargets	2.30267	0.0524729	No	No
400	RASSF2	OpenTargets		0.0305864	No	No
401	RC3H1	OpenTargets		0.044867	No	No
402	REL	OpenTargets		0.0375715	No	No
403	RELA	GeneCards; OpenTargets	3.72695	0.0377637	No	Yes
404	RELB	GeneCards; OpenTargets	2.18032	0.0572824	No	No
405	RIGI	OpenTargets		0.036788	No	No
406	RIPK1	GeneCards	2.3299		No	No
407	RIPK3	GeneCards	2.78793		No	No
408	RNF128	OpenTargets		0.0332474	No	No
409	RNF20	OpenTargets		0.0361227	No	No
410	RNF43	OpenTargets		0.0306751	No	No
411	RORA	OpenTargets		0.031503	No	Yes
412	RORC	GeneCards; OpenTargets	5.16952	0.0368175	No	Yes
413	RPL38	OpenTargets		0.0327004	No	No
414	RRAGC	OpenTargets		0.0348366	No	No
415	RUNX3	OpenTargets		0.0544557	No	No
416	S100A12	GeneCards	3.36008		No	No
417	S100A8	GeneCards	2.28206		No	Yes
418	SALL4	OpenTargets		0.0321017	No	No
419	SAV1	OpenTargets		0.0344892	No	No
420	SCARF1	OpenTargets		0.0346075	No	No
421	SERPINB1	OpenTargets		0.0316656	No	No
422	SFN	OpenTargets		0.0339053	No	No

No.	Gene symbol	Source database(s)	GeneCards relevance score	Open Targets overall score	OMIM	In 102 shared targets
423	SFTPC	OpenTargets		0.0314586	No	No
424	SFTPD	OpenTargets		0.0359823	No	No
425	SGO	OMIM			Yes	No
426	SGO1	OMIM			Yes	No
427	SGOL1	OMIM			Yes	No
428	SH3BP2	OpenTargets		0.0469958	No	No
429	SHARPIN	OpenTargets		0.0485998	No	No
430	SHH	OpenTargets		0.0353023	No	No
431	SIK3	OpenTargets		0.0356053	No	No
432	SIRT1	GeneCards; OpenTargets	2.42954	0.0409033	No	Yes
433	SIRT6	OpenTargets		0.0371206	No	Yes
434	SIX1	OpenTargets		0.030993	No	No
435	SLC12A2	OpenTargets		0.0379411	No	No
436	SLC15A1	GeneCards	5.57895		No	No
437	SLC22A4	GeneCards	2.52232		No	No
438	SLC26A3	GeneCards; OpenTargets	2.55981	0.0307417	No	No
439	SLC29A3	OpenTargets		0.03375	No	No
440	SLC33A1	OpenTargets		0.0389094	No	No
441	SLC4A4	OpenTargets		0.0452865	No	No
442	SLC9A4	OpenTargets		0.0328409	No	No
443	SMAD3	OpenTargets		0.0474633	No	Yes
444	SMAD4	OMIM			Yes	No
445	SMAD5-AS1	GeneCards	2.06483		No	No
446	SMAD7	GeneCards	2.04664		No	No
447	SMCR8	OpenTargets		0.0429027	No	No
448	SMLMOD	OMIM			Yes	No
449	SNRNP40	OpenTargets		0.0367215	No	No
450	SNX10	OpenTargets		0.0476333	No	No
451	SOCS1	OpenTargets		0.039035	No	No
452	SOCS3	GeneCards	2.69903		No	No
453	SOX2	OpenTargets		0.031843	No	No
454	SOX21	OpenTargets		0.0312665	No	No
455	SPDEF	OpenTargets		0.0317543	No	No
456	SPRED1	OpenTargets		0.0359453	No	No
457	SPTA1	OpenTargets		0.0488455	No	No
458	SPTB	OpenTargets		0.0336244	No	No
459	STAT1	OpenTargets		0.0409199	No	Yes
460	STAT3	GeneCards	5.01204		No	Yes
461	STING1	GeneCards; OpenTargets	2.48062	0.0372536	No	No
462	SYK	OMIM; OpenTargets		0.633072	Yes	Yes
463	TANK	OpenTargets		0.0350953	No	No
464	TBX21	OpenTargets		0.0312369	No	No
465	TCF7	OpenTargets		0.0320278	No	No
466	TEN1	OpenTargets		0.036204	No	No

No.	Gene symbol	Source database(s)	GeneCards relevance score	Open Targets overall score	OMIM	In 102 shared targets
467	TEN1-CDK3	OpenTargets		0.036204	No	No
468	TENT5A	OpenTargets		0.0305569	No	No
469	TFF3	OMIM			Yes	No
470	TG	OpenTargets		0.0344596	No	No
471	TGFB1	GeneCards; OpenTargets	3.79714	0.059975	No	Yes
472	TGIF1	OpenTargets		0.0313552	No	No
473	THBS1	OpenTargets		0.0356275	No	Yes
474	THRA	OpenTargets		0.0424389	No	Yes
475	THUMPD3-AS1	OpenTargets		0.0342453	No	No
476	TIPE2	OpenTargets		0.0434552	No	No
477	TJP1	GeneCards	4.46104		No	Yes
478	TK1	OpenTargets		0.0312665	No	No
479	TLR2	GeneCards; OpenTargets	4.23757	0.036278	No	Yes
480	TLR4	GeneCards; OpenTargets	12.5152	0.0368619	No	Yes
481	TLR5	GeneCards; OpenTargets	5.71266	0.0476148	No	No
482	TLR7	OpenTargets		0.0504846	No	No
483	TLR9	GeneCards	3.34137		No	Yes
484	TMEM16A	OMIM			Yes	No
485	TNF	GeneCards; OpenTargets	7.71947	0.0510076	No	Yes
486	TNFAIP3	GeneCards; OpenTargets	2.78793	0.0395081	No	No
487	TNFAIP8	OpenTargets		0.0362632	No	No
488	TNFRSF11B	OpenTargets		0.031806	No	No
489	TNFRSF13B	OpenTargets		0.0344596	No	No
490	TNFRSF14	GeneCards	2.03237		No	No
491	TNFRSF1A	GeneCards; OpenTargets	2.10821	0.0335135	No	No
492	TNFRSF1B	OpenTargets		0.0325748	No	No
493	TNFRSF25	GeneCards	2.48062		No	No
494	TNFSF11	OpenTargets		0.0343266	No	Yes
495	TNFSF14	GeneCards	3.26048		No	No
496	TNFSF15	GeneCards	2.67884		No	No
497	TNIP1	OpenTargets		0.0448596	No	No
498	TOM1L2	OpenTargets		0.0371576	No	No
499	TOP3B	OpenTargets		0.0361597	No	No
500	TP53	OpenTargets		0.0448744	No	Yes
501	TRAF3IP2	OpenTargets		0.0371723	No	No
502	TREX1	OpenTargets		0.0529296	No	No
503	TRPM6	OMIM			Yes	No
504	TRPV5	OpenTargets		0.0310964	No	No
505	TTC7A	OpenTargets		0.0425571	No	No
506	TVP23B	OpenTargets		0.0329887	No	No
507	TVP23C	OpenTargets		0.0329887	No	No
508	TVP23C-CDRT4	OpenTargets		0.0329887	No	No
509	TWSG1	OpenTargets		0.0345631	No	No
510	TXBP181	OMIM			Yes	No

No.	Gene symbol	Source database(s)	GeneCards relevance score	Open Targets overall score	OMIM	In 102 shared targets
511	UACA	OpenTargets		0.0363371	No	No
512	ULK4	OpenTargets		0.0305347	No	No
513	UNC93B1	OpenTargets		0.0476111	No	No
514	UPK1A	OpenTargets		0.0330774	No	No
515	VDR	OpenTargets		0.0457836	No	Yes
516	VIP	OMIM			Yes	No
517	VIPR1	OMIM			Yes	No
518	VIPR2	OMIM			Yes	No
519	VSCM1	OMIM			Yes	No
520	VSCM2	OMIM			Yes	No
521	WAS	OpenTargets		0.0355536	No	No
522	WDR41	OpenTargets		0.0319095	No	No
523	WIPF1	OpenTargets		0.0445344	No	No
524	WWOX	OpenTargets		0.0422836	No	No
525	WWTR1	OpenTargets		0.0387079	No	No
526	XBP1	GeneCards	3.65036		No	Yes
527	XCL1	OpenTargets		0.0362262	No	No
528	XCL2	OpenTargets		0.0362262	No	No
529	YIPF6	OpenTargets		0.0395266	No	No
530	ZAP70	OpenTargets		0.0417625	No	No
531	ZDHHC13	OpenTargets		0.0355388	No	No
532	ZFP36	OpenTargets		0.0338387	No	No
533	ZSCAN10	OpenTargets		0.039789	No	No

Note. Blank score cells mean that the gene was not retained from that scored database source; they do not indicate a score of zero. OMIM records in the archived pipeline are represented as presence/absence because the supplied filtered OMIM file did not contain a comparable numerical association score.

Table S2C. Revision-stage human-to-feline orthology/mapping audit for the 102 shared targets carried forward to feline PPI analysis.

No.	Human/shared-target symbol	Revision-stage feline counterpart	Archived Felis catus STRING mapping	PPI status	Orthology/mapping audit
1	CASP3	CASP3	9685.ENSFCAP00000031946	Yes	Accepted for revision audit: same standardized symbol in the archived Felis catus STRING mapping.
2	TP53	TP53	9685.ENSFCAP00000045749	Yes	Accepted for revision audit: same standardized symbol in the archived Felis catus STRING mapping.
3	IL33	IL33	9685.ENSFCAP00000008387	Yes	Accepted for revision audit: same standardized symbol in the archived Felis catus STRING mapping.
4	NFKB1	NFKB1	9685.ENSFCAP00000006998	Yes	Accepted for revision audit: same standardized symbol in the archived Felis catus STRING mapping.
5	LPO	LPO	9685.ENSFCAP00000011377	Yes	Accepted for revision audit: same standardized symbol in the archived Felis catus STRING mapping.
6	DPP4	DPP4	9685.ENSFCAP00000037887	Yes	Accepted for revision audit: same standardized symbol in the archived Felis catus STRING mapping.
7	MMP9	MMP9	9685.ENSFCAP00000012997	Yes	Accepted for revision audit: same standardized symbol in the archived Felis catus STRING mapping.
8	MMP2	MMP2	9685.ENSFCAP00000040098	Yes	Accepted for revision audit: same standardized symbol in the archived Felis catus STRING mapping.
9	PRKCD	PRKCD	9685.ENSFCAP00000003598	Yes	Accepted for revision audit: same standardized symbol in the archived Felis catus STRING mapping.
10	ABCB1	ABCB1	9685.ENSFCAP00000034467	Yes	Accepted for revision audit: same standardized symbol in the archived Felis catus STRING mapping.
11	FASLG	FASLG	9685.ENSFCAP00000033003	Yes	Accepted for revision audit: same standardized symbol in the archived Felis catus STRING mapping.
12	MYD88	MYD88	9685.ENSFCAP00000036847	Yes	Accepted for revision audit: same standardized symbol in the archived Felis catus STRING

No.	Human/shared-target symbol	Revision-stage feline counterpart	Archived Felis catus STRING mapping	PPI status	Orthology/mapping audit
					mapping.
13	ACE2	ACE2	9685.ENSFCAP00000052000	Yes	Accepted for revision audit: same standardized symbol in the archived Felis catus STRING mapping.
14	NOS2	NOS2	9685.ENSFCAP00000037704	Yes	Accepted for revision audit: same standardized symbol in the archived Felis catus STRING mapping.
15	NFE2L2	NFE2L2	9685.ENSFCAP00000011270	Yes	Accepted for revision audit: same standardized symbol in the archived Felis catus STRING mapping.
16	NR1I2	NR1I2	9685.ENSFCAP00000005662	Yes	Accepted for revision audit: same standardized symbol in the archived Felis catus STRING mapping.
17	IL10	IL10	9685.ENSFCAP00000012535	Yes	Accepted for revision audit: same standardized symbol in the archived Felis catus STRING mapping.
18	TNF	TNF	9685.ENSFCAP00000051032	Yes	Accepted for revision audit: same standardized symbol in the archived Felis catus STRING mapping.
19	GUCA2A	GUCA2A	9685.ENSFCAP00000005747	No	Accepted for revision audit: same standardized symbol in the archived Felis catus STRING mapping.
20	TLR2	TLR2	9685.ENSFCAP00000020127	Yes	Accepted for revision audit: same standardized symbol in the archived Felis catus STRING mapping.
21	BCL2	BCL2	9685.ENSFCAP00000062072	Yes	Accepted for revision audit: same standardized symbol in the archived Felis catus STRING mapping.
22	TLR4	TLR4	9685.ENSFCAP00000023731	Yes	Accepted for revision audit: same standardized symbol in the archived Felis catus STRING mapping.
23	LTF	LTF	9685.ENSFCAP00000013454	Yes	Accepted for revision audit: same standardized symbol in the archived Felis catus STRING mapping.
24	PPARG	PPARG	9685.ENSFCAP00000050722	Yes	Accepted for revision audit: same standardized symbol in the archived Felis catus STRING mapping.
25	CDKN1A	CDKN1A	9685.ENSFCAP00000020450	Yes	Accepted for revision audit: same standardized symbol in the archived Felis catus STRING mapping.
26	NR1H4	NR1H4	9685.ENSFCAP00000006012	Yes	Accepted for revision audit: same standardized symbol in the archived Felis catus STRING mapping.
27	RORA	RORA	9685.ENSFCAP00000040308	Yes	Accepted for revision audit: same standardized symbol in the archived Felis catus STRING mapping.
28	CHRNA7	CHRNA7	9685.ENSFCAP00000047960	Yes	Accepted for revision audit: same standardized symbol in the archived Felis catus STRING mapping.
29	VDR	VDR	9685.ENSFCAP00000021493	Yes	Accepted for revision audit: same standardized symbol in the archived Felis catus STRING mapping.
30	FEN1	FEN1	9685.ENSFCAP00000012693	Yes	Accepted for revision audit: same standardized symbol in the archived Felis catus STRING mapping.
31	ESR1	ESR1	9685.ENSFCAP00000020448	Yes	Accepted for revision audit: same standardized symbol in the archived Felis catus STRING mapping.
32	PGLYRP2	PGLYRP2	9685.ENSFCAP00000012657	No	Accepted for revision audit: same standardized symbol in the archived Felis catus STRING mapping.
33	APOE	APOE	9685.ENSFCAP00000061228	No	Accepted for revision audit: same standardized symbol in the archived Felis catus STRING mapping.
34	C3	LOC111557813 (complement C3-like) NCBI Gene: 111557813	9685.ENSFCAP00000012390 preferred name: POLR3C	No (archived POLR3C mapping not retained)	FLAGGED. Archived STRING mapping is non-equivalent (C3→POLR3C). Current NCBI Felis catus record supports a complement C3-like counterpart, but the archived record must not be described as a C3 ortholog.
35	S100A8	S100A8	9685.ENSFCAP00000001199	Yes	Accepted for revision audit: same standardized symbol in the archived Felis catus STRING mapping.
36	PSPH	PSPH	9685.ENSFCAP00000017540	No	Accepted for revision audit: same standardized symbol in the archived Felis catus STRING mapping.
37	LYN	LYN	9685.ENSFCAP00000022129	Yes	Accepted for revision audit: same standardized symbol in the archived Felis catus STRING mapping.
38	HMOX1	HMOX1	9685.ENSFCAP00000062192	Yes	Accepted for revision audit: same standardized symbol in the archived Felis catus STRING mapping.
39	PTGS2	PTGS2	9685.ENSFCAP00000020047	Yes	Accepted for revision audit: same standardized symbol in the archived Felis catus STRING mapping.
40	CLDN2	CLDN2	9685.ENSFCAP00000013583	Yes	Accepted for revision audit: same standardized symbol in the archived Felis catus STRING mapping.
41	CXCL8	CXCL8	9685.ENSFCAP00000004310	Yes	Accepted for revision audit: same standardized symbol in the archived Felis catus STRING mapping.

No.	Human/shared-target symbol	Revision-stage feline counterpart	Archived Felis catus STRING mapping	PPI status	Orthology/mapping audit
42	STAT1	STAT1	9685.ENSFCAP00000050611	Yes	Accepted for revision audit: same standardized symbol in the archived Felis catus STRING mapping.
43	TJP1	TJP1	9685.ENSFCAP00000027327	Yes	Accepted for revision audit: same standardized symbol in the archived Felis catus STRING mapping.
44	STAT3	STAT3	9685.ENSFCAP00000001991	Yes	Accepted for revision audit: same standardized symbol in the archived Felis catus STRING mapping.
45	SYK	SYK	9685.ENSFCAP00000013189	Yes	Accepted for revision audit: same standardized symbol in the archived Felis catus STRING mapping.
46	FOXP3	FOXP3	9685.ENSFCAP00000043740	Yes	Accepted for revision audit: same standardized symbol in the archived Felis catus STRING mapping.
47	NFKBIA	NFKBIA	9685.ENSFCAP00000006200	Yes	Accepted for revision audit: same standardized symbol in the archived Felis catus STRING mapping.
48	AHR	AHR	9685.ENSFCAP00000004645	Yes	Accepted for revision audit: same standardized symbol in the archived Felis catus STRING mapping.
49	ITGB3	ITGB3	9685.ENSFCAP00000033446	Yes	Accepted for revision audit: same standardized symbol in the archived Felis catus STRING mapping.
50	CTNNB1	CTNNB1	9685.ENSFCAP00000003200	Yes	Accepted for revision audit: same standardized symbol in the archived Felis catus STRING mapping.
51	HIF1A	HIF1A	9685.ENSFCAP00000003163	Yes	Accepted for revision audit: same standardized symbol in the archived Felis catus STRING mapping.
52	MTOR	MTOR	9685.ENSFCAP00000000048	Yes	Accepted for revision audit: same standardized symbol in the archived Felis catus STRING mapping.
53	IL1B	IL1B	9685.ENSFCAP00000005482	Yes	Accepted for revision audit: same standardized symbol in the archived Felis catus STRING mapping.
54	SIRT1	SIRT1	9685.ENSFCAP00000027641	Yes	Accepted for revision audit: same standardized symbol in the archived Felis catus STRING mapping.
55	SMAD3	SMAD3	9685.ENSFCAP00000034334	Yes	Accepted for revision audit: same standardized symbol in the archived Felis catus STRING mapping.
56	JAK1	JAK1	9685.ENSFCAP00000001806	Yes	Accepted for revision audit: same standardized symbol in the archived Felis catus STRING mapping.
57	MAPK1	MAPK1	9685.ENSFCAP00000025541	Yes	Accepted for revision audit: same standardized symbol in the archived Felis catus STRING mapping.
58	TNFSF11	TNFSF11	9685.ENSFCAP00000005491	Yes	Accepted for revision audit: same standardized symbol in the archived Felis catus STRING mapping.
59	JAK2	JAK2	9685.ENSFCAP00000049673	Yes	Accepted for revision audit: same standardized symbol in the archived Felis catus STRING mapping.
60	TGFB1	TGFB1	9685.ENSFCAP00000005040	Yes	Accepted for revision audit: same standardized symbol in the archived Felis catus STRING mapping.
61	MAPK3	MAPK3	9685.ENSFCAP00000034399	Yes	Accepted for revision audit: same standardized symbol in the archived Felis catus STRING mapping.
62	OCLN	OCLN	9685.ENSFCAP00000007138	Yes	Accepted for revision audit: same standardized symbol in the archived Felis catus STRING mapping.
63	CYP19A1	CYP19A1	9685.ENSFCAP00000018380	Yes	Accepted for revision audit: same standardized symbol in the archived Felis catus STRING mapping.
64	ESR2	ESR2	9685.ENSFCAP00000042538	Yes	Accepted for revision audit: same standardized symbol in the archived Felis catus STRING mapping.
65	IL2	IL2	9685.ENSFCAP00000012696	Yes	Accepted for revision audit: same standardized symbol in the archived Felis catus STRING mapping.
66	E2F2	E2F2	9685.ENSFCAP00000010812	Yes	Accepted for revision audit: same standardized symbol in the archived Felis catus STRING mapping.
67	THBS1	THBS1	9685.ENSFCAP00000010636	Yes	Accepted for revision audit: same standardized symbol in the archived Felis catus STRING mapping.
68	CASP8	CASP8	9685.ENSFCAP00000055230	Yes	Accepted for revision audit: same standardized symbol in the archived Felis catus STRING mapping.
69	CRP	CRP	9685.ENSFCAP00000058298	Yes	Accepted for revision audit: same standardized symbol in the archived Felis catus STRING mapping.
70	RELA	RELA	9685.ENSFCAP00000048226	Yes	Accepted for revision audit: same standardized symbol in the archived Felis catus STRING mapping.
71	COL1A1	COL1A1	9685.ENSFCAP00000004218	Yes	Accepted for revision audit: same standardized symbol in the archived Felis catus STRING mapping.

No.	Human/shared-target symbol	Revision-stage feline counterpart	Archived Felis catus STRING mapping	PPI status	Orthology/mapping audit
72	PTGS1	PTGS1<br>Official feline symbol: VGNC:69147	9685.ENSFCAP00000056601<br>preferred name: MRRF	FLAGGED: MRRF retained in archived PPI	FLAGGED. Archived STRING mapping is non-equivalent (PTGS1→MRRF). Feline PTGS1 is independently supported by VGNC and feline genomic literature; a corrected species-specific identifier is required for PPI re-analysis.
73	IL6	IL6	9685.ENSFCAP00000022423	Yes	Accepted for revision audit: same standardized symbol in the archived Felis catus STRING mapping.
74	XBP1	XBP1	9685.ENSFCAP00000041558	Yes	Accepted for revision audit: same standardized symbol in the archived Felis catus STRING mapping.
75	IL17A	IL17A	9685.ENSFCAP00000033272	Yes	Accepted for revision audit: same standardized symbol in the archived Felis catus STRING mapping.
76	CAV1	CAV1	9685.ENSFCAP00000029296	Yes	Accepted for revision audit: same standardized symbol in the archived Felis catus STRING mapping.
77	BRCA1	BRCA1	9685.ENSFCAP00000012193	Yes	Accepted for revision audit: same standardized symbol in the archived Felis catus STRING mapping.
78	MLXIPL	MLXIPL	9685.ENSFCAP00000004390	Yes	Accepted for revision audit: same standardized symbol in the archived Felis catus STRING mapping.
79	NOS3	NOS3	9685.ENSFCAP00000012234	Yes	Accepted for revision audit: same standardized symbol in the archived Felis catus STRING mapping.
80	EGFR	EGFR	9685.ENSFCAP00000041464	Yes	Accepted for revision audit: same standardized symbol in the archived Felis catus STRING mapping.
81	NLRP3	NLRP3	9685.ENSFCAP00000020543	Yes	Accepted for revision audit: same standardized symbol in the archived Felis catus STRING mapping.
82	IFNG	IFNG	9685.ENSFCAP00000008355	Yes	Accepted for revision audit: same standardized symbol in the archived Felis catus STRING mapping.
83	ABCA1	ABCA1	9685.ENSFCAP00000004021	Yes	Accepted for revision audit: same standardized symbol in the archived Felis catus STRING mapping.
84	CACNB4	CACNB4	9685.ENSFCAP00000033196	No	Accepted for revision audit: same standardized symbol in the archived Felis catus STRING mapping.
85	ALPI	ALPI	9685.ENSFCAP00000050011	No	Accepted for revision audit: same standardized symbol in the archived Felis catus STRING mapping.
86	THRA	THRA	9685.ENSFCAP00000053212	No	Accepted for revision audit: same standardized symbol in the archived Felis catus STRING mapping.
87	MYLK	MYLK	9685.ENSFCAP00000043407	No	Accepted for revision audit: same standardized symbol in the archived Felis catus STRING mapping.
88	ELANE	ELANE	9685.ENSFCAP00000057052	Yes	Accepted for revision audit: same standardized symbol in the archived Felis catus STRING mapping.
89	PTPRC	PTPRC	9685.ENSFCAP00000056536	Yes	Accepted for revision audit: same standardized symbol in the archived Felis catus STRING mapping.
90	TLR9	TLR9	9685.ENSFCAP00000038661	Yes	Accepted for revision audit: same standardized symbol in the archived Felis catus STRING mapping.
91	CLK1	CLK1	9685.ENSFCAP00000050399	No	Accepted for revision audit: same standardized symbol in the archived Felis catus STRING mapping.
92	PTPN22	PTPN22	9685.ENSFCAP00000061870	Yes	Accepted for revision audit: same standardized symbol in the archived Felis catus STRING mapping.
93	IDO1	IDO1	9685.ENSFCAP00000003351	Yes	Accepted for revision audit: same standardized symbol in the archived Felis catus STRING mapping.
94	PTPN2	PTPN2	9685.ENSFCAP00000043457	Yes	Accepted for revision audit: same standardized symbol in the archived Felis catus STRING mapping.
95	FGFR1	FGFR1	9685.ENSFCAP00000032682	Yes	Accepted for revision audit: same standardized symbol in the archived Felis catus STRING mapping.
96	SIRT6	SIRT6	9685.ENSFCAP00000050094	Yes	Accepted for revision audit: same standardized symbol in the archived Felis catus STRING mapping.
97	MMP14	MMP14	9685.ENSFCAP00000006156	Yes	Accepted for revision audit: same standardized symbol in the archived Felis catus STRING mapping.
98	FABP2	FABP2	9685.ENSFCAP00000016766	Yes	Accepted for revision audit: same standardized symbol in the archived Felis catus STRING mapping.
99	RORC	RORC	9685.ENSFCAP00000056184	Yes	Accepted for revision audit: same standardized symbol in the archived Felis catus STRING mapping.
100	PLG	PLG	9685.ENSFCAP00000021289	Yes	Accepted for revision audit: same standardized symbol in the archived Felis catus STRING mapping.
101	ENPP1	ENPP1	9685.ENSFCAP00000060910	Yes	Accepted for revision audit: same standardized symbol in the archived Felis catus STRING mapping.

No.	Human/shared-target symbol	Revision-stage feline counterpart	Archived Felis catus STRING mapping	PPI status	Orthology/mapping audit
102	HMGB1	HMGB1	9685.ENSFCAP00000058007	Yes	Accepted for revision audit: same standardized symbol in the archived Felis catus STRING mapping.

Revision-stage mapping interpretation: the archived Felis catus STRING identifier-mapping output returned a species-specific record for all 102 shared-target queries. However, exact preferred-name checking showed that 100/102 queries resolved to the same standardized gene symbol, whereas two queries were non-equivalent mappings: C3→POLR3C and PTGS1→MRRF. These two records are therefore flagged rather than treated as feline orthologs. Current external verification identifies a feline complement C3-like gene record (NCBI Gene 111557813) and an official feline PTGS1 symbol (VGNC:69147). Accordingly, the table below is a revision-stage cross-species audit, not a reconstruction of an unarchived historical biomaRt output.
Orthology and downstream-PPI limitation: the supplied analysis archive does not preserve a row-level biomaRt human→Felis catus ortholog table for the complete 533-gene disease set. NCBI describes vertebrate ortholog groups as being inferred using protein-sequence similarity together with local synteny, and such a formal ortholog set should be used for any full re-mapping. For the present revision audit, only the 102 shared targets actually propagated to the feline PPI/enrichment workflow were examined. The archived PTGS1→MRRF mismatch is particularly important because MRRF is present in the archived 92-node PPI network; therefore, a corrected feline PPI re-run is recommended before the manuscript is finalized. The C3→POLR3C mismatch was not retained in the 92-node PPI network. These findings do not by themselves establish functional or pharmacological equivalence between human and feline genes.

Archived enrichment summary: 195 GO terms were retained in the archived output (171 BP, 24 MF, 0 CC). All 195 GO rows have nominal $P < 0.05$, whereas 107 have adjusted $P < 0.05$. The archived KEGG output contains 173 pathways; 150 have nominal $P < 0.05$ and 141 have adjusted $P < 0.05$. The complete rows are reported below irrespective of whether the adjusted P value is below 0.05.

Revision-stage orthology source note: NCBI Gene FTP/Datasets documentation (accessed 25 August 2026) states that vertebrate ortholog groups are calculated using protein-sequence similarity and local synteny; current NCBI gene records and VGNC nomenclature were used to audit the two non-equivalent STRING mappings identified above. The original 102-query STRING mapping file is retained as the archived downstream-analysis record.

Table S2D. Complete GO enrichment results: ontology, term identity, gene ratios, counts, and significance statistics.

No.	Ontology	GO ID	Description	GeneRatio	BgRatio	Count	P value	Adjusted P	q value
1	BP	GO:0006952	defense response	24/94	396/14350	24	4.752E-17	1.546E-13	1.546E-13
2	BP	GO:0009605	response to external stimulus	21/94	442/14350	21	6.800E-13	1.106E-09	1.106E-09
3	BP	GO:0006954	inflammatory response	14/94	172/14350	14	5.623E-12	6.099E-09	6.099E-09
4	BP	GO:0043207	response to external biotic stimulus	16/94	289/14350	16	5.417E-11	3.091E-08	3.091E-08
5	BP	GO:0051707	response to other organism	16/94	289/14350	16	5.417E-11	3.091E-08	3.091E-08
6	BP	GO:0009607	response to biotic stimulus	16/94	291/14350	16	6.004E-11	3.091E-08	3.091E-08
7	BP	GO:0044419	biological process involved in interspecies interaction between organisms	16/94	293/14350	16	6.650E-11	3.091E-08	3.091E-08
8	BP	GO:0140546	defense response to symbiont	13/94	217/14350	13	1.546E-09	6.052E-07	6.052E-07
9	BP	GO:0098542	defense response to other organism	14/94	265/14350	14	1.777E-09	6.052E-07	6.052E-07
10	BP	GO:0031347	regulation of defense response	10/94	107/14350	10	1.860E-09	6.052E-07	6.052E-07
11	BP	GO:0045087	innate immune response	11/94	184/14350	11	3.129E-08	9.257E-06	9.257E-06
12	BP	GO:0032101	regulation of response to external stimulus	10/94	157/14350	10	7.580E-08	2.056E-05	2.056E-05
13	BP	GO:0080134	regulation of response to stress	10/94	171/14350	10	1.693E-07	4.238E-05	4.238E-05
14	BP	GO:0042127	regulation of cell population proliferation	10/94	181/14350	10	2.876E-07	6.685E-05	6.685E-05
15	BP	GO:0008283	cell population proliferation	10/94	210/14350	10	1.128E-06	2.447E-04	2.447E-04
16	BP	GO:0002221	pattern recognition receptor signaling pathway	5/94	30/14350	5	1.356E-06	2.758E-04	2.758E-04
17	BP	GO:0034097	response to cytokine	9/94	174/14350	9	2.025E-06	3.660E-04	3.660E-04
18	BP	GO:1901652	response to peptide	9/94	174/14350	9	2.025E-06	3.660E-04	3.660E-04
19	BP	GO:0038061	non-canonical NF-kappaB signal transduction	4/94	15/14350	4	2.230E-06	3.819E-04	3.819E-04
20	BP	GO:0031349	positive regulation of defense response	6/94	60/14350	6	2.532E-06	4.120E-04	4.120E-04
21	BP	GO:0009617	response to bacterium	7/94	101/14350	7	4.303E-06	6.231E-04	6.231E-04
22	BP	GO:0030097	hemopoiesis	7/94	101/14350	7	4.303E-06	6.231E-04	6.231E-04
23	BP	GO:0002758	innate immune response-activating signaling pathway	5/94	38/14350	5	4.584E-06	6.231E-04	6.231E-04
24	BP	GO:0002757	immune response-activating signaling pathway	7/94	102/14350	7	4.595E-06	6.231E-04	6.231E-04
25	BP	GO:0002764	immune response-regulating signaling pathway	7/94	106/14350	7	5.937E-06	7.728E-04	7.728E-04
26	BP	GO:0002218	activation of innate immune response	5/94	42/14350	5	7.609E-06	9.303E-04	9.303E-04
27	BP	GO:0050728	negative regulation of inflammatory response	4/94	20/14350	4	7.719E-06	9.303E-04	9.303E-04
28	BP	GO:0050727	regulation of inflammatory response	5/94	45/14350	5	1.076E-05	0.00120857	0.00120857
29	BP	GO:0010557	positive regulation of macromolecule biosynthetic process	12/94	398/14350	12	1.077E-05	0.00120857	0.00120857
30	BP	GO:0002224	toll-like receptor signaling pathway	4/94	22/14350	4	1.154E-05	0.00125154	0.00125154
31	BP	GO:0009891	positive regulation of biosynthetic process	12/94	406/14350	12	1.315E-05	0.00138	0.00138
32	BP	GO:0031348	negative regulation of defense response	4/94	23/14350	4	1.390E-05	0.00141322	0.00141322
33	BP	GO:0002253	activation of immune response	7/94	125/14350	7	1.756E-05	0.0017319	0.0017319
34	BP	GO:0032103	positive regulation of response to external stimulus	6/94	84/14350	6	1.812E-05	0.00173442	0.00173442
35	BP	GO:0045089	positive regulation of innate immune response	5/94	53/14350	5	2.425E-05	0.00225468	0.00225468
36	BP	GO:0002833	positive regulation of response to biotic stimulus	5/94	57/14350	5	3.466E-05	0.00313305	0.00313305

No.	Ontology	GO ID	Description	GeneRatio	BgRatio	Count	P value	Adjusted P	q value
37	BP	GO:0045944	positive regulation of transcription by R polymerase II	8/94	191/14350	8	3.564E-05	0.00313436	0.00313436
38	BP	GO:0045088	regulation of innate immune response	5/94	58/14350	5	3.774E-05	0.00323145	0.00323145
39	BP	GO:0008284	positive regulation of cell population proliferation	6/94	104/14350	6	6.091E-05	0.0050204	0.0050204
40	BP	GO:0019221	cytokine-mediated signaling pathway	7/94	152/14350	7	6.171E-05	0.0050204	0.0050204
41	BP	GO:0002831	regulation of response to biotic stimulus	5/94	65/14350	5	6.561E-05	0.00520751	0.00520751
42	BP	GO:0070887	cellular response to chemical stimulus	10/94	341/14350	10	7.752E-05	0.00600573	0.00600573
43	BP	GO:0071345	cellular response to cytokine stimulus	7/94	159/14350	7	8.201E-05	0.00613231	0.00613231
44	BP	GO:0009719	response to endogenous stimulus	9/94	277/14350	9	8.292E-05	0.00613231	0.00613231
45	BP	GO:0008625	extrinsic apoptotic signaling pathway via death domain receptors	3/94	14/14350	3	9.403E-05	0.00665189	0.00665189
46	BP	GO:0046631	alpha-beta T cell activation	3/94	14/14350	3	9.403E-05	0.00665189	0.00665189
47	BP	GO:0006915	apoptotic process	9/94	288/14350	9	1.116E-04	0.00772388	0.00772388
48	BP	GO:0009755	hormone-mediated signaling pathway	4/94	39/14350	4	1.191E-04	0.00807708	0.00807708
49	BP	GO:0012501	programmed cell death	9/94	294/14350	9	1.304E-04	0.00866007	0.00866007
50	BP	GO:0008219	cell death	9/94	295/14350	9	1.338E-04	0.00870723	0.00870723
51	BP	GO:0050778	positive regulation of immune response	7/94	173/14350	7	1.390E-04	0.00886807	0.00886807
52	BP	GO:0042102	positive regulation of T cell proliferation	3/94	16/14350	3	1.433E-04	0.0089672	0.0089672
53	BP	GO:0007249	canonical NF-kappaB signal transduction	5/94	77/14350	5	1.475E-04	0.00905852	0.00905852
54	BP	GO:0009725	response to hormone	6/94	123/14350	6	1.544E-04	0.00927912	0.00927912
55	BP	GO:0033993	response to lipid	5/94	78/14350	5	1.568E-04	0.00927912	0.00927912
56	BP	GO:0097191	extrinsic apoptotic signaling pathway	4/94	42/14350	4	1.597E-04	0.00928099	0.00928099
57	BP	GO:0007259	cell surface receptor signaling pathway via JAK-STAT	4/94	44/14350	4	1.918E-04	0.0105775	0.0105775
58	BP	GO:0018108	peptidyl-tyrosine phosphorylation	4/94	44/14350	4	1.918E-04	0.0105775	0.0105775
59	BP	GO:0097696	cell surface receptor signaling pathway via STAT	4/94	44/14350	4	1.918E-04	0.0105775	0.0105775
60	BP	GO:0002684	positive regulation of immune system process	8/94	247/14350	8	2.140E-04	0.0116053	0.0116053
61	BP	GO:0018212	peptidyl-tyrosine modification	4/94	46/14350	4	2.283E-04	0.0121759	0.0121759
62	BP	GO:0032946	positive regulation of mononuclear cell proliferation	3/94	20/14350	3	2.862E-04	0.0145191	0.0145191
63	BP	GO:0050671	positive regulation of lymphocyte proliferation	3/94	20/14350	3	2.862E-04	0.0145191	0.0145191
64	BP	GO:0070665	positive regulation of leukocyte proliferation	3/94	20/14350	3	2.862E-04	0.0145191	0.0145191
65	BP	GO:0050776	regulation of immune response	7/94	195/14350	7	2.900E-04	0.0145191	0.0145191
66	BP	GO:0032102	negative regulation of response to external stimulus	4/94	50/14350	4	3.158E-04	0.0155675	0.0155675
67	BP	GO:0071495	cellular response to endogenous stimulus	8/94	263/14350	8	3.270E-04	0.01588	0.01588
68	BP	GO:0030098	lymphocyte differentiation	4/94	51/14350	4	3.409E-04	0.0163139	0.0163139
69	BP	GO:0006809	nitric oxide biosynthetic process	2/94	5/14350	2	4.191E-04	0.0189428	0.0189428
70	BP	GO:0046209	nitric oxide metabolic process	2/94	5/14350	2	4.191E-04	0.0189428	0.0189428
71	BP	GO:0046633	alpha-beta T cell proliferation	2/94	5/14350	2	4.191E-04	0.0189428	0.0189428
72	BP	GO:0046640	regulation of alpha-beta T cell proliferation	2/94	5/14350	2	4.191E-04	0.0189428	0.0189428
73	BP	GO:0030217	T cell differentiation	3/94	23/14350	3	4.384E-04	0.0192763	0.0192763
74	BP	GO:0030574	collagen catabolic process	3/94	23/14350	3	4.384E-04	0.0192763	0.0192763
75	BP	GO:0006979	response to oxidative stress	4/94	55/14350	4	4.561E-04	0.0195271	0.0195271
76	BP	GO:1903131	mononuclear cell differentiation	4/94	55/14350	4	4.561E-04	0.0195271	0.0195271
77	BP	GO:0045893	positive regulation of D-templated transcription	8/94	278/14350	8	4.732E-04	0.0197427	0.0197427
78	BP	GO:1902680	positive regulation of R biosynthetic process	8/94	278/14350	8	4.732E-04	0.0197427	0.0197427
79	BP	GO:0042742	defense response to bacterium	4/94	56/14350	4	4.887E-04	0.0201299	0.0201299
80	BP	GO:0030522	intracellular receptor signaling pathway	3/94	25/14350	3	5.639E-04	0.0229381	0.0229381
81	BP	GO:0032963	collagen metabolic process	3/94	26/14350	3	6.345E-04	0.0254887	0.0254887
82	BP	GO:0032870	cellular response to hormone stimulus	5/94	109/14350	5	7.401E-04	0.0293701	0.0293701
83	BP	GO:0042129	regulation of T cell proliferation	3/94	28/14350	3	7.919E-04	0.0310463	0.0310463
84	BP	GO:0071396	cellular response to lipid	4/94	65/14350	4	8.613E-04	0.0328804	0.0328804
85	BP	GO:0002526	acute inflammatory response	2/94	7/14350	2	8.727E-04	0.0328804	0.0328804
86	BP	GO:2001057	reactive nitrogen species metabolic process	2/94	7/14350	2	8.727E-04	0.0328804	0.0328804

No.	Ontology	GO ID	Description	GeneRatio	BgRatio	Count	P value	Adjusted P	q value
87	BP	GO:0042098	T cell proliferation	3/94	29/14350	3	8.791E-04	0.0328804	0.0328804
88	BP	GO:0002521	leukocyte differentiation	4/94	68/14350	4	0.00102069	0.0377423	0.0377423
89	BP	GO:0051254	positive regulation of R metabolic process	8/94	318/14350	8	0.00113588	0.0415298	0.0415298
90	BP	GO:1901222	regulation of non-canonical NF-kappaB signal transduction	2/94	8/14350	2	0.00115863	0.041891	0.041891
91	BP	GO:0007159	leukocyte cell-cell adhesion	4/94	72/14350	4	0.00126399	0.0451979	0.0451979
92	BP	GO:0032944	regulation of mononuclear cell proliferation	3/94	34/14350	3	0.001406	0.0486715	0.0486715
93	BP	GO:0050670	regulation of lymphocyte proliferation	3/94	34/14350	3	0.001406	0.0486715	0.0486715
94	BP	GO:0070663	regulation of leukocyte proliferation	3/94	34/14350	3	0.001406	0.0486715	0.0486715
95	BP	GO:0046634	regulation of alpha-beta T cell activation	2/94	9/14350	2	0.00148332	0.0508077	0.0508077
96	BP	GO:1901700	response to oxygen-containing compound	6/94	191/14350	6	0.00158672	0.0537831	0.0537831
97	BP	GO:0002682	regulation of immune system process	8/94	342/14350	8	0.00180078	0.0604097	0.0604097
98	BP	GO:0051341	regulation of oxidoreductase activity	2/94	10/14350	2	0.00184625	0.0613032	0.0613032
99	BP	GO:0043066	negative regulation of apoptotic process	4/94	82/14350	4	0.00204469	0.0672062	0.0672062
100	BP	GO:0043069	negative regulation of programmed cell death	4/94	83/14350	4	0.00213757	0.0695566	0.0695566
101	BP	GO:0043401	steroid hormone receptor signaling pathway	2/94	11/14350	2	0.00224692	0.072391	0.072391
102	BP	GO:0042981	regulation of apoptotic process	6/94	206/14350	6	0.0023233	0.0741177	0.0741177
103	BP	GO:0043067	regulation of programmed cell death	6/94	209/14350	6	0.00249724	0.0788934	0.0788934
104	BP	GO:0032496	response to lipopolysaccharide	3/94	42/14350	3	0.0025973	0.0812656	0.0812656
105	BP	GO:0048584	positive regulation of response to stimulus	9/94	449/14350	9	0.00268739	0.0832835	0.0832835
106	BP	GO:0097190	apoptotic signaling pathway	4/94	94/14350	4	0.00335884	0.102493	0.102493
107	BP	GO:0002237	response to molecule of bacterial origin	3/94	46/14350	3	0.00337024	0.102493	0.102493
108	BP	GO:0050870	positive regulation of T cell activation	3/94	47/14350	3	0.0035831	0.106442	0.106442
109	BP	GO:1903039	positive regulation of leukocyte cell-cell adhesion	3/94	47/14350	3	0.0035831	0.106442	0.106442
110	BP	GO:0048585	negative regulation of response to stimulus	7/94	301/14350	7	0.00359823	0.106442	0.106442
111	BP	GO:0042110	T cell activation	4/94	101/14350	4	0.00434383	0.127341	0.127341
112	BP	GO:0007155	cell adhesion	9/94	486/14350	9	0.00452704	0.131527	0.131527
113	BP	GO:0022409	positive regulation of cell-cell adhesion	3/94	52/14350	3	0.0047699	0.136983	0.136983
114	BP	GO:0050678	regulation of epithelial cell proliferation	2/94	16/14350	2	0.00479904	0.136983	0.136983
115	BP	GO:0032943	mononuclear cell proliferation	3/94	53/14350	3	0.00503237	0.138774	0.138774
116	BP	GO:0046651	lymphocyte proliferation	3/94	53/14350	3	0.00503237	0.138774	0.138774
117	BP	GO:0050851	antigen receptor-mediated signaling pathway	3/94	53/14350	3	0.00503237	0.138774	0.138774
118	BP	GO:0070661	leukocyte proliferation	3/94	53/14350	3	0.00503237	0.138774	0.138774
119	BP	GO:0050673	epithelial cell proliferation	2/94	17/14350	2	0.00541583	0.148093	0.148093
120	BP	GO:0050830	defense response to Gram-positive bacterium	2/94	18/14350	2	0.00606695	0.161818	0.161818
121	BP	GO:0071383	cellular response to steroid hormone stimulus	2/94	18/14350	2	0.00606695	0.161818	0.161818
122	BP	GO:0141193	nuclear receptor-mediated signaling pathway	2/94	18/14350	2	0.00606695	0.161818	0.161818
123	BP	GO:0007167	enzyme-linked receptor protein signaling pathway	7/94	337/14350	7	0.00661697	0.175054	0.175054
124	BP	GO:0051251	positive regulation of lymphocyte activation	3/94	59/14350	3	0.0067894	0.178167	0.178167
125	BP	GO:1903037	regulation of leukocyte cell-cell adhesion	3/94	61/14350	3	0.00744616	0.193838	0.193838
126	BP	GO:0051240	positive regulation of multicellular organismal process	5/94	186/14350	5	0.00751296	0.194025	0.194025
127	BP	GO:0016311	dephosphorylation	5/94	189/14350	5	0.00802252	0.204225	0.204225
128	BP	GO:0051239	regulation of multicellular organismal process	8/94	440/14350	8	0.00818929	0.204225	0.204225
129	BP	GO:0001666	response to hypoxia	2/94	21/14350	2	0.00822173	0.204225	0.204225
130	BP	GO:0036293	response to decreased oxygen levels	2/94	21/14350	2	0.00822173	0.204225	0.204225
131	BP	GO:0048545	response to steroid hormone	2/94	21/14350	2	0.00822173	0.204225	0.204225
132	BP	GO:0010628	positive regulation of gene expression	4/94	122/14350	4	0.00842367	0.207656	0.207656
133	BP	GO:0045785	positive regulation of cell adhesion	3/94	64/14350	3	0.00849963	0.207953	0.207953
134	BP	GO:0050863	regulation of T cell activation	3/94	66/14350	3	0.00924812	0.224578	0.224578
135	BP	GO:0002696	positive regulation of leukocyte activation	3/94	67/14350	3	0.00963637	0.230564	0.230564
136	BP	GO:0050867	positive regulation of cell activation	3/94	67/14350	3	0.00963637	0.230564	0.230564

No.	Ontology	GO ID	Description	GeneRatio	BgRatio	Count	P value	Adjusted P	q value
137	BP	GO:0022407	regulation of cell-cell adhesion	3/94	71/14350	3	0.0112838	0.26801	0.26801
138	BP	GO:0046649	lymphocyte activation	4/94	134/14350	4	0.0116081	0.273715	0.273715
139	BP	GO:0070482	response to oxygen levels	2/94	26/14350	2	0.0124572	0.291624	0.291624
140	BP	GO:0045216	cell-cell junction organization	3/94	75/14350	3	0.013084	0.30411	0.30411
141	BP	GO:0002429	immune response-activating cell surface receptor signaling pathway	3/94	76/14350	3	0.0135582	0.312896	0.312896
142	BP	GO:0043297	apical junction assembly	2/94	28/14350	2	0.0143666	0.326915	0.326915
143	BP	GO:0070830	bicellular tight junction assembly	2/94	28/14350	2	0.0143666	0.326915	0.326915
144	BP	GO:0030198	extracellular matrix organization	3/94	79/14350	3	0.0150392	0.337499	0.337499
145	BP	GO:0045229	external encapsulating structure organization	3/94	79/14350	3	0.0150392	0.337499	0.337499
146	BP	GO:0120192	tight junction assembly	2/94	29/14350	2	0.0153657	0.340135	0.340135
147	BP	GO:0120193	tight junction organization	2/94	29/14350	2	0.0153657	0.340135	0.340135
148	BP	GO:0002768	immune response-regulating cell surface receptor signaling pathway	3/94	81/14350	3	0.0160753	0.349725	0.349725
149	BP	GO:0043062	extracellular structure organization	3/94	81/14350	3	0.0160753	0.349725	0.349725
150	BP	GO:0007169	cell surface receptor protein tyrosine kinase signaling pathway	5/94	225/14350	5	0.0161213	0.349725	0.349725
151	BP	GO:0050730	regulation of peptidyl-tyrosine phosphorylation	2/94	30/14350	2	0.0163938	0.350956	0.350956
152	BP	GO:2001234	negative regulation of apoptotic signaling pathway	2/94	30/14350	2	0.0163938	0.350956	0.350956
153	BP	GO:0051249	regulation of lymphocyte activation	3/94	83/14350	3	0.0171508	0.364762	0.364762
154	BP	GO:0045321	leukocyte activation	4/94	154/14350	4	0.0184648	0.381736	0.381736
155	BP	GO:1901701	cellular response to oxygen-containing compound	4/94	154/14350	4	0.0184648	0.381736	0.381736
156	BP	GO:0007179	transforming growth factor beta receptor signaling pathway	2/94	32/14350	2	0.0185354	0.381736	0.381736
157	BP	GO:0071559	response to transforming growth factor beta	2/94	32/14350	2	0.0185354	0.381736	0.381736
158	BP	GO:0071560	cellular response to transforming growth factor beta stimulus	2/94	32/14350	2	0.0185354	0.381736	0.381736
159	BP	GO:0043549	regulation of kinase activity	4/94	156/14350	4	0.019263	0.394225	0.394225
160	BP	GO:0098609	cell-cell adhesion	6/94	325/14350	6	0.0198776	0.40426	0.40426
161	BP	GO:0006469	negative regulation of protein kinase activity	2/94	35/14350	2	0.0219554	0.441006	0.441006
162	BP	GO:0033627	cell adhesion mediated by integrin	2/94	35/14350	2	0.0219554	0.441006	0.441006
163	BP	GO:0019216	regulation of lipid metabolic process	2/94	36/14350	2	0.0231492	0.459314	0.459314
164	BP	GO:0033673	negative regulation of kinase activity	2/94	36/14350	2	0.0231492	0.459314	0.459314
165	BP	GO:0034330	cell junction organization	4/94	166/14350	4	0.0235737	0.462102	0.462102
166	BP	GO:0051338	regulation of transferase activity	4/94	166/14350	4	0.0235737	0.462102	0.462102
167	BP	GO:0001819	positive regulation of cytokine production	2/94	37/14350	2	0.0243691	0.472007	0.472007
168	BP	GO:0048871	multicellular organismal-level homeostasis	2/94	37/14350	2	0.0243691	0.472007	0.472007
169	BP	GO:0002694	regulation of leukocyte activation	3/94	96/14350	3	0.025105	0.483383	0.483383
170	BP	GO:0050852	T cell receptor signaling pathway	2/94	38/14350	2	0.0256149	0.487432	0.487432
171	BP	GO:0071222	cellular response to lipopolysaccharide	2/94	38/14350	2	0.0256149	0.487432	0.487432
172	MF	GO:0004879	nuclear receptor activity	10/99	51/14823	10	1.130E-12	6.533E-10	6.533E-10
173	MF	GO:0098531	ligand-modulated transcription factor activity	10/99	51/14823	10	1.130E-12	6.533E-10	6.533E-10
174	MF	GO:0005126	cytokine receptor binding	11/99	138/14823	11	1.911E-09	7.363E-07	7.363E-07
175	MF	GO:0004713	protein tyrosine kinase activity	7/99	99/14823	7	4.312E-06	0.0012463	0.0012463
176	MF	GO:0005125	cytokine activity	8/99	147/14823	8	6.180E-06	0.00142876	0.00142876
177	MF	GO:0020037	heme binding	7/99	110/14823	7	8.684E-06	0.00167303	0.00167303
178	MF	GO:0046906	tetrapyrrole binding	7/99	115/14823	7	1.164E-05	0.00192155	0.00192155
179	MF	GO:0038187	pattern recognition receptor activity	3/99	8/14823	3	1.580E-05	0.00228244	0.00228244
180	MF	GO:0004715	non-membrane spanning protein tyrosine kinase activity	4/99	29/14823	4	3.911E-05	0.0050236	0.0050236
181	MF	GO:0048018	receptor ligand activity	9/99	314/14823	9	2.494E-04	0.0274668	0.0274668
182	MF	GO:0030546	signaling receptor activator activity	9/99	316/14823	9	2.614E-04	0.0274668	0.0274668
183	MF	GO:0030545	signaling receptor regulator activity	9/99	341/14823	9	4.561E-04	0.0413004	0.0413004
184	MF	GO:0005164	tumor necrosis factor receptor binding	3/99	23/14823	3	4.645E-04	0.0413004	0.0413004
185	MF	GO:0032813	tumor necrosis factor receptor superfamily binding	3/99	30/14823	3	0.00102929	0.0849897	0.0849897
186	MF	GO:0016705	oxidoreductase activity, acting on paired donors, with incorporation or reduction of	5/99	143/14823	5	0.00269549	0.189725	0.189725

No.	Ontology	GO ID	Description	GeneRatio	BgRatio	Count	P value	Adjusted P	q value
			molecular oxygen						
187	MF	GO:0010181	FMN binding	2/99	12/14823	2	0.00279007	0.189725	0.189725
188	MF	GO:0070403	D+ binding	2/99	12/14823	2	0.00279007	0.189725	0.189725
189	MF	GO:0004497	monooxygenase activity	4/99	90/14823	4	0.00308379	0.198048	0.198048
190	MF	GO:0004707	MAP kinase activity	2/99	14/14823	2	0.00381359	0.232027	0.232027
191	MF	GO:0003707	nuclear steroid receptor activity	2/99	15/14823	2	0.00438121	0.241175	0.241175
192	MF	GO:0016709	oxidoreductase activity, acting on paired donors, with incorporation or reduction of molecular oxygen, D(P)H as one donor, and incorporation of one atom of oxygen	2/99	15/14823	2	0.00438121	0.241175	0.241175
193	MF	GO:0070851	growth factor receptor binding	3/99	55/14823	3	0.00589649	0.309834	0.309834
194	MF	GO:0004725	protein tyrosine phosphatase activity	3/99	57/14823	3	0.00651358	0.327378	0.327378
195	MF	GO:0016702	oxidoreductase activity, acting on single donors with incorporation of molecular oxygen, incorporation of two atoms of oxygen	2/99	23/14823	2	0.0101967	0.49114	0.49114

Table S2E. Complete GO enrichment results: enrichment metrics and contributing genes (linked to Table S2D by row number/GO ID).

No.	GO ID	Rich factor	Fold enrichment	z score	Contributing genes
1	GO:0006952	0.0606061	9.2521	13.5219	ELANE; NLRP3; TLR4; PPARG; PTPN22; STAT3; CXCL8; SYK; TLR9; RORA; CRP; IL1B; TLR2; IL17A; PGLYRP2; LYN; RELA; NFKB1; S100A8; LTF; IL33; MYD88; STAT1; NR1H4
2	GO:0009605	0.0475113	7.25306	10.8429	NLRP3; TLR4; PPARG; PTPN22; CXCL8; SYK; TLR9; RORA; CRP; IL1B; TLR2; PGLYRP2; LYN; RELA; NFKB1; S100A8; LTF; IL33; MYD88; STAT1; NR1H4
3	GO:0006954	0.0813953	12.4258	12.241	ELANE; NLRP3; TLR4; PPARG; CXCL8; RORA; IL1B; TLR2; IL17A; RELA; NFKB1; S100A8; IL33; NR1H4
4	GO:0043207	0.0553633	8.45174	10.3914	TLR4; PTPN22; CXCL8; SYK; TLR9; CRP; IL1B; TLR2; PGLYRP2; LYN; RELA; NFKB1; S100A8; LTF; MYD88; STAT1
5	GO:0051707	0.0553633	8.45174	10.3914	TLR4; PTPN22; CXCL8; SYK; TLR9; CRP; IL1B; TLR2; PGLYRP2; LYN; RELA; NFKB1; S100A8; LTF; MYD88; STAT1
6	GO:0009607	0.0549828	8.39365	10.3468	TLR4; PTPN22; CXCL8; SYK; TLR9; CRP; IL1B; TLR2; PGLYRP2; LYN; RELA; NFKB1; S100A8; LTF; MYD88; STAT1
7	GO:0044419	0.0546075	8.33636	10.3025	TLR4; PTPN22; CXCL8; SYK; TLR9; CRP; IL1B; TLR2; PGLYRP2; LYN; RELA; NFKB1; S100A8; LTF; MYD88; STAT1
8	GO:0140546	0.0599078	9.1455	9.81763	TLR4; PTPN22; CXCL8; SYK; TLR9; CRP; TLR2; LYN; RELA; NFKB1; LTF; MYD88; STAT1
9	GO:0098542	0.0528302	8.06503	9.42616	TLR4; PTPN22; CXCL8; SYK; TLR9; CRP; TLR2; PGLYRP2; LYN; RELA; NFKB1; LTF; MYD88; STAT1
10	GO:0031347	0.0934579	14.2672	11.1853	NLRP3; TLR4; PPARG; PTPN22; TLR9; RORA; TLR2; IL33; MYD88; NR1H4
11	GO:0045087	0.0597826	9.12639	9.00863	TLR4; PTPN22; SYK; TLR9; CRP; TLR2; LYN; RELA; NFKB1; MYD88; STAT1
12	GO:0032101	0.0636943	9.72354	8.92445	NLRP3; TLR4; PPARG; PTPN22; TLR9; RORA; TLR2; IL33; MYD88; NR1H4
13	GO:0080134	0.0584795	8.92746	8.46809	NLRP3; TLR4; PPARG; PTPN22; TLR9; RORA; TLR2; IL33; MYD88; NR1H4
14	GO:0042127	0.0552486	8.43423	8.173	CAV1; EGFR; PTPN22; FGFR1; STAT3; SYK; IL6; IL1B; TGFB1; STAT1
15	GO:0008283	0.047619	7.2695	7.4318	CAV1; EGFR; PTPN22; FGFR1; STAT3; SYK; IL6; IL1B; TGFB1; STAT1
16	GO:0002221	0.166667	25.4433	10.8824	TLR4; PTPN22; TLR9; TLR2; MYD88
17	GO:0034097	0.0517241	7.89618	7.4316	JAK1; STAT3; CXCL8; TNF; IL1B; RELA; NFKB1; JAK2; STAT1
18	GO:1901652	0.0517241	7.89618	7.4316	JAK1; STAT3; CXCL8; TNF; IL1B; RELA; NFKB1; JAK2; STAT1
19	GO:0038061	0.266667	40.7092	12.4944	NLRP3; PTPN22; RELA; NFKB1
20	GO:0031349	0.1	15.266	8.99159	TLR4; PTPN22; TLR9; TLR2; IL33; MYD88
21	GO:0009617	0.0693069	10.5804	7.84561	TLR4; CXCL8; IL1B; PGLYRP2; S100A8; LTF; MYD88
22	GO:0030097	0.0693069	10.5804	7.84561	JAK1; PTPN22; SYK; CASP3; IL6; IL1B; JAK2
23	GO:0002758	0.131579	20.0868	9.56644	TLR4; PTPN22; TLR9; TLR2; MYD88
24	GO:0002757	0.0686275	10.4766	7.79926	PTPRC; TLR4; PTPN22; SYK; TLR9; TLR2; MYD88
25	GO:0002764	0.0660377	10.0813	7.6201	PTPRC; TLR4; PTPN22; SYK; TLR9; TLR2; MYD88
26	GO:0002218	0.119048	18.1738	9.05058	TLR4; PTPN22; TLR9; TLR2; MYD88
27	GO:0050728	0.2	30.5319	10.7315	NLRP3; PPARG; RORA; NR1H4
28	GO:0050727	0.111111	16.9622	8.70824	NLRP3; PPARG; RORA; IL33; NR1H4
29	GO:0010557	0.0301508	4.6028	5.91888	PPARG; PTPN22; NR1I2; TLR9; CTNNB1; TNF; IL1B; BRCA1; THRA; VDR; IL33; NR1H4
30	GO:0002224	0.181818	27.7563	10.1981	TLR4; TLR9; TLR2; MYD88
31	GO:0009891	0.0295567	4.51211	5.82925	PPARG; PTPN22; NR1I2; TLR9; CTNNB1; TNF; IL1B; BRCA1; THRA; VDR; IL33; NR1H4
32	GO:0031348	0.173913	26.5495	9.95737	NLRP3; PPARG; RORA; NR1H4
33	GO:0002253	0.056	8.54894	6.8832	PTPRC; TLR4; PTPN22; SYK; TLR9; TLR2; MYD88
34	GO:0032103	0.0714286	10.9043	7.39241	TLR4; PTPN22; TLR9; TLR2; IL33; MYD88
35	GO:0045089	0.0943396	14.4018	7.93699	TLR4; PTPN22; TLR9; TLR2; MYD88

No.	GO ID	Rich factor	Fold enrichment	z score	Contributing genes
36	GO:0002833	0.0877193	13.3912	7.6114	TLR4; PTPN22; TLR9; TLR2; MYD88
37	GO:0045944	0.0418848	6.39412	6.09392	PPARG; NR1I2; CTNNB1; TNF; BRCA1; THRA; VDR; NR1H4
38	GO:0045088	0.0862069	13.1603	7.53508	TLR4; PTPN22; TLR9; TLR2; MYD88
39	GO:0008284	0.0576923	8.80728	6.48853	EGFR; PTPN22; FGFR1; SYK; IL6; IL1B
40	GO:0019221	0.0460526	7.03038	6.06916	JAK1; STAT3; CXCL8; TNF; IL1B; JAK2; STAT1
41	GO:0002831	0.0769231	11.743	7.04888	TLR4; PTPN22; TLR9; TLR2; MYD88
42	GO:0070887	0.0293255	4.47682	5.27633	PPARG; STAT3; CXCL8; HIF1A; ESR2; IL1B; THRA; S100A8; NFE2L2; ESR1
43	GO:0071345	0.0440252	6.72086	5.89019	JAK1; STAT3; CXCL8; TNF; IL1B; JAK2; STAT1
44	GO:0009719	0.032491	4.96006	5.40411	PPARG; FGFR1; STAT3; ESR2; THRA; TGFB1; STAT1; ESR1; SMAD3
45	GO:0008625	0.214286	32.7128	9.63962	TNF; CASP8; FASLG
46	GO:0046631	0.214286	32.7128	9.63962	PTPN22; SYK; IL6
47	GO:0006915	0.03125	4.77061	5.2488	TP53; EGFR; CASP3; TNF; APOE; TJP1; CASP8; BCL2; FASLG
48	GO:0009755	0.102564	15.6574	7.44269	PPARG; ESR2; THRA; ESR1
49	GO:0012501	0.0306122	4.67325	5.16737	TP53; EGFR; CASP3; TNF; APOE; TJP1; CASP8; BCL2; FASLG
50	GO:0008219	0.0305085	4.65741	5.15401	TP53; EGFR; CASP3; TNF; APOE; TJP1; CASP8; BCL2; FASLG
51	GO:0050778	0.0404624	6.17698	5.56267	PTPRC; TLR4; PTPN22; SYK; TLR9; TLR2; MYD88
52	GO:0042102	0.1875	28.6237	8.97705	PTPN22; SYK; IL1B
53	GO:0007249	0.0649351	9.91296	6.36774	TLR9; RELA; NFKB1; MYD88; FASLG
54	GO:0009725	0.0487805	7.44681	5.83064	PPARG; STAT3; ESR2; THRA; STAT1; ESR1
55	GO:0033993	0.0641026	9.78587	6.3178	CXCL8; ESR2; IL1B; S100A8; ESR1
56	GO:0097191	0.0952381	14.539	7.13506	TNF; CASP8; BCL2; FASLG
57	GO:0007259	0.0909091	13.8781	6.94699	JAK1; STAT3; JAK2; STAT1
58	GO:0018108	0.0909091	13.8781	6.94699	PTPRC; CAV1; JAK1; JAK2
59	GO:0097696	0.0909091	13.8781	6.94699	JAK1; STAT3; JAK2; STAT1
60	GO:0002684	0.0323887	4.94444	5.07755	PTPRC; TLR4; PTPN22; SYK; TLR9; IL1B; TLR2; MYD88
61	GO:0018212	0.0869565	13.2747	6.77078	PTPRC; CAV1; JAK1; JAK2
62	GO:0032946	0.15	22.8989	7.95776	PTPN22; SYK; IL1B
63	GO:0050671	0.15	22.8989	7.95776	PTPN22; SYK; IL1B
64	GO:0070665	0.15	22.8989	7.95776	PTPN22; SYK; IL1B
65	GO:0050776	0.0358974	5.48009	5.11475	PTPRC; TLR4; PTPN22; SYK; TLR9; TLR2; MYD88
66	GO:0032102	0.08	12.2128	6.4492	NLRP3; PPARG; RORA; NR1H4
67	GO:0071495	0.0304183	4.64364	4.84261	PPARG; FGFR1; STAT3; ESR2; THRA; TGFB1; ESR1; SMAD3
68	GO:0030098	0.0784314	11.9733	6.37449	PTPN22; SYK; IL6; IL1B
69	GO:0006809	0.4	61.0638	10.9075	NOS3; NOS2
70	GO:0046209	0.4	61.0638	10.9075	NOS3; NOS2
71	GO:0046633	0.4	61.0638	10.9075	PTPN22; SYK
72	GO:0046640	0.4	61.0638	10.9075	PTPN22; SYK
73	GO:0030217	0.130435	19.9121	7.37059	PTPN22; IL6; IL1B
74	GO:0030574	0.130435	19.9121	7.37059	MMP9; MMP2; MMP14
75	GO:0006979	0.0727273	11.1025	6.09529	HMOX1; LPO; PTGS2; NFE2L2
76	GO:1903131	0.0727273	11.1025	6.09529	PTPN22; SYK; IL6; IL1B
77	GO:0045893	0.028777	4.39308	4.6389	PPARG; NR1I2; CTNNB1; TNF; BRCA1; THRA; VDR; NR1H4
78	GO:1902680	0.028777	4.39308	4.6389	PPARG; NR1I2; CTNNB1; TNF; BRCA1; THRA; VDR; NR1H4
79	GO:0042742	0.0714286	10.9043	6.02997	TLR4; PGLYRP2; LTF; MYD88
80	GO:0030522	0.12	18.3191	7.03761	PTPN22; ESR2; THRA
81	GO:0032963	0.115385	17.6146	6.88525	MMP9; MMP2; MMP14
82	GO:0032870	0.0458716	7.00273	5.1082	PPARG; STAT3; ESR2; THRA; ESR1
83	GO:0042129	0.107143	16.3564	6.60453	PTPN22; SYK; IL1B
84	GO:0071396	0.0615385	9.39444	5.50787	CXCL8; ESR2; IL1B; ESR1
85	GO:0002526	0.285714	43.617	9.15773	ELANE; NLRP3

No.	GO ID	Rich factor	Fold enrichment	z score	Contributing genes
86	GO:2001057	0.285714	43.617	9.15773	NOS3; NOS2
87	GO:0042098	0.103448	15.7924	6.47479	PTPN22; SYK; IL1B
88	GO:0002521	0.0588235	8.97997	5.35596	PTPN22; SYK; IL6; IL1B
89	GO:0051254	0.0251572	3.84049	4.15933	PPARG; NR1I2; CTNNB1; TNF; BRCA1; THRA; VDR; NR1H4
90	GO:1901222	0.25	38.1649	8.53786	NLRP3; PTPN22
91	GO:0007159	0.0555556	8.48109	5.16741	PTPN22; SYK; IL1B; S100A8
92	GO:0032944	0.0882353	13.47	5.91112	PTPN22; SYK; IL1B
93	GO:0050670	0.0882353	13.47	5.91112	PTPN22; SYK; IL1B
94	GO:0070663	0.0882353	13.47	5.91112	PTPN22; SYK; IL1B
95	GO:0046634	0.222222	33.9243	8.02278	PTPN22; SYK
96	GO:1901700	0.0314136	4.79559	4.28801	STAT3; CXCL8; ESR2; IL1B; S100A8; STAT1
97	GO:0002682	0.0233918	3.57098	3.90751	PTPRC; TLR4; PTPN22; SYK; TLR9; IL1B; TLR2; MYD88
98	GO:0051341	0.2	30.5319	7.58565	IL1B; APOE
99	GO:0043066	0.0487805	7.44681	4.75386	EGFR; APOE; CASP8; BCL2
100	GO:0043069	0.0481928	7.35709	4.71636	EGFR; APOE; CASP8; BCL2
101	GO:0043401	0.181818	27.7563	7.2084	ESR2; ESR1
102	GO:0042981	0.0291262	4.4464	4.04565	EGFR; CASP3; APOE; TJP1; CASP8; BCL2
103	GO:0043067	0.0287081	4.38257	3.99996	EGFR; CASP3; APOE; TJP1; CASP8; BCL2
104	GO:0032496	0.0714286	10.9043	5.21955	CXCL8; IL1B; S100A8
105	GO:0048584	0.0200445	3.05999	3.60115	PTPRC; TLR4; PTPN22; SYK; TLR9; TLR2; IL33; MYD88; FASLG
106	GO:0097190	0.0425532	6.49615	4.3411	TNF; CASP8; BCL2; FASLG
107	GO:0002237	0.0652174	9.95606	4.94018	CXCL8; IL1B; S100A8
108	GO:0050870	0.0638298	9.74423	4.87565	PTPN22; SYK; IL1B
109	GO:1903039	0.0638298	9.74423	4.87565	PTPN22; SYK; IL1B
110	GO:0048585	0.0232558	3.55022	3.6309	NLRP3; PPARG; PTPN22; RORA; CASP8; BCL2; NR1H4
111	GO:0042110	0.039604	6.04592	4.13224	PTPN22; SYK; IL6; IL1B
112	GO:0007155	0.0185185	2.82703	3.32733	PTPN22; CLDN2; SYK; CTNNB1; ITGB3; IL1B; S100A8; TJP1; THBS1
113	GO:0022409	0.0576923	8.80728	4.57973	PTPN22; SYK; IL1B
114	GO:0050678	0.125	19.0824	5.87637	CAV1; EGFR
115	GO:0032943	0.0566038	8.64111	4.5253	PTPN22; SYK; IL1B
116	GO:0046651	0.0566038	8.64111	4.5253	PTPN22; SYK; IL1B
117	GO:0050851	0.0566038	8.64111	4.5253	PTPRC; PTPN22; SYK
118	GO:0070661	0.0566038	8.64111	4.5253	PTPN22; SYK; IL1B
119	GO:0050673	0.117647	17.9599	5.68141	CAV1; EGFR
120	GO:0050830	0.111111	16.9622	5.50238	PGLYRP2; MYD88
121	GO:0071383	0.111111	16.9622	5.50238	ESR2; ESR1
122	GO:0141193	0.111111	16.9622	5.50238	ESR2; THRA
123	GO:0007167	0.0207715	3.17097	3.27476	EGFR; FGFR1; STAT3; SYK; LYN; TGFB1; SMAD3
124	GO:0051251	0.0508475	7.76235	4.22638	PTPN22; SYK; IL1B
125	GO:1903037	0.0491803	7.50785	4.13597	PTPN22; SYK; IL1B
126	GO:0051240	0.0268817	4.10375	3.4596	PTPN22; SYK; TLR9; IL1B; IL33
127	GO:0016311	0.026455	4.03861	3.41456	PTPRC; PTPN22; ALPI; PSPH; PTPN2
128	GO:0051239	0.0181818	2.77563	3.07178	PTPN22; SYK; TLR9; IL1B; ENPP1; APOE; IL33; THBS1
129	GO:0001666	0.0952381	14.539	5.04155	HIF1A; MMP2
130	GO:0036293	0.0952381	14.539	5.04155	HIF1A; MMP2
131	GO:0048545	0.0952381	14.539	5.04155	ESR2; ESR1
132	GO:0010628	0.0327869	5.00523	3.60754	PTPN22; TLR9; IL1B; IL33
133	GO:0045785	0.046875	7.15592	4.00778	PTPN22; SYK; IL1B
134	GO:0050863	0.0454545	6.93907	3.92682	PTPN22; SYK; IL1B
135	GO:0002696	0.0447761	6.8355	3.8876	PTPN22; SYK; IL1B

No.	GO ID	Rich factor	Fold enrichment	z score	Contributing genes
136	GO:0050867	0.0447761	6.8355	3.8876	PTPN22; SYK; IL1B
137	GO:0022407	0.0422535	6.4504	3.73839	PTPN22; SYK; IL1B
138	GO:0046649	0.0298507	4.557	3.35911	PTPN22; SYK; IL6; IL1B
139	GO:0070482	0.0769231	11.743	4.45203	HIF1A; MMP2
140	GO:0045216	0.04	6.10638	3.60024	CLDN2; OCLN; TJP1
141	GO:0002429	0.0394737	6.02604	3.56727	PTPRC; PTPN22; SYK
142	GO:0043297	0.0714286	10.9043	4.25966	CLDN2; OCLN
143	GO:0070830	0.0714286	10.9043	4.25966	CLDN2; OCLN
144	GO:0030198	0.0379747	5.7972	3.47176	MMP9; MMP2; MMP14
145	GO:0045229	0.0379747	5.7972	3.47176	MMP9; MMP2; MMP14
146	GO:0120192	0.0689655	10.5282	4.17063	CLDN2; OCLN
147	GO:0120193	0.0689655	10.5282	4.17063	CLDN2; OCLN
148	GO:0002768	0.037037	5.65406	3.41078	PTPRC; PTPN22; SYK
149	GO:0043062	0.037037	5.65406	3.41078	MMP9; MMP2; MMP14
150	GO:0007169	0.0222222	3.39243	2.93706	EGFR; FGFR1; STAT3; SYK; LYN
151	GO:0050730	0.0666667	10.1773	4.08583	PTPRC; CAV1
152	GO:2001234	0.0666667	10.1773	4.08583	CASP8; BCL2
153	GO:0051249	0.0361446	5.51782	3.35179	PTPN22; SYK; IL1B
154	GO:0045321	0.025974	3.96518	3.00403	PTPN22; SYK; IL6; IL1B
155	GO:1901701	0.025974	3.96518	3.00403	STAT3; CXCL8; ESR2; IL1B
156	GO:0007179	0.0625	9.54122	3.92762	TGFB1; SMAD3
157	GO:0071559	0.0625	9.54122	3.92762	TGFB1; SMAD3
158	GO:0071560	0.0625	9.54122	3.92762	TGFB1; SMAD3
159	GO:0043549	0.025641	3.91435	2.97185	PTPRC; CAV1; EGFR; FGFR1
160	GO:0098609	0.0184615	2.81833	2.6924	PTPN22; CLDN2; SYK; IL1B; S100A8; TJP1
161	GO:0006469	0.0571429	8.7234	3.71469	PTPRC; CAV1
162	GO:0033627	0.0571429	8.7234	3.71469	SYK; ITGB3
163	GO:0019216	0.0555556	8.48109	3.64932	PPARG; BRCA1
164	GO:0033673	0.0555556	8.48109	3.64932	PTPRC; CAV1
165	GO:0034330	0.0240964	3.67854	2.81857	CLDN2; OCLN; TJP1; CACNB4
166	GO:0051338	0.0240964	3.67854	2.81857	PTPRC; CAV1; EGFR; FGFR1
167	GO:0001819	0.0540541	8.25187	3.58642	TLR9; IL33
168	GO:0048871	0.0540541	8.25187	3.58642	CASP3; TJP1
169	GO:0002694	0.03125	4.77061	3.00992	PTPN22; SYK; IL1B
170	GO:0050852	0.0526316	8.03471	3.52585	PTPRC; PTPN22
171	GO:0071222	0.0526316	8.03471	3.52585	CXCL8; IL1B
172	GO:0004879	0.196078	29.3583	16.6343	PPARG; NR1I2; RORA; ESR2; THRA; VDR; RORC; NR1H4; ESR1; AHR
173	GO:0098531	0.196078	29.3583	16.6343	PPARG; NR1I2; RORA; ESR2; THRA; VDR; RORC; NR1H4; ESR1; AHR
174	GO:0005126	0.0797101	11.9348	10.5821	JAK1; CXCL8; IFNG; TNF; IL6; IL1B; TNFSF11; JAK2; IL2; TGFB1; FASLG
175	GO:0004713	0.0707071	10.5868	7.84757	CLK1; EGFR; JAK1; FGFR1; SYK; LYN; JAK2
176	GO:0005125	0.0544218	8.14842	7.14205	CXCL8; TNF; IL6; IL1B; IL17A; TGFB1; IL33; IL10
177	GO:0020037	0.0636364	9.5281	7.36133	HMOX1; NOS3; IDO1; NOS2; CYP19A1; LPO; PTGS2
178	GO:0046906	0.0608696	9.11383	7.16236	HMOX1; NOS3; IDO1; NOS2; CYP19A1; LPO; PTGS2
179	GO:0038187	0.375	56.1477	12.7932	TLR4; TLR9; PGLYRP2
180	GO:0004715	0.137931	20.652	8.68604	JAK1; SYK; LYN; JAK2
181	GO:0048018	0.0286624	4.29155	4.83397	CXCL8; TNF; IL6; IL1B; IL17A; IL2; TGFB1; IL33; IL10
182	GO:0030546	0.028481	4.26438	4.80966	CXCL8; TNF; IL6; IL1B; IL17A; IL2; TGFB1; IL33; IL10
183	GO:0030545	0.026393	3.95175	4.52168	CXCL8; TNF; IL6; IL1B; IL17A; IL2; TGFB1; IL33; IL10
184	GO:0005164	0.130435	19.5296	7.29219	TNF; TNFSF11; FASLG
185	GO:0032813	0.1	14.9727	6.28162	TNF; TNFSF11; FASLG

No.	GO ID	Rich factor	Fold enrichment	z score	Contributing genes
186	GO:0016705	0.034965	5.23522	4.17291	HMOX1; NOS3; NOS2; CYP19A1; PTGS2
187	GO:0010181	0.166667	24.9545	6.80682	NOS3; NOS2
188	GO:0070403	0.166667	24.9545	6.80682	SIRT1; SIRT6
189	GO:0004497	0.0444444	6.65455	4.41196	HMOX1; NOS3; NOS2; CYP19A1
190	GO:0004707	0.142857	21.3896	6.25847	MAPK1; MAPK3
191	GO:0003707	0.133333	19.9636	6.02528	ESR2; ESR1
192	GO:0016709	0.133333	19.9636	6.02528	NOS3; NOS2
193	GO:0070851	0.0545455	8.16694	4.36629	IL6; IL1B; IL2
194	GO:0004725	0.0526316	7.88038	4.26753	PTPRC; PTPN22; PTPN2
195	GO:0016702	0.0869565	13.0198	4.73028	IDO1; PTGS2

Table S2F. Complete KEGG enrichment results: pathway classification, ratios, counts, and significance statistics.

No.	Category	Subcategory	KEGG ID	Pathway	GeneRatio	BgRatio	Count	P value	Adjusted P	q value
1	Human Diseases	Infectious disease: viral	fca05161	Hepatitis B	25/96	162/10177	25	4.834E-24	1.063E-21	1.063E-21
2	Organismal Systems	Immune system	fca04659	Th17 cell differentiation	22/96	109/10177	22	6.899E-24	1.063E-21	1.063E-21
3	Human Diseases	Cardiovascular disease	fca05417	Lipid and atherosclerosis	27/96	210/10177	27	8.910E-24	1.063E-21	1.063E-21
4	Human Diseases	Immune disease	fca05321	Inflammatory bowel disease	18/96	64/10177	18	1.668E-22	1.493E-20	1.493E-20
5	Human Diseases	Infectious disease: parasitic	fca05140	Leishmaniasis	18/96	75/10177	18	4.085E-21	2.884E-19	2.884E-19
6	Human Diseases	Infectious disease: parasitic	fca05142	Chagas disease	20/96	107/10177	20	4.834E-21	2.884E-19	2.884E-19
7	Human Diseases	Infectious disease: parasitic	fca05145	Toxoplasmosis	20/96	112/10177	20	1.272E-20	5.987E-19	5.987E-19
8	Human Diseases	Infectious disease: bacterial	fca05152	Tuberculosis	23/96	173/10177	23	1.338E-20	5.987E-19	5.987E-19
9	Human Diseases	Endocrine and metabolic disease	fca04933	AGE-RAGE signaling pathway in diabetic complications	19/96	102/10177	19	5.462E-20	2.173E-18	2.173E-18
10	Human Diseases	Cancer: overview	fca05205	Proteoglycans in cancer	23/96	210/10177	23	1.179E-18	4.219E-17	4.219E-17
11	Human Diseases	Infectious disease: viral	fca05164	Influenza A	21/96	163/10177	21	1.496E-18	4.870E-17	4.870E-17
12	Human Diseases	Cancer: overview	fca05235	PD-L1 expression and PD-1 checkpoint pathway in cancer	17/96	90/10177	17	4.542E-18	1.355E-16	1.355E-16
13	Human Diseases	Infectious disease: viral	fca05167	Kaposi sarcoma-associated herpesvirus infection	22/96	199/10177	22	6.000E-18	1.652E-16	1.652E-16
14	Human Diseases	Infectious disease: viral	fca05160	Hepatitis C	20/96	155/10177	20	1.046E-17	2.579E-16	2.579E-16
15	Human Diseases	Infectious disease: viral	fca05168	Herpes simplex virus 1 infection	21/96	179/10177	21	1.081E-17	2.579E-16	2.579E-16
16	Human Diseases	Infectious disease: viral	fca05163	Human cytomegalovirus infection	22/96	226/10177	22	9.444E-17	2.113E-15	2.113E-15
17	Human Diseases	Infectious disease: viral	fca05162	Measles	18/96	132/10177	18	1.930E-16	4.064E-15	4.064E-15
18	Human Diseases	Cancer: overview	fca05206	MicroRs in cancer	19/96	161/10177	19	4.106E-16	8.167E-15	8.167E-15
19	Organismal Systems	Immune system	fca04657	IL-17 signaling pathway	16/96	98/10177	16	5.445E-16	1.026E-14	1.026E-14
20	Organismal Systems	Immune system	fca04620	Toll-like receptor signaling pathway	16/96	101/10177	16	8.983E-16	1.608E-14	1.608E-14
21	Organismal Systems	Immune system	fca04625	C-type lectin receptor signaling pathway	16/96	104/10177	16	1.458E-15	2.485E-14	2.485E-14
22	Human Diseases	Infectious disease: bacterial	fca05133	Pertussis	14/96	76/10177	14	7.076E-15	1.151E-13	1.151E-13
23	Human Diseases	Cancer: specific types	fca05212	Pancreatic cancer	14/96	77/10177	14	8.583E-15	1.301E-13	1.301E-13
24	Environmental Information Processing	Signal transduction	fca04066	HIF-1 signaling pathway	16/96	116/10177	16	8.725E-15	1.301E-13	1.301E-13
25	Human Diseases	Infectious disease: bacterial	fca05134	Legionellosis	13/96	65/10177	13	2.227E-14	3.190E-13	3.190E-13
26	Human Diseases	Infectious disease: parasitic	fca05144	Malaria	12/96	51/10177	12	2.873E-14	3.955E-13	3.955E-13
27	Environmental Information Processing	Signal transduction	fca04151	PI3K-Akt signaling pathway	23/96	354/10177	23	1.179E-13	1.563E-12	1.563E-12
28	Organismal Systems	Immune system	fca04621	NOD-like receptor signaling pathway	17/96	163/10177	17	1.356E-13	1.733E-12	1.733E-12
29	Human Diseases	Infectious disease: viral	fca05169	Epstein-Barr virus infection	18/96	201/10177	18	3.453E-13	4.263E-12	4.263E-12
30	Human Diseases	Cancer: specific types	fca05215	Prostate cancer	14/96	103/10177	14	5.772E-13	6.887E-12	6.887E-12
31	Human Diseases	Infectious disease: parasitic	fca05146	Amoebiasis	14/96	104/10177	14	6.619E-13	7.464E-12	7.464E-12
32	Organismal Systems	Development and regeneration	fca04380	Osteoclast differentiation	15/96	127/10177	15	6.672E-13	7.464E-12	7.464E-12
33	Human Diseases	Endocrine and metabolic disease	fca04936	Alcoholic liver disease	15/96	136/10177	15	1.847E-12	2.003E-11	2.003E-11
34	Human Diseases	Infectious disease: bacterial	fca05132	Salmonella infection	19/96	255/10177	19	1.951E-12	2.054E-11	2.054E-11
35	Human Diseases	Cancer: specific types	fca05219	Bladder cancer	10/96	42/10177	10	3.965E-12	4.055E-11	4.055E-11
36	Human Diseases	Cardiovascular disease	fca05418	Fluid shear stress and atherosclerosis	15/96	149/10177	15	7.077E-12	7.037E-11	7.037E-11

No.	Category	Subcategory	KEGG ID	Pathway	GeneRatio	BgRatio	Count	P value	Adjusted P	q value
37	Environmental Information Processing	Signal transduction	fca04064	NF-kappa B signaling pathway	13/96	104/10177	13	1.232E-11	1.192E-10	1.192E-10
38	Human Diseases	Infectious disease: viral	fca05171	Coronavirus disease	20/96	329/10177	20	2.029E-11	1.912E-10	1.912E-10
39	Human Diseases	Infectious disease: parasitic	fca05143	African trypanosomiasis	9/96	35/10177	9	2.328E-11	2.137E-10	2.137E-10
40	Human Diseases	Infectious disease: bacterial	fca05135	Yersinia infection	14/96	137/10177	14	3.066E-11	2.744E-10	2.744E-10
41	Environmental Information Processing	Signal transduction	fca04668	TNF signaling pathway	13/96	115/10177	13	4.523E-11	3.949E-10	3.949E-10
42	Human Diseases	Drug resistance: antineoplastic	fca01522	Endocrine resistance	12/96	96/10177	12	8.001E-11	6.820E-10	6.820E-10
43	Human Diseases	Cancer: overview	fca05207	Chemical carcinogenesis - receptor activation	15/96	180/10177	15	1.085E-10	9.030E-10	9.030E-10
44	Human Diseases	Infectious disease: viral	fca05165	Human papillomavirus infection	19/96	328/10177	19	1.591E-10	1.294E-09	1.294E-09
45	Human Diseases	Cancer: specific types	fca05210	Colorectal cancer	11/96	90/10177	11	6.605E-10	5.255E-09	5.255E-09
46	Human Diseases	Cancer: specific types	fca05224	Breast cancer	13/96	146/10177	13	9.263E-10	7.209E-09	7.209E-09
47	Cellular Processes	Cell growth and death	fca04217	Necroptosis	14/96	177/10177	14	9.572E-10	7.265E-09	7.265E-09
48	Organismal Systems	Immune system	fca04623	Cytosolic D-sensing pathway	10/96	71/10177	10	9.943E-10	7.265E-09	7.265E-09
49	Organismal Systems	Endocrine system	fca04917	Prolactin signaling pathway	10/96	71/10177	10	9.943E-10	7.265E-09	7.265E-09
50	Human Diseases	Cancer: specific types	fca05220	Chronic myeloid leukemia	10/96	76/10177	10	1.978E-09	1.416E-08	1.416E-08
51	Organismal Systems	Endocrine system	fca04926	Relaxin signaling pathway	12/96	129/10177	12	2.600E-09	1.825E-08	1.825E-08
52	Cellular Processes	Transport and catabolism	fca04148	Efferocytosis	13/96	159/10177	13	2.662E-09	1.832E-08	1.832E-08
53	Cellular Processes	Cell growth and death	fca04218	Cellular senescence	13/96	162/10177	13	3.349E-09	2.262E-08	2.262E-08
54	Environmental Information Processing	Signal transduction	fca04630	JAK-STAT signaling pathway	13/96	164/10177	13	3.892E-09	2.580E-08	2.580E-08
55	Human Diseases	Endocrine and metabolic disease	fca04932	Non-alcoholic fatty liver disease	13/96	167/10177	13	4.858E-09	3.162E-08	3.162E-08
56	Human Diseases	Infectious disease: viral	fca05170	Human immunodeficiency virus 1 infection	14/96	213/10177	14	1.064E-08	6.740E-08	6.740E-08
57	Human Diseases	Cancer: specific types	fca05226	Gastric cancer	12/96	146/10177	12	1.073E-08	6.740E-08	6.740E-08
58	Organismal Systems	Immune system	fca04658	Th1 and Th2 cell differentiation	10/96	91/10177	10	1.186E-08	7.319E-08	7.319E-08
59	Human Diseases	Cancer: specific types	fca05222	Small cell lung cancer	10/96	95/10177	10	1.808E-08	1.097E-07	1.097E-07
60	Human Diseases	Infectious disease: viral	fca05166	Human T-cell leukemia virus 1 infection	14/96	226/10177	14	2.265E-08	1.352E-07	1.352E-07
61	Human Diseases	Drug resistance: antineoplastic	fca01524	Platinum drug resistance	9/96	74/10177	9	2.696E-08	1.560E-07	1.560E-07
62	Human Diseases	Immune disease	fca05323	Rheumatoid arthritis	10/96	99/10177	10	2.702E-08	1.560E-07	1.560E-07
63	Human Diseases	Cancer: specific types	fca05225	Hepatocellular carcinoma	12/96	163/10177	12	3.710E-08	2.108E-07	2.108E-07
64	Environmental Information Processing	Signal transduction	fca04068	FoxO signaling pathway	11/96	132/10177	11	3.973E-08	2.223E-07	2.223E-07
65	Human Diseases	Drug resistance: antineoplastic	fca01521	EGFR tyrosine kinase inhibitor resistance	9/96	80/10177	9	5.400E-08	2.974E-07	2.974E-07
66	Human Diseases	Endocrine and metabolic disease	fca04931	Insulin resistance	10/96	108/10177	10	6.264E-08	3.398E-07	3.398E-07
67	Human Diseases	Cancer: overview	fca05203	Viral carcinogenesis	13/96	210/10177	13	7.587E-08	4.054E-07	4.054E-07
68	Cellular Processes	Cell growth and death	fca04210	Apoptosis	11/96	144/10177	11	9.783E-08	5.150E-07	5.150E-07
69	Organismal Systems	Endocrine system	fca04919	Thyroid hormone signaling pathway	10/96	120/10177	10	1.711E-07	8.878E-07	8.878E-07
70	Organismal Systems	Immune system	fca04660	T cell receptor signaling pathway	10/96	122/10177	10	2.001E-07	1.023E-06	1.023E-06
71	Human Diseases	Cancer: overview	fca05230	Central carbon metabolism in cancer	8/96	70/10177	8	2.725E-07	1.374E-06	1.374E-06
72	Organismal Systems	Endocrine system	fca04915	Estrogen signaling pathway	10/96	137/10177	10	5.927E-07	2.947E-06	2.947E-06
73	Environmental Information Processing	Signal transduction	fca04010	MAPK signaling pathway	14/96	300/10177	14	7.512E-07	3.684E-06	3.684E-06
74	Human Diseases	Neurodegenerative disease	fca05010	Alzheimer disease	16/96	400/10177	16	8.901E-07	4.306E-06	4.306E-06
75	Human Diseases	Cancer: specific types	fca05216	Thyroid cancer	6/96	36/10177	6	9.329E-07	4.453E-06	4.453E-06
76	Organismal Systems	Endocrine system	fca04928	Parathyroid hormone synthesis, secretion and action	9/96	115/10177	9	1.249E-06	5.884E-06	5.884E-06
77	Organismal Systems	Immune system	fca04062	Chemokine signaling pathway	11/96	187/10177	11	1.362E-06	6.333E-06	6.333E-06
78	Human Diseases	Cancer: overview	fca05208	Chemical carcinogenesis - reactive oxygen species	12/96	237/10177	12	2.135E-06	9.798E-06	9.798E-06
79	Organismal Systems	Nervous system	fca04722	Neurotrophin signaling pathway	9/96	123/10177	9	2.197E-06	9.957E-06	9.957E-06
80	Organismal Systems	Immune system	fca04613	Neutrophil extracellular trap formation	11/96	201/10177	11	2.763E-06	1.224E-05	1.224E-05
81	Human Diseases	Immune disease	fca05332	Graft-versus-host disease	6/96	43/10177	6	2.769E-06	1.224E-05	1.224E-05
82	Human Diseases	Neurodegenerative disease	fca05022	Pathways of neurodegeneration - multiple diseases	17/96	491/10177	17	2.850E-06	1.244E-05	1.244E-05
83	Human Diseases	Cancer: specific types	fca05218	Melanoma	7/96	71/10177	7	4.332E-06	1.846E-05	1.846E-05

No.	Category	Subcategory	KEGG ID	Pathway	GeneRatio	BgRatio	Count	P value	Adjusted P	q value
84	Human Diseases	Cancer: specific types	fca05223	Non-small cell lung cancer	7/96	71/10177	7	4.332E-06	1.846E-05	1.846E-05
85	Organismal Systems	Endocrine system	fca04920	Adipocytokine signaling pathway	7/96	73/10177	7	5.226E-06	2.201E-05	2.201E-05
86	Human Diseases	Cancer: specific types	fca05214	Glioma	7/96	75/10177	7	6.270E-06	2.610E-05	2.610E-05
87	Human Diseases	Drug resistance: antineoplastic	fca01523	Antifolate resistance	5/96	29/10177	5	6.676E-06	2.747E-05	2.747E-05
88	Organismal Systems	Immune system	fca04662	B cell receptor signaling pathway	7/96	78/10177	7	8.156E-06	3.318E-05	3.318E-05
89	Environmental Information Processing	Signaling molecules and interaction	fca04060	Cytokine-cytokine receptor interaction	12/96	281/10177	12	1.226E-05	4.933E-05	4.933E-05
90	Human Diseases	Cancer: specific types	fca05213	Endometrial cancer	6/96	57/10177	6	1.482E-05	5.895E-05	5.895E-05
91	Environmental Information Processing	Signal transduction	fca04071	Sphingolipid signaling pathway	8/96	126/10177	8	2.354E-05	9.262E-05	9.262E-05
92	Cellular Processes	Cellular community - eukaryotes	fca04520	Adherens junction	7/96	93/10177	7	2.607E-05	1.015E-04	1.015E-04
93	Human Diseases	Immune disease	fca05330	Allograft rejection	5/96	39/10177	5	3.004E-05	1.156E-04	1.156E-04
94	Human Diseases	Cancer: specific types	fca05221	Acute myeloid leukemia	6/96	67/10177	6	3.778E-05	1.439E-04	1.439E-04
95	Human Diseases	Cardiovascular disease	fca05415	Diabetic cardiomyopathy	10/96	219/10177	10	3.889E-05	1.466E-04	1.466E-04
96	Human Diseases	Cancer: overview	fca05202	Transcriptional misregulation in cancer	9/96	183/10177	9	5.453E-05	2.034E-04	2.034E-04
97	Organismal Systems	Immune system	fca04622	RIG-I-like receptor signaling pathway	6/96	72/10177	6	5.695E-05	2.087E-04	2.087E-04
98	Organismal Systems	Immune system	fca04650	Natural killer cell mediated cytotoxicity	7/96	105/10177	7	5.714E-05	2.087E-04	2.087E-04
99	Cellular Processes	Cellular community - eukaryotes	fca04550	Signaling pathways regulating pluripotency of stem cells	8/96	143/10177	8	5.843E-05	2.113E-04	2.113E-04
100	Human Diseases	Endocrine and metabolic disease	fca04940	Type I diabetes mellitus	5/96	45/10177	5	6.096E-05	2.182E-04	2.182E-04
101	Human Diseases	Endocrine and metabolic disease	fca04930	Type II diabetes mellitus	5/96	47/10177	5	7.541E-05	2.673E-04	2.673E-04
102	Environmental Information Processing	Signal transduction	fca04350	TGF-beta signaling pathway	7/96	111/10177	7	8.144E-05	2.858E-04	2.858E-04
103	Cellular Processes	Cell growth and death	fca04115	p53 signaling pathway	6/96	78/10177	6	8.946E-05	3.110E-04	3.110E-04
104	Cellular Processes	Cellular community - eukaryotes	fca04510	Focal adhesion	9/96	202/10177	9	1.167E-04	4.019E-04	4.019E-04
105	Organismal Systems	Immune system	fca04611	Platelet activation	7/96	122/10177	7	1.477E-04	5.034E-04	5.034E-04
106	Organismal Systems	Aging	fca04211	Longevity regulating pathway	6/96	88/10177	6	1.750E-04	5.911E-04	5.911E-04
107	Organismal Systems	Immune system	fca04666	Fc gamma R-mediated phagosome formation	6/96	93/10177	6	2.370E-04	7.857E-04	7.857E-04
108	Organismal Systems	Endocrine system	fca04912	GnRH signaling pathway	6/96	93/10177	6	2.370E-04	7.857E-04	7.857E-04
109	Organismal Systems	Immune system	fca04664	Fc epsilon RI signaling pathway	5/96	68/10177	5	4.384E-04	0.00144	0.00144
110	Organismal Systems	Endocrine system	fca04921	Oxytocin signaling pathway	7/96	151/10177	7	5.448E-04	0.00177297	0.00177297
111	Human Diseases	Cancer: specific types	fca05211	Renal cell carcinoma	5/96	72/10177	5	5.713E-04	0.00184252	0.00184252
112	Human Diseases	Endocrine and metabolic disease	fca04934	Cushing syndrome	7/96	154/10177	7	6.128E-04	0.00195863	0.00195863
113	Cellular Processes	Transport and catabolism	fca04145	Phagocytosis	9/96	254/10177	9	6.374E-04	0.0020193	0.0020193
114	Environmental Information Processing	Signaling molecules and interaction	fca04518	Integrin signaling	7/96	158/10177	7	7.137E-04	0.00224136	0.00224136
115	Organismal Systems	Endocrine system	fca04935	Growth hormone synthesis, secretion and action	6/96	118/10177	6	8.485E-04	0.00264135	0.00264135
116	Organismal Systems	Immune system	fca04672	Intestinal immune network for IgA production	4/96	46/10177	4	8.959E-04	0.00276483	0.00276483
117	Genetic Information Processing	Information processing in viruses	fca03272	Virion - Hepatitis viruses	4/96	49/10177	4	0.00113836	0.00348319	0.00348319
118	Environmental Information Processing	Signal transduction	fca04012	ErbB signaling pathway	5/96	84/10177	5	0.00115337	0.0034992	0.0034992
119	Cellular Processes	Transport and catabolism	fca04140	Autophagy - animal	7/96	179/10177	7	0.00148032	0.00445341	0.00445341
120	Environmental Information Processing	Signaling molecules and interaction	fca04061	Viral protein interaction with cytokine and cytokine receptor	5/96	89/10177	5	0.00149443	0.00445839	0.00445839
121	Environmental Information Processing	Signal transduction	fca04371	Apelin signaling pathway	6/96	141/10177	6	0.00212201	0.00627065	0.00627065
122	Environmental Information Processing	Signal transduction	fca04370	VEGF signaling pathway	4/96	58/10177	4	0.00213693	0.00627065	0.00627065
123	Human Diseases	Cancer: overview	fca05231	Choline metabolism in cancer	5/96	97/10177	5	0.00218718	0.00636594	0.00636594
124	Human Diseases	Cardiovascular disease	fca05410	Hypertrophic cardiomyopathy	5/96	100/10177	5	0.00249919	0.00721539	0.00721539
125	Environmental Information Processing	Signal transduction	fca04072	Phospholipase D signaling pathway	6/96	153/10177	6	0.00319265	0.00914375	0.00914375
126	Environmental Information Processing	Signal transduction	fca04015	Rap1 signaling pathway	7/96	214/10177	7	0.00403854	0.0114746	0.0114746
127	Organismal Systems	Nervous system	fca04725	Cholinergic synapse	5/96	112/10177	5	0.0040764	0.011491	0.011491

No.	Category	Subcategory	KEGG ID	Pathway	GeneRatio	BgRatio	Count	P value	Adjusted P	q value
128	Environmental Information Processing	Signaling molecules and interaction	fca04081	Hormone signaling	7/96	218/10177	7	0.00446821	0.012497	0.012497
129	Cellular Processes	Cell growth and death	fca04215	Apoptosis - multiple species	3/96	36/10177	3	0.0046357	0.012865	0.012865
130	Organismal Systems	Immune system	fca04670	Leukocyte transendothelial migration	5/96	117/10177	5	0.00490729	0.0135139	0.0135139
131	Metabolism	Metabolism of cofactors and vitamins	fca00760	Nicotinate and nicotinamide metabolism	3/96	38/10177	3	0.00540329	0.0146547	0.0146547
132	Human Diseases	Neurodegenerative disease	fca05020	Prion disease	8/96	285/10177	8	0.00540342	0.0146547	0.0146547
133	Environmental Information Processing	Signal transduction	fca04014	Ras signaling pathway	7/96	235/10177	7	0.00669122	0.0180109	0.0180109
134	Cellular Processes	Cellular community - eukaryotes	fca04540	Gap junction	4/96	86/10177	4	0.00875715	0.023396	0.023396
135	Environmental Information Processing	Signaling molecules and interaction	fca04519	Cadherin signaling	9/96	384/10177	9	0.010142	0.026895	0.026895
136	Human Diseases	Immune disease	fca05320	Autoimmune thyroid disease	3/96	48/10177	3	0.0103529	0.0272526	0.0272526
137	Organismal Systems	Immune system	fca04640	Hematopoietic cell lineage	4/96	97/10177	4	0.0132143	0.0345309	0.0345309
138	Metabolism	Amino acid metabolism	fca00220	Arginine biosynthesis	2/96	20/10177	2	0.0149813	0.0388644	0.0388644
139	Human Diseases	Cardiovascular disease	fca05414	Dilated cardiomyopathy	4/96	104/10177	4	0.0166879	0.0429805	0.0429805
140	Cellular Processes	Cell growth and death	fca04110	Cell cycle	5/96	161/10177	5	0.0179671	0.045876	0.045876
141	Organismal Systems	Nervous system	fca04730	Long-term depression	3/96	59/10177	3	0.0180685	0.045876	0.045876
142	Environmental Information Processing	Signaling molecules and interaction	fca04517	IgSF CAM signaling	7/96	293/10177	7	0.0205866	0.0514281	0.0514281
143	Human Diseases	Cancer: specific types	fca05217	Basal cell carcinoma	3/96	62/10177	3	0.0206009	0.0514281	0.0514281
144	Organismal Systems	Nervous system	fca04726	Serotonergic synapse	4/96	111/10177	4	0.0206862	0.0514281	0.0514281
145	Organismal Systems	Endocrine system	fca04929	GnRH secretion	3/96	64/10177	3	0.0223921	0.0552853	0.0552853
146	Human Diseases	Immune disease	fca05310	Asthma	2/96	28/10177	2	0.0283891	0.0696118	0.0696118
147	Human Diseases	Cardiovascular disease	fca05416	Viral myocarditis	3/96	73/10177	3	0.0314722	0.0766465	0.0766465
148	Organismal Systems	Environmental adaptation	fca04710	Circadian rhythm	2/96	34/10177	2	0.0406308	0.0982826	0.0982826
149	Organismal Systems	Excretory system	fca04960	Aldosterone-regulated sodium reabsorption	2/96	37/10177	2	0.0473707	0.113817	0.113817
150	Human Diseases	Cardiovascular disease	fca05412	Arrhythmogenic right ventricular cardiomyopathy	3/96	87/10177	3	0.0488602	0.116613	0.116613
151	Human Diseases	Immune disease	fca05322	Systemic lupus erythematosus	4/96	148/10177	4	0.0511152	0.121187	0.121187
152	Environmental Information Processing	Signaling molecules and interaction	fca04512	ECM-receptor interaction	3/96	91/10177	3	0.0545345	0.128443	0.128443
153	Organismal Systems	Circulatory system	fca04261	Adrenergic signaling in cardiomyocytes	4/96	152/10177	4	0.0553638	0.129544	0.129544
154	Organismal Systems	Digestive system	fca04975	Fat digestion and absorption	2/96	41/10177	2	0.0569378	0.132362	0.132362
155	Environmental Information Processing	Signal transduction	fca04024	cAMP signaling pathway	5/96	225/10177	5	0.0613508	0.141701	0.141701
156	Environmental Information Processing	Signal transduction	fca04150	mTOR signaling pathway	4/96	158/10177	4	0.0620852	0.142478	0.142478
157	Genetic Information Processing	Replication and repair	fca03410	Base excision repair	2/96	45/10177	2	0.0671129	0.153035	0.153035
158	Organismal Systems	Digestive system	fca04974	Protein digestion and absorption	3/96	100/10177	3	0.0683939	0.154969	0.154969
159	Cellular Processes	Cell motility	fca04810	Regulation of actin cytoskeleton	5/96	233/10177	5	0.06909	0.155561	0.155561
160	Organismal Systems	Endocrine system	fca04916	Melanogenesis	3/96	101/10177	3	0.0700243	0.156679	0.156679
161	Human Diseases	Neurodegenerative disease	fca05014	Amyotrophic lateral sclerosis	7/96	385/10177	7	0.0711266	0.158157	0.158157
162	Cellular Processes	Transport and catabolism	fca04137	Mitophagy - animal	3/96	102/10177	3	0.0716722	0.158387	0.158387
163	Environmental Information Processing	Membrane transport	fca02010	ABC transporters	2/96	47/10177	2	0.0724103	0.159036	0.159036
164	Metabolism	Metabolism of cofactors and vitamins	fca00740	Riboflavin metabolism	1/96	8/10177	1	0.0730435	0.159449	0.159449
165	Organismal Systems	Endocrine system	fca04913	Ovarian steroidogenesis	2/96	48/10177	2	0.0751085	0.161981	0.161981
166	Human Diseases	Substance dependence	fca05030	Cocaine addiction	2/96	48/10177	2	0.0751085	0.161981	0.161981
167	Genetic Information Processing	Information processing in viruses	fca03266	Virion - Herpesvirus	1/96	9/10177	1	0.0817944	0.175344	0.175344
168	Environmental Information Processing	Signal transduction	fca04310	Wnt signaling pathway	4/96	175/10177	4	0.0833494	0.177614	0.177614
169	Metabolism	Amino acid metabolism	fca00330	Arginine and proline metabolism	2/96	52/10177	2	0.0862112	0.181551	0.181551
170	Organismal Systems	Excretory system	fca04961	Endocrine and other factor-regulated calcium reabsorption	2/96	52/10177	2	0.0862112	0.181551	0.181551
171	Organismal Systems	Digestive system	fca04979	Cholesterol metabolism	2/96	53/10177	2	0.0890604	0.186454	0.186454

No.	Category	Subcategory	KEGG ID	Pathway	GeneRatio	BgRatio	Count	P value	Adjusted P	q value
172	Environmental Information Processing	Signal transduction	fca04020	Calcium signaling pathway	5/96	256/10177	5	0.0942105	0.196089	0.196089
173	Cellular Processes	Cell growth and death	fca04216	Ferroptosis	2/96	55/10177	2	0.0948415	0.196262	0.196262

Table S2G. Complete KEGG enrichment results: enrichment metrics and contributing genes (linked to Table S2F by row number/KEGG ID).

No.	KEGG ID	Rich factor	Fold enrichment	z score	Contributing genes
1	fca05161	0.154321	16.3596	19.2303	TP53; TLR4; E2F2; JAK1; NFKBIA; MAPK1; MMP9; STAT3; CXCL8; CASP3; TNF; IL6; TLR2; RELA; MAPK3; NFKB1; JAK2; CDKN1A; TGFB1; MYD88; STAT1; CASP8; BCL2; FASLG; SMAD3
2	fca04659	0.201835	21.3966	20.8916	JAK1; NFKBIA; MTOR; MAPK1; STAT3; IFNG; HIF1A; RORA; IL6; IL1B; IL17A; FOXP3; RELA; MAPK3; NFKB1; JAK2; IL2; TGFB1; RORC; STAT1; AHR; SMAD3
3	fca05417	0.128571	13.6299	18.0468	NLRP3; TP53; TLR4; PPARG; NFKBIA; XBP1; MAPK1; MMP9; STAT3; CXCL8; NOS3; CASP3; TNF; IL6; IL1B; TLR2; LYN; RELA; MAPK3; NFKB1; JAK2; ABCA1; NFE2L2; MYD88; CASP8; BCL2; FASLG
4	fca05321	0.28125	29.8154	22.5656	TLR4; STAT3; IFNG; RORA; TNF; IL6; IL1B; TLR2; IL17A; FOXP3; RELA; NFKB1; IL2; TGFB1; RORC; STAT1; IL10; SMAD3
5	fca05140	0.24	25.4425	20.7322	TLR4; JAK1; NFKBIA; MAPK1; IFNG; TNF; IL1B; TLR2; RELA; MAPK3; NOS2; NFKB1; JAK2; PTGS2; TGFB1; MYD88; STAT1; IL10
6	fca05142	0.186916	19.815	19.0921	TLR4; NFKBIA; MAPK1; CXCL8; TLR9; IFNG; TNF; IL6; IL1B; TLR2; RELA; MAPK3; NOS2; NFKB1; IL2; TGFB1; MYD88; CASP8; IL10; FASLG
7	fca05145	0.178571	18.9304	18.6194	TLR4; JAK1; NFKBIA; MAPK1; STAT3; IFNG; CASP3; TNF; TLR2; RELA; MAPK3; NOS2; NFKB1; JAK2; TGFB1; MYD88; STAT1; CASP8; BCL2; IL10
8	fca05152	0.132948	14.0939	16.9503	TLR4; JAK1; MAPK1; SYK; TLR9; IFNG; CASP3; TNF; IL6; IL1B; TLR2; RELA; MAPK3; NOS2; VDR; NFKB1; JAK2; TGFB1; MYD88; STAT1; CASP8; BCL2; IL10
9	fca04933	0.186275	19.747	18.5687	PRKCD; MAPK1; STAT3; CXCL8; NOS3; CASP3; TNF; IL6; IL1B; COL1A1; RELA; MAPK3; NFKB1; JAK2; TGFB1; MMP2; STAT1; BCL2; SMAD3
10	fca05205	0.109524	11.6107	15.1615	TP53; TLR4; CAV1; EGFR; MTOR; FGFR1; MAPK1; MMP9; STAT3; HIF1A; CTNNB1; ITGB3; CASP3; TNF; TLR2; COL1A1; MAPK3; CDKN1A; TGFB1; MMP2; THBS1; FASLG; ESR1
11	fca05164	0.128834	13.6578	15.8972	NLRP3; TLR4; JAK1; NFKBIA; MAPK1; CXCL8; IFNG; PLG; CASP3; TNF; IL6; IL1B; RELA; MAPK3; NFKB1; JAK2; IL33; MYD88; STAT1; CASP8; FASLG
12	fca05235	0.188889	20.0242	17.6896	TLR4; EGFR; JAK1; NFKBIA; MTOR; MAPK1; STAT3; TLR9; IFNG; HIF1A; TLR2; RELA; MAPK3; NFKB1; JAK2; MYD88; STAT1
13	fca05167	0.110553	11.7197	14.9026	TP53; E2F2; JAK1; NFKBIA; MTOR; MAPK1; STAT3; CXCL8; SYK; HIF1A; CTNNB1; CASP3; IL6; LYN; RELA; MAPK3; NFKB1; JAK2; PTGS2; CDKN1A; STAT1; CASP8
14	fca05160	0.129032	13.6788	15.5217	TP53; E2F2; EGFR; JAK1; NFKBIA; MAPK1; CLDN2; STAT3; IFNG; CTNNB1; CASP3; TNF; RELA; MAPK3; OCLN; NFKB1; CDKN1A; STAT1; CASP8; FASLG
15	fca05168	0.117318	12.437	15.0645	TP53; JAK1; NFKBIA; MTOR; SYK; TLR9; IFNG; ITGB3; CASP3; TNF; IL6; IL1B; TLR2; RELA; NFKB1; JAK2; MYD88; STAT1; CASP8; BCL2; FASLG
16	fca05163	0.0973451	10.3196	13.8258	TP53; E2F2; EGFR; JAK1; NFKBIA; MTOR; MAPK1; STAT3; CXCL8; CTNNB1; ITGB3; CASP3; TNF; IL6; IL1B; RELA; MAPK3; NFKB1; PTGS2; CDKN1A; CASP8; FASLG
17	fca05162	0.136364	14.456	15.1845	TP53; TLR4; JAK1; NFKBIA; STAT3; TLR9; CASP3; IL6; IL1B; TLR2; RELA; NFKB1; IL2; MYD88; STAT1; CASP8; BCL2; FASLG
18	fca05206	0.118012	12.5105	14.3659	TP53; E2F2; EGFR; MTOR; SIRT1; MAPK1; MMP9; HMOX1; STAT3; ITGB3; CASP3; MAPK3; BRCA1; ABCB1; NFKB1; PTGS2; CDKN1A; THBS1; BCL2
19	fca04657	0.163265	17.3078	15.8297	NFKBIA; MAPK1; MMP9; CXCL8; IFNG; CASP3; TNF; IL6; IL1B; IL17A; RELA; MAPK3; NFKB1; S100A8; PTGS2; CASP8
20	fca04620	0.158416	16.7937	15.5659	TLR4; JAK1; NFKBIA; MAPK1; CXCL8; TLR9; TNF; IL6; IL1B; TLR2; RELA; MAPK3; NFKB1; MYD88; STAT1; CASP8
21	fca04625	0.153846	16.3093	15.3132	PRKCD; NLRP3; NFKBIA; MAPK1; SYK; TNF; IL6; IL1B; RELA; MAPK3; NFKB1; PTGS2; IL2; STAT1; CASP8; IL10
22	fca05133	0.184211	19.5282	15.8209	NLRP3; TLR4; MAPK1; CXCL8; CASP3; TNF; IL6; IL1B; RELA; MAPK3; NOS2; NFKB1; MYD88; IL10
23	fca05212	0.181818	19.2746	15.7074	TP53; E2F2; EGFR; JAK1; MTOR; MAPK1; STAT3; RELA; MAPK3; NFKB1; CDKN1A; TGFB1; STAT1; SMAD3
24	fca04066	0.137931	14.6221	14.3988	TLR4; EGFR; MTOR; MAPK1; HMOX1; STAT3; NOS3; IFNG; HIF1A; IL6; RELA; MAPK3; NOS2; NFKB1; CDKN1A; BCL2
25	fca05134	0.2	21.2021	15.9443	CLK1; TLR4; NFKBIA; CXCL8; CASP3; TNF; IL6; IL1B; TLR2; RELA; NFKB1; MYD88; CASP8
26	fca05144	0.235294	24.9436	16.7274	TLR4; CXCL8; TLR9; IFNG; TNF; IL6; IL1B; TLR2; TGFB1; THBS1; MYD88; IL10
27	fca04151	0.0649718	6.88768	11.0026	TP53; TLR4; EGFR; JAK1; MTOR; FGFR1; MAPK1; SYK; NOS3; ITGB3; IL6; TLR2; COL1A1; RELA; MAPK3; BRCA1; NFKB1; JAK2; CDKN1A; IL2; THBS1; BCL2; FASLG
28	fca04621	0.104294	11.0563	12.6299	PRKCD; NLRP3; TLR4; JAK1; NFKBIA; MAPK1; CXCL8; TNF; IL6; IL1B; RELA; MAPK3; NFKB1; MYD88; STAT1; CASP8; BCL2
29	fca05169	0.0895522	9.49347	11.868	TP53; E2F2; JAK1; NFKBIA; STAT3; SYK; CASP3; TNF; IL6; TLR2; LYN; RELA; NFKB1; CDKN1A; MYD88; STAT1; CASP8; BCL2
30	fca05215	0.135922	14.4092	13.3473	TP53; E2F2; EGFR; NFKBIA; MTOR; FGFR1; MAPK1; MMP9; CTNNB1; RELA; MAPK3; NFKB1; CDKN1A; BCL2
31	fca05146	0.134615	14.2706	13.274	TLR4; CXCL8; IFNG; CASP3; TNF; IL6; IL1B; TLR2; COL1A1; RELA; NOS2; NFKB1; TGFB1; IL10
32	fca04380	0.11811	12.5209	12.7491	PPARG; JAK1; NFKBIA; MAPK1; SYK; IFNG; ITGB3; TNF; IL1B; RELA; MAPK3; TNFSF11; NFKB1; TGFB1; STAT1
33	fca04936	0.110294	11.6923	12.2497	TLR4; NFKBIA; SIRT1; CXCL8; CTNNB1; CASP3; TNF; IL6; IL1B; IL17A; RELA; NFKB1; MYD88; CASP8; FASLG
34	fca05132	0.0745098	7.89882	10.8872	NLRP3; PTPRC; TLR4; NFKBIA; MAPK1; CXCL8; TLR9; CTNNB1; CASP3; TNF; IL6; IL1B; TLR2; RELA; MAPK3; NFKB1; MYD88; CASP8; BCL2
35	fca05219	0.238095	25.2406	15.3613	TP53; E2F2; EGFR; MAPK1; MMP9; CXCL8; MAPK3; CDKN1A; MMP2; THBS1
36	fca05418	0.100671	10.6722	11.606	TP53; CAV1; MMP9; HMOX1; NOS3; IFNG; CTNNB1; ITGB3; TNF; IL1B; RELA; NFKB1; NFE2L2; MMP2; BCL2
37	fca04064	0.125	13.2513	12.2544	TLR4; NFKBIA; CXCL8; SYK; TNF; IL1B; LYN; RELA; TNFSF11; NFKB1; PTGS2; MYD88; BCL2
38	fca05171	0.0607903	6.4444	9.79595	NLRP3; TLR4; ACE2; EGFR; JAK1; NFKBIA; MAPK1; STAT3; CXCL8; SYK; TNF; IL6; IL1B; TLR2; RELA; MAPK3; NFKB1; IL2; MYD88; STAT1
39	fca05143	0.257143	27.2598	15.1858	TLR9; IFNG; TNF; IL6; IL1B; IDO1; MYD88; IL10; FASLG
40	fca05135	0.10219	10.8332	11.3073	NLRP3; TLR4; NFKBIA; MAPK1; CXCL8; TNF; IL6; IL1B; RELA; MAPK3; NFKB1; IL2; MYD88; IL10
41	fca04668	0.113043	11.9838	11.5593	NFKBIA; MAPK1; MMP9; CASP3; TNF; IL6; IL1B; RELA; MAPK3; NFKB1; PTGS2; MMP14; CASP8
42	fca01522	0.125	13.2513	11.769	TP53; E2F2; EGFR; MTOR; MAPK1; MMP9; ESR2; MAPK3; CDKN1A; MMP2; BCL2; ESR1
43	fca05207	0.0833333	8.8342	10.3483	EGFR; MTOR; MAPK1; STAT3; ESR2; RELA; MAPK3; TNFSF11; VDR; NFKB1; JAK2; CHRNA7; BCL2; ESR1; AHR

No.	KEGG ID	Rich factor	Fold enrichment	z score	Contributing genes
44	fca05165	0.0579268	6.14085	9.23523	TP53; EGFR; JAK1; MTOR; MAPK1; CTNNB1; ITGB3; CASP3; TNF; COL1A1; RELA; MAPK3; NFKB1; PTGS2; CDKN1A; THBS1; STAT1; CASP8; FASLG
45	fca05210	0.122222	12.9568	11.118	TP53; EGFR; MTOR; MAPK1; CTNNB1; CASP3; MAPK3; CDKN1A; TGFB1; BCL2; SMAD3
46	fca05224	0.0890411	9.43928	10.0226	TP53; E2F2; EGFR; MTOR; FGFR1; MAPK1; CTNNB1; ESR2; MAPK3; BRCA1; TNFSF11; CDKN1A; ESR1
47	fca04217	0.079096	8.385	9.67185	NLRP3; TLR4; JAK1; STAT3; IFNG; HMGB1; TNF; IL1B; JAK2; IL33; STAT1; CASP8; BCL2; FASLG
48	fca04623	0.140845	14.931	11.4946	NLRP3; NFKBIA; CASP3; IL6; IL1B; RELA; NFKB1; IL33; POLR3C; CASP8
49	fca04917	0.140845	14.931	11.4946	MAPK1; STAT3; ESR2; RELA; MAPK3; TNFSF11; NFKB1; JAK2; STAT1; ESR1
50	fca05220	0.131579	13.9487	11.0567	TP53; E2F2; NFKBIA; MAPK1; RELA; MAPK3; NFKB1; CDKN1A; TGFB1; SMAD3
51	fca04926	0.0930233	9.86143	9.88397	EGFR; NFKBIA; MAPK1; MMP9; NOS3; COL1A1; RELA; MAPK3; NOS2; NFKB1; TGFB1; MMP2
52	fca04148	0.081761	8.66752	9.50901	PPARG; SIRT1; MAPK1; HIF1A; ITGB3; CASP3; MAPK3; JAK2; ABCA1; PTGS2; TGFB1; THBS1; IL10
53	fca04218	0.0802469	8.50701	9.39878	TP53; E2F2; MTOR; SIRT1; MAPK1; CXCL8; IL6; RELA; MAPK3; NFKB1; CDKN1A; TGFB1; SMAD3
54	fca04630	0.0792683	8.40326	9.32686	EGFR; JAK1; MTOR; STAT3; IFNG; IL6; JAK2; CDKN1A; IL2; STAT1; BCL2; IL10; PTPN2
55	fca04932	0.0778443	8.25231	9.22125	PPARG; XBP1; CXCL8; CASP3; TNF; IL6; IL1B; RELA; MLXIPL; NFKB1; TGFB1; CASP8; FASLG
56	fca05170	0.0657277	6.96782	8.58937	TLR4; NFKBIA; MTOR; MAPK1; CASP3; TNF; TLR2; RELA; MAPK3; NFKB1; MYD88; CASP8; BCL2; FASLG
57	fca05226	0.0821918	8.71318	9.16032	TP53; E2F2; EGFR; MTOR; MAPK1; CTNNB1; MAPK3; ABCB1; CDKN1A; TGFB1; BCL2; SMAD3
58	fca04658	0.10989	11.6495	9.95778	JAK1; NFKBIA; MAPK1; IFNG; RELA; MAPK3; NFKB1; JAK2; IL2; STAT1
59	fca05222	0.105263	11.159	9.70759	TP53; E2F2; NFKBIA; CASP3; RELA; NOS2; NFKB1; PTGS2; CDKN1A; BCL2
60	fca05166	0.0619469	6.56702	8.25878	TP53; E2F2; JAK1; NFKBIA; MAPK1; TNF; IL6; RELA; MAPK3; NFKB1; CDKN1A; IL2; TGFB1; SMAD3
61	fca01524	0.121622	12.8932	10.0198	TP53; MAPK1; CASP3; MAPK3; BRCA1; CDKN1A; CASP8; BCL2; FASLG
62	fca05323	0.10101	10.7081	9.47192	TLR4; CXCL8; IFNG; TNF; IL6; IL1B; TLR2; IL17A; TNFSF11; TGFB1
63	fca05225	0.0736196	7.80445	8.54585	TP53; E2F2; EGFR; MTOR; MAPK1; HMOX1; CTNNB1; MAPK3; NFE2L2; CDKN1A; TGFB1; SMAD3
64	fca04068	0.0833333	8.8342	8.84055	EGFR; SIRT1; MAPK1; STAT3; IL6; MAPK3; CDKN1A; TGFB1; IL10; FASLG; SMAD3
65	fca01521	0.1125	11.9262	9.57391	EGFR; JAK1; MTOR; MAPK1; STAT3; IL6; MAPK3; JAK2; BCL2
66	fca04931	0.0925926	9.81578	8.98777	PRKCD; NFKBIA; MTOR; STAT3; NOS3; TNF; IL6; RELA; MLXIPL; NFKB1
67	fca05203	0.0619048	6.56255	7.94829	TP53; JAK1; NFKBIA; MAPK1; STAT3; SYK; CASP3; LYN; RELA; MAPK3; NFKB1; CDKN1A; CASP8
68	fca04210	0.0763889	8.09802	8.37096	TP53; NFKBIA; MAPK1; CASP3; TNF; RELA; MAPK3; NFKB1; CASP8; BCL2; FASLG
69	fca04919	0.0833333	8.8342	8.4241	TP53; MTOR; MAPK1; HIF1A; CTNNB1; ITGB3; MAPK3; THRA; STAT1; ESR1
70	fca04660	0.0819672	8.68938	8.33782	PTPRC; NFKBIA; MAPK1; IFNG; TNF; RELA; MAPK3; NFKB1; IL2; IL10
71	fca05230	0.114286	12.1155	9.10622	TP53; EGFR; MTOR; FGFR1; MAPK1; HIF1A; MAPK3; SIRT6
72	fca04915	0.0729927	7.73799	7.74811	PRKCD; EGFR; MAPK1; MMP9; NOS3; ESR2; MAPK3; MMP2; BCL2; ESR1
73	fca04010	0.0466667	4.94715	6.7718	TP53; EGFR; FGFR1; MAPK1; CASP3; TNF; IL1B; RELA; MAPK3; NFKB1; TGFB1; CACNB4; MYD88; FASLG
74	fca05010	0.04	4.24042	6.45209	MTOR; XBP1; MAPK1; CTNNB1; CASP3; TNF; IL6; IL1B; RELA; MAPK3; NOS2; APOE; NFKB1; PTGS2; CASP8; CHRNA7
75	fca05216	0.166667	17.6684	9.77636	TP53; PPARG; MAPK1; CTNNB1; MAPK3; CDKN1A
76	fca04928	0.0782609	8.29647	7.67877	EGFR; FGFR1; MAPK1; MAPK3; TNFSF11; VDR; CDKN1A; MMP14; BCL2
77	fca04062	0.0588235	6.23591	7.05184	PRKCD; NFKBIA; MAPK1; STAT3; CXCL8; LYN; RELA; MAPK3; NFKB1; JAK2; STAT1
78	fca05208	0.0506329	5.36762	6.63893	PRKCD; EGFR; NFKBIA; MAPK1; HMOX1; HIF1A; RELA; MAPK3; NFKB1; LPO; NFE2L2; AHR
79	fca04722	0.0731707	7.75686	7.35699	PRKCD; TP53; NFKBIA; MAPK1; RELA; MAPK3; NFKB1; BCL2; FASLG
80	fca04613	0.0547264	5.80157	6.70927	ELANE; TLR4; MTOR; MAPK1; SYK; HMGB1; ITGB3; TLR2; RELA; MAPK3; NFKB1
81	fca05332	0.139535	14.7922	8.84399	IFNG; TNF; IL6; IL1B; IL2; FASLG
82	fca05022	0.0346232	3.67042	5.91862	MTOR; XBP1; MAPK1; CTNNB1; CASP3; TNF; IL6; IL1B; RELA; MAPK3; NOS2; NFKB1; PTGS2; CASP8; CHRNA7; BCL2; FASLG
83	fca05218	0.0985915	10.4517	7.79872	TP53; E2F2; EGFR; FGFR1; MAPK1; MAPK3; CDKN1A
84	fca05223	0.0985915	10.4517	7.79872	TP53; E2F2; EGFR; MAPK1; STAT3; MAPK3; CDKN1A
85	fca04920	0.0958904	10.1654	7.66898	NFKBIA; MTOR; STAT3; TNF; RELA; NFKB1; JAK2
86	fca05214	0.0933333	9.89431	7.54417	TP53; E2F2; EGFR; MTOR; MAPK1; MAPK3; CDKN1A
87	fca01523	0.172414	18.2777	9.09214	TNF; IL6; IL1B; RELA; NFKB1
88	fca04662	0.0897436	9.51376	7.36549	NFKBIA; MAPK1; SYK; LYN; RELA; MAPK3; NFKB1
89	fca04060	0.0427046	4.52714	5.85083	CXCL8; IFNG; TNF; IL6; IL1B; IL17A; TNFSF11; IL2; TGFB1; IL33; IL10; FASLG
90	fca05213	0.105263	11.159	7.50534	TP53; EGFR; MAPK1; CTNNB1; MAPK3; CDKN1A
91	fca04071	0.0634921	6.73082	6.3164	TP53; MAPK1; NOS3; TNF; RELA; MAPK3; NFKB1; BCL2
92	fca04520	0.0752688	7.97928	6.59793	EGFR; FGFR1; MAPK1; CTNNB1; MAPK3; TJP1; SMAD3
93	fca05330	0.128205	13.5911	7.68761	IFNG; TNF; IL2; IL10; FASLG

No.	KEGG ID	Rich factor	Fold enrichment	z score	Contributing genes
94	fca05221	0.0895522	9.49347	6.80643	MTOR; MAPK1; STAT3; RELA; MAPK3; NFKB1
95	fca05415	0.0456621	4.84066	5.60679	PRKCD; MTOR; MMP9; NOS3; COL1A1; RELA; NFKB1; TGFB1; MMP2; SMAD3
96	fca05202	0.0491803	5.21363	5.61286	ELANE; TP53; PPARG; MMP9; CXCL8; IL6; RELA; NFKB1; CDKN1A
97	fca04622	0.0833333	8.8342	6.50976	NFKBIA; CXCL8; TNF; RELA; NFKB1; CASP8
98	fca04650	0.0666667	7.06736	6.0983	MAPK1; SYK; IFNG; CASP3; TNF; MAPK3; FASLG
99	fca04550	0.0559441	5.93065	5.79439	JAK1; FGFR1; MAPK1; STAT3; CTNNB1; MAPK3; JAK2; SMAD3
100	fca04940	0.111111	11.7789	7.07142	IFNG; TNF; IL1B; IL2; FASLG
101	fca04930	0.106383	11.2777	6.89148	PRKCD; MTOR; MAPK1; TNF; MAPK3
102	fca04350	0.0630631	6.68534	5.87708	MAPK1; IFNG; TNF; MAPK3; TGFB1; THBS1; SMAD3
103	fca04115	0.0769231	8.15465	6.18969	TP53; CASP3; CDKN1A; THBS1; CASP8; BCL2
104	fca04510	0.0445545	4.72324	5.2157	CAV1; EGFR; MAPK1; CTNNB1; ITGB3; COL1A1; MAPK3; THBS1; BCL2
105	fca04611	0.057377	6.08256	5.51117	MAPK1; SYK; NOS3; ITGB3; COL1A1; LYN; MAPK3
106	fca04211	0.0681818	7.22798	5.72581	TP53; PPARG; MTOR; SIRT1; RELA; NFKB1
107	fca04666	0.0645161	6.83938	5.52032	PRKCD; PTPRC; MAPK1; SYK; LYN; MAPK3
108	fca04912	0.0645161	6.83938	5.52032	PRKCD; EGFR; MAPK1; MAPK3; MMP2; MMP14
109	fca04664	0.0735294	7.79488	5.48599	MAPK1; SYK; TNF; LYN; MAPK3
110	fca04921	0.0463576	4.91439	4.72891	EGFR; MAPK1; NOS3; MAPK3; PTGS2; CDKN1A; CACNB4
111	fca05211	0.0694444	7.36183	5.28631	MAPK1; HIF1A; MAPK3; CDKN1A; TGFB1
112	fca04934	0.0454545	4.81866	4.65955	E2F2; EGFR; MAPK1; CTNNB1; MAPK3; CDKN1A; AHR
113	fca04145	0.0354331	3.75627	4.341	ELANE; TLR4; MMP9; SYK; ITGB3; TLR2; ALPI; LTF; THBS1
114	fca04518	0.0443038	4.69666	4.56982	EGFR; MTOR; CTNNB1; ITGB3; COL1A1; TGFB1; THBS1
115	fca04935	0.0508475	5.39036	4.68097	MTOR; MAPK1; STAT3; MAPK3; JAK2; STAT1
116	fca04672	0.0869565	9.2183	5.45139	IL6; IL2; TGFB1; IL10
117	fca03272	0.0816327	8.65391	5.24073	DPP4; EGFR; CLDN2; OCLN
118	fca04012	0.0595238	6.31014	4.76879	EGFR; MTOR; MAPK1; MAPK3; CDKN1A
119	fca04140	0.0391061	4.14566	4.14337	PRKCD; MTOR; MAPK1; HIF1A; HMGB1; MAPK3; BCL2
120	fca04061	0.0561798	5.95564	4.5821	CXCL8; TNF; IL6; IL2; IL10
121	fca04371	0.0425532	4.51108	4.09678	MTOR; MAPK1; NOS3; MAPK3; NOS2; SMAD3
122	fca04370	0.0689655	7.31106	4.70349	MAPK1; NOS3; MAPK3; PTGS2
123	fca05231	0.0515464	5.46445	4.31118	EGFR; MTOR; MAPK1; HIF1A; MAPK3
124	fca05410	0.05	5.30052	4.21723	ITGB3; TNF; IL6; TGFB1; CACNB4
125	fca04072	0.0392157	4.15727	3.83981	EGFR; MTOR; MAPK1; CXCL8; SYK; MAPK3
126	fca04015	0.0327103	3.46763	3.56012	EGFR; FGFR1; MAPK1; CTNNB1; ITGB3; MAPK3; THBS1
127	fca04725	0.0446429	4.73261	3.87603	MAPK1; MAPK3; JAK2; CHRNA7; BCL2
128	fca04081	0.0321101	3.404	3.50129	STAT3; ESR2; ITGB3; GUCA2A; THRA; JAK2; ESR1
129	fca04215	0.0833333	8.8342	4.59492	CASP3; CASP8; BCL2
130	fca04670	0.042735	4.53036	3.74788	CLDN2; MMP9; CTNNB1; OCLN; MMP2
131	fca00760	0.0789474	8.36924	4.44109	SIRT1; ENPP1; SIRT6
132	fca05020	0.0280702	2.97573	3.30127	PRKCD; CAV1; MAPK1; CASP3; TNF; IL6; IL1B; MAPK3
133	fca04014	0.0297872	3.15776	3.26567	EGFR; FGFR1; MAPK1; RELA; MAPK3; NFKB1; FASLG
134	fca04540	0.0465116	4.93072	3.57212	EGFR; MAPK1; MAPK3; TJP1
135	fca04519	0.0234375	2.48462	2.89398	EGFR; NFKBIA; FGFR1; MAPK1; CTNNB1; RELA; MAPK3; NFKB1; TJP1
136	fca05320	0.0625	6.62565	3.81226	IL2; IL10; FASLG
137	fca04640	0.0412371	4.37156	3.25581	ITGB3; TNF; IL6; IL1B
138	fca00220	0.1	10.601	4.19395	NOS3; NOS2
139	fca05414	0.0384615	4.07732	3.0781	ITGB3; TNF; TGFB1; CACNB4
140	fca04110	0.0310559	3.29225	2.86088	TP53; E2F2; CDKN1A; TGFB1; SMAD3
141	fca04730	0.0508475	5.39036	3.30028	MAPK1; LYN; MAPK3
142	fca04517	0.0238908	2.53267	2.5977	FGFR1; MAPK1; CTNNB1; ITGB3; LYN; MAPK3; TJP1
143	fca05217	0.0483871	5.12954	3.18263	TP53; CTNNB1; CDKN1A

No.	KEGG ID	Rich factor	Fold enrichment	z score	Contributing genes
144	fca04726	0.036036	3.8202	2.91531	MAPK1; CASP3; MAPK3; PTGS2
145	fca04929	0.046875	4.96924	3.10835	MAPK1; ESR2; MAPK3
146	fca05310	0.0714286	7.57217	3.3982	TNF; IL10
147	fca05416	0.0410959	4.35659	2.80857	CAV1; CASP3; CASP8
148	fca04710	0.0588235	6.23591	2.98415	RORA; RORC
149	fca04960	0.0540541	5.73029	2.81282	MAPK1; MAPK3
150	fca05412	0.0344828	3.65553	2.42738	CTNNB1; ITGB3; CACNB4
151	fca05322	0.027027	2.86515	2.23042	ELANE; IFNG; TNF; IL10
152	fca04512	0.032967	3.49485	2.3328	ITGB3; COL1A1; THBS1
153	fca04261	0.0263158	2.78975	2.16942	MAPK1; MAPK3; CACNB4; BCL2
154	fca04975	0.0487805	5.17124	2.61154	FABP2; ABCA1
155	fca04024	0.0222222	2.35579	2.00678	NFKBIA; MAPK1; RELA; MAPK3; NFKB1
156	fca04150	0.0253165	2.68381	2.08152	MTOR; MAPK1; TNF; MAPK3
157	fca03410	0.0444444	4.71157	2.43495	FEN1; HMGB1
158	fca04974	0.03	3.18031	2.13809	DPP4; ACE2; COL1A1
159	fca04810	0.0214592	2.2749	1.92108	EGFR; FGFR1; MAPK1; ITGB3; MAPK3
160	fca04916	0.029703	3.14882	2.11782	MAPK1; CTNNB1; MAPK3
161	fca05014	0.0181818	1.92746	1.81035	TP53; MTOR; XBP1; CASP3; TNF; NOS2; BCL2
162	fca04137	0.0294118	3.11795	2.09781	TP53; HIF1A; RELA
163	fca02010	0.0425532	4.51108	2.35428	ABCB1; ABCA1
164	fca00740	0.125	13.2513	3.38268	ENPP1
165	fca04913	0.0416667	4.4171	2.31562	CYP19A1; PTGS2
166	fca05030	0.0416667	4.4171	2.31562	RELA; NFKB1
167	fca03266	0.111111	11.7789	3.15683	ITGB3
168	fca04310	0.0228571	2.4231	1.85303	TP53; SIRT1; CTNNB1; SMAD3
169	fca00330	0.0384615	4.07732	2.17095	NOS3; NOS2
170	fca04961	0.0384615	4.07732	2.17095	VDR; ESR1
171	fca04979	0.0377358	4.00039	2.13704	APOE; ABCA1
172	fca04020	0.0195312	2.07052	1.69281	EGFR; FGFR1; NOS3; NOS2; CHRNA7
173	fca04216	0.0363636	3.85492	2.07164	TP53; HMOX1

Statistical note. The manuscript text should use the same significance definition as the result counts. In the archived files, the counts of 195 GO terms and 150 KEGG pathways correspond to nominal $P < 0.05$, whereas adjusted-$P < 0.05$ yields 107 GO terms and 141 KEGG pathways. Both statistics are retained here to make the distinction explicit.

Orthology resources :
NCBI Gene FTP/Datasets: gene_orthologs documentation and ortholog calculation notes (accessed 25 August 2026).
NCBI Gene: Felis catus LOC111557813, complement C3-like (Gene ID 111557813; accessed 25 August 2026).
Vertebrate Gene Nomenclature Committee (VGNC): feline PTGS1, VGNC:69147 (accessed 25 August 2026).
