# Supplementary material for: Network Pharmacology and Molecular Simulation Reveal Potential Targets and Pathways of Vine Tea in Feline Intestinal Inflammation
Source: Vet Sci. 2026 Aug 31;13(9):895. doi: 10.3390/vetsci13090895 (PMC13611877; doi:10.3390/vetsci13090895)
Supplement: Supplementary file 1 [file vetsci-13-00895-s001.zip › Supplementary S1.pdf]

Supplementary Data S1

Complete Screening Audit, Compound Provenance, Target-Prediction Dataset, and Molecular Docking Reproducibility Data for Vine Tea Candidate Compounds

Reproducible screening workflow: The archived candidate input contained 55 records. Forty-nine unique chemical identities were successfully standardized and evaluated in SwissADME; 17 were retained and 32 were excluded. Four manually added name records duplicated compounds already represented by PubChem identifiers, and two name records did not yield a discrete usable PubChem CID in the archived standardization output. The reconstructed retention rule exactly reproduced the archived 17-compound set: predicted GI absorption = High AND (at least three of the five drug-likeness rules—Lipinski, Ghose, Veber, Egan, and Muegge—showing zero violations OR the maximum number of violations in any one of the five rules being ≤1). This filter was used only as an in silico prioritization step and should not be interpreted as evidence of oral bioavailability in cats.

Chemical-provenance note: No physical vine tea batch, extract, or formulation was chemically analyzed in the present in silico study. The supplied archive preserves a database-coded candidate block labelled "herbs" plus manually added literature/name entries, but it does not preserve an unambiguous per-compound constituent-source field distinguishing HERB from BATMAN-TCM. Therefore, this supplement reports the archived input honestly and adds an independent literature-provenance audit rather than inferring a database source that is not recoverable. Published analytical/isolation evidence indicates that several retained compounds or their neutral parent compounds occur in Ampelopsis grossedentata, but database-encoded ionic or partially specified stereochemical forms are not treated as independently measured phytochemicals.

Target-prediction and docking scope: The 17 retained compounds yielded 966 nonredundant predicted targets after merging TargetNet, SEA, BATMAN-TCM, and SwissTargetPrediction results. The complete database-specific target lists are included in this Supplementary Data S1 rather than supplied as a separate supplement. Molecular docking used 10 representative compounds cross-docked against six core targets (IL6, TNF, IL1B, STAT3, CASP3, and TLR4), giving 60 compound–target combinations. The supplied docking logs/configuration files record AutoDock Vina 1.2.7, scoring function = vina, exhaustiveness = 50, CPU = 4, random seed = 1000, and nine output poses per docking run.

Table S1A. Complete screening audit of all 55 initial vine tea candidate records (17 retained compounds, 32 SwissADME-excluded compounds, 4 duplicate name records, and 2 unresolved records).

No.	Archived input	Standardized compound	PubChem CID	Canonical SMILES	Archived source interpretation	SwissADME screening profile	Screening decision
1	5487255	Myricomplanoside	5487255	COC1=C(C(=CC(=C1)C2=C(C(=O)C3=C(C=C(C=C3O2)O)O)O)O[C@H]4[C@@H]([C@H]([C@@H]([C@H](O4)CO)O)O)O)O	Archived database-coded candidate block (labelled "herbs" in the supplied input); per-database constituent origin was not preserved.	GI Low; L/G/V/E/M = 2/2/1/1/3; 0/5 rules with 0 violations; max individual violations = 3	Excluded at GI-absorption stage (predicted GI absorption = Low).
2	442154	Afzelechin	442154	C1[C@@H]([C@H](OC2=CC(=CC(=C21)O)O)C3=CC=C(C=C3)O)O	Archived database-coded candidate block (labelled "herbs" in the supplied input); per-database constituent origin was not preserved.	GI High; L/G/V/E/M = 0/0/0/0/0; 5/5 rules with 0 violations; max individual violations = 0	Retained. Passed GI = High and the predefined drug-likeness rule.
3	12305858	Ambrein	12305858	C/C(=C\CC[C@@H]1[C@]2(CCCC([C@@H]2CC[C@@]1(C)O)(C)C)C)/CC[C@@H]3C(=C)CCCC3(C)C	Archived database-coded candidate block (labelled "herbs" in the supplied input); per-database constituent origin was not preserved.	GI Low; L/G/V/E/M = 1/3/0/1/2; 1/5 rules with 0 violations; max individual violations = 3	Excluded at GI-absorption stage (predicted GI absorption = Low).
4	161557	Dihydromyricetin	161557	C1=C(C=C(C(=C1O)O)O)[C@@H]2[C@H](C(=O)C3=C(C=C(C=C3O2)O)O)O	Archived database-coded candidate block (labelled "herbs" in the supplied input); per-database constituent origin was not preserved.	GI Low; L/G/V/E/M = 1/0/1/1/1; 1/5 rules with 0 violations; max individual violations = 1	Excluded at GI-absorption stage (predicted GI absorption = Low).
5	Andmyricetin-3-o-beta-d-galactopyranoside	Andmyricetin-3-o-beta-d-galactopyranosideAndmyricetin-3-o-beta-d-galactopyranoside	—	—	Archived database-coded candidate block (labelled "herbs" in the supplied input); per-database constituent origin was not preserved.	Not evaluated	No discrete usable PubChem CID was recovered from the archived standardization output; excluded before SwissADME.
6	5280746	Apiin	5280746	C1[C@@]([C@H]([C@@H](O1)O[C@@H]2[C@H]([C@@H]([C@H](O[C@H]2OC3=CC(=C4C(=C3)OC(=CC4=O)C5=CC=C(C=C5)O)O)CO)O)O)O)(CO)O	Archived database-coded candidate block (labelled "herbs" in the supplied input); per-database constituent origin was not preserved.	GI Low; L/G/V/E/M = 3/3/1/1/3; 0/5 rules with 0 violations; max individual violations = 3	Excluded at GI-absorption stage (predicted GI absorption = Low).
7	3220	Emodin	3220	CC1=CC2=C(C(=C1)O)C(=O)C3=C(C2=O)C=C(C=C3O)O	Archived database-coded candidate block (labelled "herbs" in the supplied input); per-database constituent origin was not preserved.	GI High; L/G/V/E/M = 0/0/0/0/0; 5/5 rules with 0 violations; max individual violations = 0	Retained. Passed GI = High and the predefined drug-likeness rule.
8	5282102	Astragalin	5282102	C1=CC(=CC=C1C2=C(C(=O)C3=C(C=C(C=C3O2)O)O)O[C@H]4[C@@H]([C@H]([C@@H]([C@H](O4)CO)O)O)O)O	Archived database-coded candidate block (labelled "herbs" in the supplied input); per-database constituent origin was not preserved.	GI Low; L/G/V/E/M = 2/0/1/1/3; 1/5 rules with 0 violations; max individual violations = 3	Excluded at GI-absorption stage (predicted GI absorption = Low).
9	5281623	Bellidifolin	5281623	COC1=CC(=C2C(=C1)OC3=C(C=CC(=C3C2=O)O)O)O	Archived database-coded candidate block (labelled "herbs" in the supplied input); per-database constituent origin was not preserved.	GI High; L/G/V/E/M = 0/0/0/0/0; 5/5 rules with 0 violations; max individual violations = 0	Retained. Passed GI = High and the predefined drug-likeness rule.
10	222284	Beta-Sitosterol	222284	CC[C@H](CC[C@@H](C)[C@H]1CC[C@@H]2[C@@]1(CC[C@H]3[C@H]2CC=C4[C@@]3(CC[C@@H](C4)O)C)C)C(C)C	Archived database-coded candidate block (labelled "herbs" in the supplied input); per-database constituent origin was not preserved.	GI Low; L/G/V/E/M = 1/3/0/1/2; 1/5 rules with 0 violations; max individual violations = 3	Excluded at GI-absorption stage (predicted GI absorption = Low).
11	65575	Cedrol	65575	C[C@@H]1CC[C@@H]2[C@]13CC[C@@]([C@H](C3)C2(C)C)(C)O	Archived database-coded candidate block (labelled "herbs" in the supplied input); per-database constituent origin was not preserved.	GI High; L/G/V/E/M = 0/0/0/0/1; 4/5 rules with 0 violations; max individual violations = 1	Retained. Passed GI = High and the predefined drug-likeness rule.
12	985	Palmitic Acid	985	CCCCCCCCCCCCCCCC(=O)O	Archived database-coded candidate block	GI High; L/G/V/E/M = 1/0/1/0/1; 2/5	Retained. Passed GI = High and the

No.	Archived input	Standardized compound	PubChem CID	Canonical SMILES	Archived source interpretation	SwissADME screening profile	Screening decision
					(labelled “herbs” in the supplied input); per-database constituent origin was not preserved.	rules with 0 violations; max individual violations = 1	predefined drug-likeness rule.
13	122850	Aromadendrin	122850	C1=CC(=CC=C1[C@@H]2[C@H](C(=O)C3=C(C=C(C=C3O2)O)O)O)O	Archived database-coded candidate block (labelled “herbs” in the supplied input); per-database constituent origin was not preserved.	GI High; L/G/V/E/M = 0/0/0/0/0; 5/5 rules with 0 violations; max individual violations = 0	Retained. Passed GI = High and the predefined drug-likeness rule.
14	5280863	Kaempferol	5280863	C1=CC(=CC=C1C2=C(C(=O)C3=C(C=C(C=C3O2)O)O)O)O	Archived database-coded candidate block (labelled “herbs” in the supplied input); per-database constituent origin was not preserved.	GI High; L/G/V/E/M = 0/0/0/0/0; 5/5 rules with 0 violations; max individual violations = 0	Retained. Passed GI = High and the predefined drug-likeness rule.
15	5281672	Myricetin	5281672	C1=C(C=C(C(=C1O)O)O)C2=C(C(=O)C3=C(C=C(C=C3O2)O)O)O	Archived database-coded candidate block (labelled “herbs” in the supplied input); per-database constituent origin was not preserved.	GI Low; L/G/V/E/M = 1/0/1/1/2; 1/5 rules with 0 violations; max individual violations = 2	Excluded at GI-absorption stage (predicted GI absorption = Low).
16	5352000	Myricetin 3-rhamnoside	5352000	CC1C(C(C(C(O1)OC2=C(OC3=CC(=CC(=C3C2=O)O)O)C4=CC(=C(C(=C4)O)O)O)O)O)O	Archived database-coded candidate block (labelled “herbs” in the supplied input); per-database constituent origin was not preserved.	GI Low; L/G/V/E/M = 2/0/1/1/3; 1/5 rules with 0 violations; max individual violations = 3	Excluded at GI-absorption stage (predicted GI absorption = Low).
17	Myricetin-3-o-beta-d-(6"-o-galloyl)-galactopyranoside	Myricetin-3-o-beta-d-(6"-o-galloyl)-galactopyranosideMyricetin-3-o-beta-d-(6"-o-galloyl)-galactopyranoside	—	—	Archived database-coded candidate block (labelled “herbs” in the supplied input); per-database constituent origin was not preserved.	Not evaluated	No discrete usable PubChem CID was recovered from the archived standardization output; excluded before SwissADME.
18	5491408	Myricetin 3-galactoside	5491408	C1=C(C=C(C(=C1O)O)O)C2=C(C(=O)C3=C(C=C(C=C3O2)O)O)O[C@H]4[C@@H]([C@H]([C@H]([C@H](O4)CO)O)O)O	Archived database-coded candidate block (labelled “herbs” in the supplied input); per-database constituent origin was not preserved.	GI Low; L/G/V/E/M = 2/2/1/1/3; 0/5 rules with 0 violations; max individual violations = 3	Excluded at GI-absorption stage (predicted GI absorption = Low).
19	5318606	myricetin 3-O-beta-D-glucopyranoside	5318606	C1=C(C=C(C(=C1O)O)O)C2=C(C(=O)C3=C(C=C(C=C3O2)O)O)O[C@H]4[C@@H]([C@H]([C@@H]([C@H](O4)CO)O)O)O	Archived database-coded candidate block (labelled “herbs” in the supplied input); per-database constituent origin was not preserved.	GI Low; L/G/V/E/M = 2/2/1/1/3; 0/5 rules with 0 violations; max individual violations = 3	Excluded at GI-absorption stage (predicted GI absorption = Low).
20	23900088	myricetin-3-O-beta-D-xylopyranoside	23900088	C1[C@H]([C@@H]([C@H]([C@@H](O1)OC2=C(OC3=CC(=CC(=C3C2=O)O)O)C4=CC(=C(C(=C4)O)O)O)O)O)O	Archived database-coded candidate block (labelled “herbs” in the supplied input); per-database constituent origin was not preserved.	GI Low; L/G/V/E/M = 2/0/1/1/3; 1/5 rules with 0 violations; max individual violations = 3	Excluded at GI-absorption stage (predicted GI absorption = Low).
21	5281673	Myricitrin	5281673	C[C@H]1[C@@H]([C@H]([C@H]([C@@H](O1)OC2=C(OC3=CC(=CC(=C3C2=O)O)O)C4=CC(=C(C(=C4)O)O)O)O)O)O	Archived database-coded candidate block (labelled “herbs” in the supplied input); per-database constituent origin was not preserved.	GI Low; L/G/V/E/M = 2/0/1/1/3; 1/5 rules with 0 violations; max individual violations = 3	Excluded at GI-absorption stage (predicted GI absorption = Low).
22	10639	Physcion	10639	CC1=CC2=C(C(=C1)O)C(=O)C3=C(C2=O)C=C(C=C3O)OC	Archived database-coded candidate block (labelled “herbs” in the supplied input); per-database constituent origin was not preserved.	GI High; L/G/V/E/M = 0/0/0/0/0; 5/5 rules with 0 violations; max individual violations = 0	Retained. Passed GI = High and the predefined drug-likeness rule.
23	5280435	Phytol	5280435	C[C@@H](CCC[C@@H](C)CCC/C(=C/CO)/C)CCCC(C)C	Archived database-coded candidate block (labelled “herbs” in the supplied input); per-database constituent origin was not preserved.	GI Low; L/G/V/E/M = 1/1/1/1/2; 0/5 rules with 0 violations; max individual violations = 2	Excluded at GI-absorption stage (predicted GI absorption = Low).
24	5280343	Quercetin	5280343	C1=CC(=C(C=C1C2=C(C(=O)C3=C(C=C(C=C3O2)O)O)O)O)O	Archived database-coded candidate block (labelled “herbs” in the supplied input); per-database constituent origin was not preserved.	GI High; L/G/V/E/M = 0/0/0/0/0; 5/5 rules with 0 violations; max individual violations = 0	Retained. Passed GI = High and the predefined drug-likeness rule.
25	6325794	2-(3,4-dihydroxyphenyl)-5,7-dihydroxy-3-[(2S,3R,4R,5S,6S)-3,4,5-trihydroxy-6-methyloxan-2-yl]oxychromen-4-one	6325794	C[C@H]1[C@H]([C@H]([C@H]([C@@H](O1)OC2=C(OC3=CC(=CC(=C3C2=O)O)O)C4=CC(=C(C=C4)O)O)O)O)O	Archived database-coded candidate block (labelled “herbs” in the supplied input); per-database constituent origin was not preserved.	GI Low; L/G/V/E/M = 2/0/1/1/3; 1/5 rules with 0 violations; max individual violations = 3	Excluded at GI-absorption stage (predicted GI absorption = Low).
26	439533	Taxifolin	439533	C1=CC(=C(C=C1[C@@H]2[C@H](C(=O)C3=C(C=C(C=C3O2)O)O)O)O)O	Archived database-coded candidate block (labelled “herbs” in the supplied input); per-database constituent origin was not preserved.	GI High; L/G/V/E/M = 0/0/0/0/0; 5/5 rules with 0 violations; max individual violations = 0	Retained. Passed GI = High and the predefined drug-likeness rule.
27	25202062	Kaempferol oxoanion	25202062	C1=CC(=CC=C1C2=C(C(=O)C3=C(C=C(C=C3O2)O)O)[O-])O	Archived database-coded candidate block (labelled “herbs” in the supplied input); per-database constituent origin was not preserved.	GI High; L/G/V/E/M = 0/0/0/0/0; 5/5 rules with 0 violations; max individual violations = 0	Retained. Passed GI = High and the predefined drug-likeness rule.
28	44259441	3,3',4',5,5',7-Hexahydroxyflavone 3-O-Rhamnoside	44259441	CC1[C@@H](C([C@@H]([C@@H](O1)OC2=C(OC3=CC(=CC(=C3C2=O)O)O)C4=CC(=C(C(=C4)O)O)O)O)O)O	Archived database-coded candidate block (labelled “herbs” in the supplied input); per-database constituent origin was not preserved.	GI Low; L/G/V/E/M = 2/0/1/1/3; 1/5 rules with 0 violations; max individual violations = 3	Excluded at GI-absorption stage (predicted GI absorption = Low).
29	73755987	7-[3-[(4R)-3,4-dihydroxy-4-	73755987	C1[C@@](C(C(O1)OC2C(C(C(OC2OC3=CC(=C4C(	Archived database-coded candidate block	GI Low; L/G/V/E/M = 3/3/1/1/3; 0/5	Excluded at GI-absorption stage (predicted

No.	Archived input	Standardized compound	PubChem CID	Canonical SMILES	Archived source interpretation	SwissADME screening profile	Screening decision
		(hydroxymethyl)oxolan-2-yl]oxy-4,5-dihydroxy-6-(hydroxymethyl)oxan-2-yl]oxy-5-hydroxy-2-(4-hydroxyphenyl)chromen-4-one		=C3)OC(=CC4=O)C5=CC=C(C=C5)O)O)CO)O)O)O)(CO)O	(labelled "herbs" in the supplied input); per-database constituent origin was not preserved.	rules with 0 violations; max individual violations = 3	GI absorption = Low).
30	161558	alpha-D-Glucopyranoside, beta-D-fructofuranosyl, monotetradecanoate	161558	CCCCCCCCCCCCCC(=O)O[C@@]1([C@@H]([C@H]([C@@H]([C@H](O1)CO)O)O)O)[C@@]2([C@H]([C@@H]([C@H](O2)CO)O)O)CO	Archived database-coded candidate block (labelled "herbs" in the supplied input); per-database constituent origin was not preserved.	GI Low; L/G/V/E/M = 3/4/2/1/4; 0/5 rules with 0 violations; max individual violations = 4	Excluded at GI-absorption stage (predicted GI absorption = Low).
31	40486293	2-(3,4-dihydroxyphenyl)-5,7-dihydroxy-3-[(2S,3S,4S,5S,6R)-3,4,5-trihydroxy-6-methyloxan-2-yl]oxychromen-4-one	40486293	C[C@@H]1[C@H]([C@@H]([C@@H]([C@@H](O1)OC2=C(OC3=CC(=CC(=C3C2=O)O)O)C4=CC(=C(C=C4)O)O)O)O)O	Archived database-coded candidate block (labelled "herbs" in the supplied input); per-database constituent origin was not preserved.	GI Low; L/G/V/E/M = 2/0/1/1/3; 1/5 rules with 0 violations; max individual violations = 3	Excluded at GI-absorption stage (predicted GI absorption = Low).
32	44123431	(1S,2R,8S)-2,6,6,8-tetramethyltricyclo[5.3.1.01,5]undecan-8-ol	44123431	C[C@@H]1CCC2[C@]13CC[C@](C(C3)C2(C)C)(C)O	Archived database-coded candidate block (labelled "herbs" in the supplied input); per-database constituent origin was not preserved.	GI High; L/G/V/E/M = 0/0/0/0/1; 4/5 rules with 0 violations; max individual violations = 1	Retained. Passed GI = High and the predefined drug-likeness rule.
33	11826112	CID 11826112	11826112	C/C(=C\CC[C@@H]1[C@]2(CCCC(C2CC[C@@]1(C)O)(C)C)C)/CC[C@@H]3C(=C)CCCC3(C)C	Archived database-coded candidate block (labelled "herbs" in the supplied input); per-database constituent origin was not preserved.	GI Low; L/G/V/E/M = 1/3/0/1/2; 1/5 rules with 0 violations; max individual violations = 3	Excluded at GI-absorption stage (predicted GI absorption = Low).
34	46906036	Quercetin-7-olate	46906036	C1=CC(=C(C=C1C2=C(C(=O)C3=C(C=C(C=C3O2)O)O)[O-])O)O	Archived database-coded candidate block (labelled "herbs" in the supplied input); per-database constituent origin was not preserved.	GI High; L/G/V/E/M = 0/0/0/1/0; 4/5 rules with 0 violations; max individual violations = 1	Retained. Passed GI = High and the predefined drug-likeness rule.
35	25201643	Myricetin(1-)	25201643	C1=C(C=C(C(=C1O)[O-])O)C2=C(C(=O)C3=C(C=C(C=C3O2)O)O)O	Archived database-coded candidate block (labelled "herbs" in the supplied input); per-database constituent origin was not preserved.	GI Low; L/G/V/E/M = 0/0/1/1/1; 2/5 rules with 0 violations; max individual violations = 1	Excluded at GI-absorption stage (predicted GI absorption = Low).
36	44257793	7-[(2S,4S,5S)-3-[(2S,4S)-3,4-dihydroxy-4-(hydroxymethyl)oxolan-2-yl]oxy-4,5-dihydroxy-6-(hydroxymethyl)oxan-2-yl]oxy-5-hydroxy-2-(4-hydroxyphenyl)chromen-4-one	44257793	C1[C@](C([C@@H](O1)OC2[C@H]([C@@H](C(O[C@H]2OC3=CC(=C4C(=C3)OC(=CC4=O)C5=CC=C(C=C5)O)O)CO)O)O)O)(CO)O	Archived database-coded candidate block (labelled "herbs" in the supplied input); per-database constituent origin was not preserved.	GI Low; L/G/V/E/M = 3/3/1/1/3; 0/5 rules with 0 violations; max individual violations = 3	Excluded at GI-absorption stage (predicted GI absorption = Low).
37	118855455	(3S)-3,5,7-trihydroxy-2-(4-hydroxyphenyl)-2,3-dihydrochromen-4-one	118855455	C1=CC(=CC=C1C2[C@@H](C(=O)C3=C(C=C(C=C3O2)O)O)O)O	Archived database-coded candidate block (labelled "herbs" in the supplied input); per-database constituent origin was not preserved.	GI High; L/G/V/E/M = 0/0/0/0/0; 5/5 rules with 0 violations; max individual violations = 0	Retained. Passed GI = High and the predefined drug-likeness rule.
38	44258797	CID 44258797	44258797	C1=CC(=CC=C1C2=C(C(=O)C3=C(C=C(C=C3O2)O)O)O[C@H]4C(C([C@@H]([C@H](O4)CO)O)O)O)O	Archived database-coded candidate block (labelled "herbs" in the supplied input); per-database constituent origin was not preserved.	GI Low; L/G/V/E/M = 2/0/1/1/3; 1/5 rules with 0 violations; max individual violations = 3	Excluded at GI-absorption stage (predicted GI absorption = Low).
39	5366244	3,7,11,15-Tetramethyl-2-hexadecen-1-OL	5366244	CC(C)CCCC(C)CCCC(C)CCC/C(=C/CO)/C	Archived database-coded candidate block (labelled "herbs" in the supplied input); per-database constituent origin was not preserved.	GI Low; L/G/V/E/M = 1/1/1/1/2; 0/5 rules with 0 violations; max individual violations = 2	Excluded at GI-absorption stage (predicted GI absorption = Low).
40	9943440	(2R)-2-(4-hydroxyphenyl)-3,4-dihydro-2H-chromene-3,5,7-triol	9943440	C1C([C@H](OC2=CC(=CC(=C21)O)O)C3=CC=C(C=C3)O)O	Archived database-coded candidate block (labelled "herbs" in the supplied input); per-database constituent origin was not preserved.	GI High; L/G/V/E/M = 0/0/0/0/0; 5/5 rules with 0 violations; max individual violations = 0	Retained. Passed GI = High and the predefined drug-likeness rule.
41	9890615	5,7-dihydroxy-3-[(2R,3S,4S,5S,6R)-3,4,5-trihydroxy-6-methyl-tetrahydropyran-2-yl]oxy-2-(3,4,5-trihydroxyphenyl)chromen-4-one	9890615	C[C@@H]1[C@H]([C@@H]([C@@H]([C@H](O1)OC2=C(OC3=CC(=CC(=C3C2=O)O)O)C4=CC(=C(C(=C4)O)O)O)O)O)O	Archived database-coded candidate block (labelled "herbs" in the supplied input); per-database constituent origin was not preserved.	GI Low; L/G/V/E/M = 2/0/1/1/3; 1/5 rules with 0 violations; max individual violations = 3	Excluded at GI-absorption stage (predicted GI absorption = Low).
42	138911127	Myricetin 3-O-alpha-L-rhamnoside	138911127	C[C@H]1[C@@H]([C@H]([C@H]([C@@H](O1)OC2=C(OC3=CC(=CC(=C3C2=O)O)O)C4=CC(=C(C(=C4)O)[O-])O)O)O)O	Archived database-coded candidate block (labelled "herbs" in the supplied input); per-database constituent origin was not preserved.	GI Low; L/G/V/E/M = 2/0/1/1/3; 1/5 rules with 0 violations; max individual violations = 3	Excluded at GI-absorption stage (predicted GI absorption = Low).
43	11301173	(E,7R,11R)-15-methyl-3,7,11-tri((113C)methyl)(1,5,9,13,16-13C5)hexadec-2-en-1-ol	11301173	CC([13CH3])C[13CH2]C[C@@H]([13CH3])C[13CH2]C[C@@H]([13CH3])C[13CH2]C/C(=C/[13CH2]O)/[13CH3]	Archived database-coded candidate block (labelled "herbs" in the supplied input); per-database constituent origin was not preserved.	GI Low; L/G/V/E/M = 1/1/1/1/2; 0/5 rules with 0 violations; max individual violations = 2	Excluded at GI-absorption stage (predicted GI absorption = Low).
44	25244375	(2R,3R)-3,7-dihydroxy-4-oxo-2-(3,4,5-trihydroxyphenyl)-2,3-dihydrochromen-5-olate	25244375	C1=C(C=C(C(=C1O)O)O)[C@@H]2[C@H](C(=O)C3=C(C=C(C=C3O2)O)[O-])O	Manual literature/name addition in the supplied input.	GI Low; L/G/V/E/M = 0/0/1/1/1; 2/5 rules with 0 violations; max individual violations = 1	Excluded at GI-absorption stage (predicted GI absorption = Low).
45	86289515	(1R,2R,4aS,8aS)-1-{(3E)-6-[(1S)-2,2-dimethyl-6-methylidenecyclohexyl]-4-	86289515	C/C(=C\CC[C@@H]1[C@]2(CCCC([C@@H]2CC[C@@]1(C)O)(C)C)C)/CCC3C(=C)CCCC3(C)C	Manual literature/name addition in the supplied input.	GI Low; L/G/V/E/M = 1/3/0/1/2; 1/5 rules with 0 violations; max individual violations = 3	Excluded at GI-absorption stage (predicted GI absorption = Low).

No.	Archived input	Standardized compound	PubChem CID	Canonical SMILES	Archived source interpretation	SwissADME screening profile	Screening decision
		methylhex-3-en-1-yl}-2,5,5,8a-tetramethyldecahydronaphthalen-2-ol					
46	dihydromyricetin	Dihydromyricetin	161557	See earlier standardized record	Manual literature/name addition in the supplied input.	Duplicate name input; no separate SwissADME run.	Duplicate of an already represented chemical identity; not counted as a separate candidate.
47	myricitrin	Myricitrin	5281673	See earlier standardized record	Manual literature/name addition in the supplied input.	Duplicate name input; no separate SwissADME run.	Duplicate of an already represented chemical identity; not counted as a separate candidate.
48	quercitrin	Quercitrin	5280459	C[C@H]1[C@@H]([C@H]([C@H]([C@@H](O1)OC2=C(OC3=CC(=CC(=C3C2=O)O)O)C4=CC(=C(C=C4)O)O)O)O)O	Manual literature/name addition in the supplied input.	GI Low; L/G/V/E/M = 2/0/1/1/3; 1/5 rules with 0 violations; max individual violations = 3	Excluded at GI-absorption stage (predicted GI absorption = Low).
49	afzelin	Afzelin	5316673	C[C@H]1[C@@H]([C@H]([C@H]([C@@H](O1)OC2=C(OC3=CC(=CC(=C3C2=O)O)O)C4=CC=C(C=C4)O)O)O)O	Manual literature/name addition in the supplied input.	GI Low; L/G/V/E/M = 1/0/1/1/2; 1/5 rules with 0 violations; max individual violations = 2	Excluded at GI-absorption stage (predicted GI absorption = Low).
50	taxifolin	Taxifolin	439533	See earlier standardized record	Manual literature/name addition in the supplied input.	Duplicate name input; no separate SwissADME run.	Duplicate of an already represented chemical identity; not counted as a separate candidate.
51	quercetin	Quercetin	5280343	See earlier standardized record	Manual literature/name addition in the supplied input.	Duplicate name input; no separate SwissADME run.	Duplicate of an already represented chemical identity; not counted as a separate candidate.
52	gallic acid	Gallic Acid	370	C1=C(C=C(C(=C1O)O)O)C(=O)O	Manual literature/name addition in the supplied input.	GI High; L/G/V/E/M = 0/2/0/0/1; 3/5 rules with 0 violations; max individual violations = 2	Retained. Passed GI = High and the predefined drug-likeness rule.
53	phlorizin	Phlorizin	6072	C1=CC(=CC=C1CCC(=O)C2=C(C=C(C=C2O[C@H]3[C@@H]([C@H]([C@@H]([C@H](O3)CO)O)O)O)O)O)O	Manual literature/name addition in the supplied input.	GI Low; L/G/V/E/M = 1/0/1/1/2; 1/5 rules with 0 violations; max individual violations = 2	Excluded at GI-absorption stage (predicted GI absorption = Low).
54	catechin	Cianidanol	9064	C1[C@@H]([C@H](OC2=CC(=CC(=C21)O)O)C3=CC(=C(C=C3)O)O)O	Manual literature/name addition in the supplied input.	GI High; L/G/V/E/M = 0/0/0/0/0; 5/5 rules with 0 violations; max individual violations = 0	Retained. Passed GI = High and the predefined drug-likeness rule.
55	rutin	Rutin	5280805	C[C@H]1[C@@H]([C@H]([C@H]([C@@H](O1)OC[C@@H]2[C@H]([C@@H]([C@H]([C@@H](O2)OC3=C(OC4=CC(=CC(=C4C3=O)O)O)C5=CC(=C(C=C5)O)O)O)O)O)O)O)O	Manual literature/name addition in the supplied input.	GI Low; L/G/V/E/M = 3/4/1/1/4; 0/5 rules with 0 violations; max individual violations = 4	Excluded at GI-absorption stage (predicted GI absorption = Low).

Note. L/G/V/E/M denote the numbers of violations for Lipinski, Ghose, Veber, Egan, and Muegge, respectively. The human-oriented SwissADME outputs are computational descriptors and are not feline pharmacokinetic measurements. Four duplicate manual-name inputs (dihydromyricetin, myricitrin, taxifolin, and quercetin) were not counted twice because the corresponding chemical identities had already been represented by PubChem-coded entries. Two archived name records could not be standardized to a discrete usable PubChem CID and were excluded before SwissADME.

Table S1B. Provenance audit for the 17 retained vine tea candidate compounds.

No.	Compound	Class	PubChem CID	CAS No.	Archived input source	Provenance evidence / source	Reported concentration/content	Interpretation
1	Afzelechin	Flavan-3-ol	442154	2545-00-8	Archived database-coded candidate input (within “herbs” block)	A: Published A. grossedentata phytochemical literature reports afzelechin in stem/leaf. [S1]	NR in the cited source used here.	Retain.
2	Emodin	Anthraquinone	3220	518-82-1	Archived database-coded candidate input (within “herbs” block)	B: A. grossedentata review lists emodin in stem/leaf based on a volatile/profile source; the primary compound-level table was not independently rechecked in this revision audit. [S1]	NR in the cited source used here.	Retain with provenance caveat.
3	Bellidifolin	Xanthone	5281623	2798-25-6	Archived database-coded candidate input (within “herbs” block)	A: Bellidifolin is reported in A. grossedentata stem/leaf phytochemistry. [S1]	NR in the cited source used here.	Retain.
4	Cedrol	Sesquiterpenoid alcohol	65575	77-53-2	Archived database-coded candidate input (within “herbs” block)	A: Cedrol has published GC-MS/profile support in tender stem/leaf of A. grossedentata. [S1]	NR in the cited source used here.	Retain.
5	Palmitic acid	Saturated fatty acid	985	57-10-3	Archived database-coded candidate input (within “herbs” block)	A: Palmitic acid / n-hexadecanoic acid has published volatile/profile support in A. grossedentata. [S1]	NR in the cited source used here.	Retain.
6	Aromadendrin	Dihydroflavonol	122850	480-20-6	Archived database-coded candidate input (within “herbs” block)	B: Aromadendrin corresponds to dihydrokaempferol; dihydrokaempferol is reported in A. grossedentata phytochemical studies. [S1]	NR in the cited source used here.	Retain; use “aromadendrin (dihydrokaempferol)” consistently.
7	Kaempferol	Flavonol	5280863	520-18-3	Archived database-coded candidate input (within “herbs” block)	A: Free kaempferol has direct A. grossedentata analytical/phytochemical support, including standards-based UPLC-HRMS. [S1,S2]	Detected analytically; no single batch-independent concentration is applicable.	Retain.
8	Physcion	Anthraquinone	10639	521-61-9	Archived database-coded candidate input (within “herbs”	D: Present in the archived database-coded input, but a direct A. grossedentata analytical/isolation report for	NR	Retain only for workflow reproducibility; explicitly label botanical provenance as

No.	Compound	Class	PubChem CID	CAS No.	Archived input source	Provenance evidence / source	Reported concentration/content	Interpretation
					block)	physcion was not located in the current literature audit. —		unconfirmed.
9	Quercetin	Flavonol	5280343	117-39-5	Archived database-coded candidate input (within "herbs" block)	A: Free quercetin has direct A. grossedentata analytical/phytochemical support. [S1,S2]	Detected analytically; reported abundance depends on sample/extraction conditions.	Retain.
10	Taxifolin	Dihydroflavonol	439533	480-18-2	Archived database-coded candidate input (within "herbs" block)	A: Taxifolin/dihydroquercetin has direct isolation and LC-MS/UPLC-HRMS support in A. grossedentata. [S1,S2,S3,S5]	Detected/isolated; no single batch-independent concentration is applicable.	Retain.
11	Kaempferol oxoanion	Database-encoded flavonolate anion	25202062	—	Archived database-coded candidate input (within "herbs" block)	C: Published literature supports the neutral parent kaempferol, but "kaempferol oxoanion" is a database-encoded ionic form and was not verified as a separately isolated plant constituent. [S1,S2]	Not applicable as a separately measured ionic constituent.	Retain only as the archived computational entity; do not describe it as independently detected in vine tea.
12	(1S,2R,8S)-2,6,6,8-tetramethyltricyclo[5.3.1.01,5]undecan-8-ol	Cedrol-related stereochemical record	44123431	—	Archived database-coded candidate input (within "herbs" block)	C: Published literature supports cedrol, but this exact stereochemical database entry was not separately reported as an isolated A. grossedentata constituent. [S1]	Not reported for this exact stereochemical entry.	Retain only as archived computational entity; link interpretation to parent cedrol.
13	Quercetin-7-olate	Database-encoded quercetin anion	46906036	—	Archived database-coded candidate input (within "herbs" block)	C: Published literature supports neutral quercetin, whereas quercetin-7-olate is a database-encoded anionic form and was not independently measured as a plant constituent. [S1,S2]	Not applicable as a separately measured ionic constituent.	Retain only as archived computational entity; link interpretation to parent quercetin.
14	(3S)-3,5,7-trihydroxy-2-(4-hydroxyphenyl)-2,3-dihydrochromen-4-one	Dihydrokaempferol stereochemical record	118855455	—	Archived database-coded candidate input (within "herbs" block)	C: Published literature supports dihydrokaempferol/aromadendrin, but this exact partially specified stereochemical PubChem entry was not separately verified. [S1]	Not reported for this exact stereochemical entry.	Retain only for workflow reproducibility; relate to parent dihydrokaempferol.
15	(2R)-2-(4-hydroxyphenyl)-3,4-dihydro-2H-chromene-3,5,7-triol	Afzelechin stereochemical record	9943440	—	Archived database-coded candidate input (within "herbs" block)	C: Published literature supports afzelechin, but this exact partially specified stereochemical PubChem entry was not separately verified as an isolated constituent. [S1]	Not reported for this exact stereochemical entry.	Retain only for workflow reproducibility; relate to parent afzelechin.
16	Gallic acid	Phenolic acid	370	149-91-7	Manual literature/name addition ("gallic acid"), then PubChem-standardized to CID 370	A: Gallic acid was directly isolated from A. grossedentata and is repeatedly reported in phytochemical studies. [S1,S3]	NR in the cited source used here.	Retain.
17	Cianidanol [(+)-catechin]	Flavan-3-ol	9064	154-23-4	Manual literature/name addition ("catechin"), then PubChem-standardized to CID 9064 (cianidanol/(+)-catechin)	A: Cianidanol is (+)-catechin; (+)-catechin was identified in vine tea extract by UPLC-Q-TOF-MS, and catechin is also reported in A. grossedentata phenolic profiles. [S1,S4]	Detected; the cited VTE study reports relative abundance rather than a universal concentration.	Retain; standardize manuscript name as "cianidanol [(+)-catechin]" at first occurrence.

Note. Provenance grades: A = direct published A. grossedentata analytical/isolation evidence for the exact neutral/common compound; B = published A. grossedentata evidence at a synonymous/parent/common-name level or through a secondary phytochemical synthesis, with a minor identity caveat; C = the neutral parent compound is supported, but the exact database-encoded ionic or stereochemical entity was not independently demonstrated as a plant constituent; D = botanical provenance was not independently confirmed in the current audit. "NR" indicates not reported in the cited source used for this audit. These grades describe provenance confidence, not biological activity or in vivo exposure.

Table S1C. Compound-level target-prediction audit summary for the 17 retained vine tea candidate compounds.

No.	Compound	PubChem CID	BATMAN-TCM	SEA	TargetNet	SwissTargetPrediction	Merged unique targets
1	Afzelechin	442154	0	63	27	45	109
2	Emodin	3220	94	12	49	41	169
3	Bellidifolin	5281623	0	0	60	43	95
4	Cedrol	65575	13	16	29	96	137
5	Palmitic acid	985	306	15	61	73	416
6	Aromadendrin	122850	0	111	38	79	195
7	Kaempferol	5280863	62	62	53	97	201
8	Physcion	10639	6	16	54	43	101
9	Quercetin	5280343	217	26	55	71	314
10	Taxifolin	439533	11	57	41	97	148
11	Kaempferol oxoanion	25202062	2	95	51	98	154
12	(1S,2R,8S)-2,6,6,8-tetramethyltricyclo[5.3.1.01,5]undecan-8-ol	44123431	13	0	29	43	79
13	Quercetin-7-olate	46906036	0	15	53	73	111
14	(3S)-3,5,7-trihydroxy-2-(4-hydroxyphenyl)-2,3-dihydrochromen-4-one	118855455	0	58	38	98	145
15	(2R)-2-(4-hydroxyphenyl)-3,4-dihydro-2H-chromene-3,5,7-triol	9943440	0	16	27	96	115
16	Gallic acid	370	42	55	72	35	163

No.	Compound	PubChem CID	BATMAN-TCM	SEA	TargetNet	SwissTargetPrediction	Merged unique targets
17	Cianidanol [(+)-catechin]	9064	4	27	32	96	122

Note. Target-prediction thresholds in the archived workflow/manuscript were TargetNet probability ≥0.90, SEA probability ≥0.30, BATMAN-TCM score ≥80, and SwissTargetPrediction probability >0.10. Counts in database-specific columns are unique targets retained from that database; the merged total is deduplicated across databases and therefore is not the arithmetic sum of the four columns. Across all compounds, the merged target set contained 966 unique targets.

Table S1D. Complete database-specific predicted-target lists for each retained vine tea candidate compound.

1. Afzelechin (PubChem CID 442154)

Target set	n	Gene symbols
Merged unique	109	ABCB1; ABCC1; ABCG2; ACHE; ACVRL1; ADRA2C; AHR; AKR1B1; AKR1B10; ALDH2; ALK; ALOX12; ALOX15; ALOX5; ALPI; AMY1A; ANTXR2; APP; AR; ATG4B; AURKB; AXL; CA1; CA12; CA13; CA14; CA2; CA3; CA4; CA5A; CA5B; CA6; CA7; CA9; CACNA1B; CALM1; CBR1; CBS; CCR4; CELA1; CLK1; CLK2; CLK3; CLK4; CREB1; CYP19A1; CYP1A1; CYP1B1; CYP2C8; DAPK1; DRD1; DRD5; DYRK1A; DYRK1B; DYRK2; EED; EGFR; ELAVL1; ELAVL3; ERAP1; ESR1; ESR2; G6PD; GALR3; GLO1; GPR35; GRK6; HSD17B1; HSD17B2; HSD17B3; IGF1R; IL2; KCND3; KDM4E; KDR; KLK7; LDHA; MAOA; MAOB; MCL1; MDH1; MET; MGAM; MIF; MPG; NEK6; NOX4; NQO1; OPRD1; P4HB; PDK3; PDK4; PGAM1; PGF; PIM1; PKLR; PLK1; PTGS1; PTGS2; PTPN1; PTPRS; RELA; SLC16A3; TAS2R31; TERT; TOP2A; TYR; VEGFA; XDH
BATMAN-TCM	0	None retained at the stated threshold
SEA	63	ABCB1; ABCC1; ABCG2; ACVRL1; AKR1B1; AKR1B10; ALDH2; ALOX12; ALOX15; ALOX5; ALPI; AMY1A; ANTXR2; APP; CA12; CA13; CA14; CA3; CA4; CA5A; CA7; CALM1; CBR1; CBS; CCR4; CELA1; CREB1; CYP1A1; CYP1B1; CYP2C8; DAPK1; ELAVL1; ELAVL3; ERAP1; ESR1; ESR2; GLO1; GPR35; GRK6; IL2; KCND3; KDM4E; KLK7; MAOA; MAOB; MDH1; MPG; NEK6; NOX4; NQO1; P4HB; PDK3; PDK4; PGF; PTPRS; RELA; SLC16A3; TAS2R31; TERT; TOP2A; TYR; VEGFA; XDH
TargetNet	27	ADRA2C; AHR; ALOX15; AR; CA12; CA14; CA4; CA5A; CA5B; CA6; CA7; CACNA1B; CYP19A1; DRD1; DRD5; ESR1; ESR2; GALR3; HSD17B1; HSD17B2; HSD17B3; MAOA; MAOB; MIF; OPRD1; PTGS1; RELA
SwissTargetPrediction	45	ACHE; ALK; ALOX5; ATG4B; AURKB; AXL; CA1; CA12; CA13; CA14; CA2; CA4; CA5A; CA5B; CA6; CA7; CA9; CBR1; CLK1; CLK2; CLK3; CLK4; DYRK1A; DYRK1B; DYRK2; EED; EGFR; ESR1; ESR2; G6PD; IGF1R; KDR; LDHA; MAOA; MAOB; MCL1; MET; MGAM; PGAM1; PIM1; PKLR; PLK1; PTGS2; PTPN1; TYR

2. Emodin (PubChem CID 3220)

Target set	n	Gene symbols
Merged unique	169	ABCB1; ABCB11; ACLY; AHR; AKR1B1; AKT1; ALKBH2; ALKBH3; ALKBH5; ALOX15; ALOX5; ALPL; ALPP; ANTXR2; APP; AQP1; AQP5; AR; ASRGL1; ATF4; ATHS; ATRNL1; BAX; BCL2; BCL2A1; BCL2L1; BGLAP; BTK; CA12; CA13; CA14; CA4; CA5A; CA5B; CA6; CA7; CA9; CASP3; CASP9; CCL27; CDC25B; CDH1; CDK2; CES1; CES2; CLDN5; CSF2; CSNK2A1; CSNK2A2; CSNK2B; CTDSP1; CTNNB1; CYP19A1; CYP1A1; CYP1A2; DDIT3; DNMT1; DRD1; DUSP3; EDN1; EGF; EGR1; ELANE; ESR1; ESR2; FASLG; FASN; FLT1; FLT4; FOS; FOXD3; FTO; FXR1; GPR35; HDAC1; HDAC6; HIF1A; HNF4A; HSD11B1; HSD17B1; HSD17B2; HSD17B3; HSP90AA1; HSPA5; HTR1E; IL1B; JAK1; JAK2; KDR; KIT; KRT12; KRT2; LCK; LDHA; LDHB; LIMK1; LIMK2; MAOA; MAOB; MAPK1; MAPK3; MAPK8IP1; MCL1; MIF; MIR145; MIR371A; MMP1; MMP9; MTA3; MTOR; MYC; NAT10; NFATC1; NFKB1; NOS2; NR2F2; OCLN; PARP1; PDLIM3; PGAM1; PIM1; PLA2G1B; PLA2G2E; PLEC; PLIN1; PLIN5; PPARG; PPP1R14A; PRKAA1; PRKAA2; PRKCD; PSMB1; PSMB5; PTGES; PTGS1; PTGS2; PTP4A3; PTPN1; PTPN7; PTPRC; RELA; RIPK2; ROS1; RUNX2; SARS; SHBG; SIRT1; SLC2A1; SLC2A4; SLPI; SMAD3; SOAT1; SOAT2; SRC; STAT3; STS; SYNJ2; TGFB1; TGFB2; TLR4; TLR9; TNF; TNFSF10; TNFSF11; TP53; TTR; TWIST1; TYR; VIM
BATMAN-TCM	94	ABCB1; ABCB11; AKT1; ALPL; ALPP; AQP1; AQP5; AR; ASRGL1; ATF4; ATHS; ATRNL1; BAX; BCL2; BGLAP; BTK; CASP3; CCL27; CDH1; CLDN5; CSF2; CSNK2A2; CTNNB1; CYP19A1; CYP1A1; CYP1A2; DDIT3; EDN1; EGF; EGR1; ESR1; ESR2; FASLG; FLT1; FLT4; FOS; FOXD3; FXR1; HIF1A; HSD11B1; HSP90AA1; HSPA5; IL1B; JAK1; JAK2; KDR; KIT; KRT12; KRT2; MAOB; MAPK1; MAPK3; MAPK8IP1; MIR145; MIR371A; MMP1; MMP9; MTA3; MTOR; MYC; NAT10; NFATC1; NFKB1; NOS2; OCLN; PDLIM3; PGAM1; PLA2G2E; PPARG; PPP1R14A; PRKAA1; PRKAA2; PRKCD; PTP4A3; ROS1; RUNX2; SARS; SHBG; SIRT1; SLC2A1; SLC2A4; SLPI; SMAD3; SRC; STAT3; TGFB1; TGFB2; TLR4; TNF; TNFSF10; TNFSF11; TP53; TWIST1; VIM
SEA	12	ACLY; AKR1B1; ANTXR2; BCL2A1; CSNK2A1; CSNK2B; ESR1; ESR2; FTO; PIM1; PTP4A3; TP53
TargetNet	49	AHR; AKR1B1; ALOX15; ALPL; AR; BCL2A1; CA12; CA13; CA14; CA4; CA5A; CA5B; CA6; CA7; CA9; CASP9; CDC25B; CDK2; CES1; CES2; CTDSP1; CYP1A2; DNMT1; DRD1; DUSP3; ESR1; ESR2; GPR35; HNF4A; HSD17B1; HSD17B2; HSD17B3; HTR1E; MAOA; MAOB; MCL1; MIF; NR2F2; PLA2G1B; PLIN1; PLIN5; PTGS1; PTGS2; PTPN7; PTPRC; RELA; RIPK2; STS; TLR9
SwissTargetPrediction	41	ACLY; AKR1B1; ALKBH2; ALKBH3; ALKBH5; ALOX5; APP; BCL2; BCL2L1; CSNK2A1; CYP19A1; ELANE; ESR1; ESR2; FASN; FTO; HDAC1; HDAC6; HSD17B1; HSD17B2; LCK; LDHA; LDHB; LIMK1; LIMK2; MAOA; MAOB; MCL1; PARP1; PIM1; PLEC; PSMB1; PSMB5; PTGES; PTP4A3; PTPN1; SOAT1; SOAT2; SYNJ2; TTR; TYR

3. Bellidifolin (PubChem CID 5281623)

Target set	n	Gene symbols
Merged unique	95	ABCB1; ABCG2; ACHE; AHR; AKR1B1; AKR1B10; ALOX15; ALOX5; ALPL; AR; BCL2A1; CA1; CA12; CA13; CA14; CA2; CA4; CA5A; CA5B; CA6; CA7; CA9; CACNA1B; CASP9; CDC25A; CDC25B; CES1; CES2; CLK1; CTDSP1; CYP17A1; CYP19A1; CYP1A2; CYP8B1; DNMT1; DUSP3; ENPP2; EPHX2; ESR1; ESR2; F10; G6PD; GPBAR1; GPR35; GRIN2A; HNF4A; HSD11B1; HSD11B2; HSD17B1; HSD17B2; HSD17B3; HSP90AA1; HTR1E; IDO1; MAOA; MAOB; MIF; NPC1L1; NR1H3; NR1H4; NR1I3; NR2E3; NR2F2; NR3C1; NR3C2; OSBP; OSBP2; PDE7A; PGR; PIM1; PLA2G1B; PLIN5; POLA1; PTGS1; PTGS2; PTPN1; PTPN2; PTPN22; PTPN7; RAC1; RELA; RIPK2; RORA; S1PR1; S1PR4; SERPINA6; SHBG; SLC10A1; STS; TERT; THRA; TLR9; TRPM8; UGT2B7; VDR
BATMAN-TCM	0	None retained at the stated threshold
SEA	0	None retained at the stated threshold
TargetNet	60	ABCB1; ABCG2; ACHE; AHR; AKR1B1; ALOX15; ALOX5; ALPL; BCL2A1; CA12; CA13; CA14; CA4; CA5A; CA5B; CA6; CA7; CA9; CACNA1B; CASP9; CES2; CLK1; CTDSP1; CYP17A1; CYP19A1; CYP1A2; DNMT1; DUSP3; ESR1; ESR2; GPR35; HNF4A; HSD17B1; HSD17B2; HSD17B3; HSP90AA1; HTR1E; MAOA; MAOB; MIF; NR2E3; NR2F2; PDE7A; PIM1; PLA2G1B; PLIN5; PTGS1; PTGS2; PTPN22; PTPN7; RAC1; RELA; RIPK2; RORA; S1PR1; S1PR4; STS; TERT; THRA; TLR9
SwissTargetPrediction	43	AKR1B1; AKR1B10; AR; CA1; CA2; CA4; CDC25A; CDC25B; CES1; CES2; CYP19A1; CYP8B1; ENPP2; EPHX2; ESR1; ESR2; F10; G6PD; GPBAR1; GRIN2A; HSD11B1; HSD11B2; HSD17B3; IDO1; NPC1L1; NR1H3; NR1H4; NR1I3; NR3C1; NR3C2; OSBP; OSBP2; PGR; POLA1; PTPN1; PTPN2; RELA; SERPINA6; SHBG; SLC10A1; TRPM8; UGT2B7; VDR

4. Cedrol (PubChem CID 65575)

Target set	n	Gene symbols
Merged unique	137	ABCB1; ABCC1; ABCG2; ACHE; ADAMTS4; ADAMTS5; ADORA3; AKT1; ALOX15; ALOX5; ALPL; APOBEC3A; APP; AR; AXL; BACE1; BCL2; CA1; CA12; CA2; CA3; CA4; CA5A; CA5B; CA6; CA7; CA9; CACNA1A; CACNA1C; CACNA1D; CACNA1F; CACNA1H; CACNA1S; CACNB1; CACNB2; CACNB3; CACNB4; CASP9; CBR1; CBS; CDC25B; CDK2; CES2; CHRM1; CHRM2; CHRM4; CLK1; CLK2; CLK3; CLK4; CSNK1E; CYP17A1; CYP19A1; CYP1A2; CYP1B1; DNMT1; DPP4; DYRK1A; DYRK1B; DYRK2; DYRK3; DYRK4; EGFR; ELAVL3; ERBB2; ESR1; ESR2; FGFR1; FLT3; FUT4; FUT7; FYN; GSK3B; HDAC6; HIF1A; HSD11B1; HSD11B2; HSD17B1; HTR1E; HTR5A; KCNH2; KDR; KIT; KLK1; KLK2; MAOB; MAPK14; MAPKAPK5; MAPT; MET; MGLL; MMP1; MMP13; MMP14; MMP2; MMP9; MTOR; NOS3; NR2F2; OPRK1; PGAM1; PGD; PGF; PIK3CA; PKM; PLK1; PRKAA2; PSMB5; PTGES; PTGS1; PTPN1; RAD52; RIPK2; RPS6KA3; S1PR2; SERPINE1; SHBG; SIRT6; SLC6A3; SNCA; SOST; SRC; SRD5A1; SRD5A2; ST3GAL3; STAT1; SYNJ2; TAS2R31; TERT; TLR9; TRPA1; TRPM8; TRPV3; TTR; TYR; VEGFA; XDH

Target set	n	Gene symbols
BATMAN-TCM	13	CACNA1A; CACNA1C; CACNA1D; CACNA1F; CACNA1S; CACNB1; CACNB2; CACNB3; CACNB4; OPRK1; TRPA1; TRPM8; TRPV3
SEA	16	CA3; CBS; CYP1B1; ELAVL3; ESR1; ESR2; FUT4; FUT7; KLK2; PGAM1; PGD; PGF; PTGS1; ST3GAL3; TAS2R31; VEGFA
TargetNet	29	ALOX15; ALPL; APOBEC3A; AR; CA5A; CA5B; CA6; CASP9; CDC25B; CES2; CHRM1; CHRM2; CHRM4; CYP17A1; CYP1A2; DYRK1A; HSD11B1; HSD11B2; HTR1E; HTR5A; MGLL; NOS3; NR2F2; RIPK2; S1PR2; SLC6A3; SRD5A1; SRD5A2; TLR9
SwissTargetPrediction	96	ABCB1; ABCC1; ABCG2; ACHE; ADAMTS4; ADAMTS5; ADORA3; AKT1; ALOX5; APP; AXL; BACE1; BCL2; CA1; CA12; CA2; CA3; CA4; CA5A; CA5B; CA6; CA7; CA9; CACNA1H; CBR1; CBS; CDK2; CES2; CLK1; CLK2; CLK3; CLK4; CSNK1E; CYP19A1; CYP1B1; DNMT1; DPP4; DYRK1A; DYRK1B; DYRK2; DYRK3; DYRK4; EGFR; ELAVL3; ERBB2; ESR1; ESR2; FGFR1; FLT3; FYN; GSK3B; HDAC6; HIF1A; HSD17B1; KCNH2; KDR; KIT; KLK1; KLK2; MAOB; MAPK14; MAPKAPK5; MAPT; MET; MMP1; MMP13; MMP14; MMP2; MMP9; MTOR; PGAM1; PGF; PIK3CA; PKM; PLK1; PRKAA2; PSMB5; PTGES; PTGS1; PTPN1; RAD52; RPS6KA3; SERPINE1; SHBG; SIRT6; SNCA; SOST; SRC; STAT1; SYNJ2; TAS2R31; TERT; TTR; TYR; VEGFA; XDH

5. Palmitic Acid (PubChem CID 985)

Target set	n	Gene symbols
Merged unique	416	A1BG; A2M; ABCB1; ABCC1; ABCG2; ABHD15; ACADSB; ACE; ACHE; ACLY; ADH1C; ADH5; ADORA3; AGT; AHSG; AHSP; AKR1B1; AKR1C2; AKR1C3; AKR1D1; ALDH5A1; ALDOA; ALOX12; ALOX15; ALOX5; ANPEP; APCS; APLP1; APLP2; APOA1; APOA2; APOA4; APOBEC3A; APOBR; APOE; APOL1; APOM; APP; AR; ASPA; ATP1A1; AXL; BACE1; BCHE; BCL2; BDKRB1; BRCC3; C1QB; C1QC; C1R; C1S; C3; C4A; C4B; C4BPA; C4BPB; C4B_2; C5; C8A; C8B; C8G; CA1; CA12; CA13; CA14; CA2; CA3; CA4; CA5A; CA5B; CA6; CA7; CA9; CACNA1H; CBR1; CBS; CCS; CD274; CDC25B; CDKN1A; CES1; CES2; CFB; CFH; CFI; CHRFAM7A; CHRM4; CHRM5; CHRNA7; CLEC4E; CLK2; CLU; CNR2; COX1; COX2; COX3; COX4I1; COX5A; COX5B; COX6A2; COX6B1; COX6C; COX7A1; COX7B; COX7C; COX8A; CP; CPN1; CPN2; CRTC2; CSNK2A1; CTSG; CYP19A1; CYP1B1; CYP2A13; CYP2A6; CYP2A7; CYP2B6; CYP2C18; CYP2C19; CYP2C8; CYP2C9; CYP2E1; CYP2F1; CYP2S1; DAND5; DCD; DPP4; DSP; DUSP3; EEF1A1; EEF1A2; EFTUD1; EGLN1; ELAVL3; ENO1; EPHX2; ESR1; ESR2; ESRRG; F12; F13B; F2; FAAH; FABP12; FABP3; FABP4; FABP5; FABP6; FABP7; FABP9; FCN3; FDPS; FECH; FEN1; FFAR1; FGA; FGFR1; FLT3; FN1; FOLH1; FTH1; FUT4; FUT7; FXN; GABRA1; GABRA2; GABRA3; GABRA4; GABRA5; GABRA6; GABRB1; GABRB2; GABRB3; GABRD; GABRE; GABRG1; GABRG2; GABRG3; GABRP; GABRQ; GALR3; GAPDHS; GLRA1; GLTP; GM2A; GPBAR1; GPHN; GPR35; GRIN1; GRIN2A; GRIN2B; GRIN2C; GRIN2D; GRIN3A; GRIN3B; GSN; GSTA1; GSTA2; GSTM1; GSTM2; GSTM3; GSTM4; GSTM5; GSTP1; GSTT1; GSTT2; GSTT2B; GUCA2A; HBA1; HBA2; HBB; HBD; HCAR2; HDAC1; HDAC10; HDAC11; HDAC2; HDAC3; HDAC4; HDAC5; HDAC6; HDAC7; HDAC8; HDAC9; HGF; HNF4A; HNF4G; HPGDS; HPR; HRNR; HSD17B1; IDE; IGFALS; IL10; IL1A; IL3; INS; ITGA2B; ITIH1; ITIH2; ITIH3; ITIH4; JCHAIN; JUP; KDR; KIT; KLK1; KLK2; KLKB1; KNG1; KRT1; KRT10; KRT14; KRT16; KRT2; KRT5; KRT6A; KRT6B; KRT6C; KRT9; LALBA; LTB4R; LTF; LY96; MAOB; MDM2; MET; MGLL; MGMT; MMP12; MMP13; MMP2; MMP9; MPG; MT1A; MT1B; MT1E; MT1F; MT1G; MT1H; MT1X; MT2A; MT3; NEIL1; NEIL2; NME1; NR1H4; NR1I2; NR1I3; ODC1; OGDH; OPN1LW; OPN1MW; OPN1MW2; OPN1MW3; OPN1SW; ORM1; ORM2; P4HB; PAEP; PARP1; PCYT1A; PCYT1B; PDCD6; PDE4D; PDIA3; PGAM1; PGD; PGF; PGLYRP2; PHOSPHO1; PKM; PLA2G10; PLA2G1B; PLA2G2A; PLA2G2D; PLA2G5; PLD1; PLD2; PLIN1; PLK1; PMP2; POLB; PON1; PPARA; PPARD; PPARG; PPT1; PRDX1; PSMB5; PSPH; PTEN; PTGER2; PTGER4; PTGES; PTGFR; PTGIR; PTGS1; PTGS2; PTPN1; PTPRC; RAD52; RHO; RORA; RPS6KA3; S100A2; S100A7; S100A7A; S100A8; S100A9; S1PR1; S1PR2; S1PR3; S1PR4; S1PR5; SCN10A; SCN11A; SCN1A; SCN1B; SCN2A; SCN2B; SCN3A; SCN3B; SCN4A; SCN4B; SCN5A; SCN7A; SCN8A; SCN9A; SEMG1; SEPP1; SERPINA1; SERPINA3; SERPINA4; SERPINA6; SERPIND1; SERPINE1; SHBG; SIRT2; SIVA1; SNCA; SOAT1; SOD1; SOST; SRC; SRD5A2; ST3GAL3; SUCNR1; SYNJ2; TAS2R31; TBXA2R; TBXAS1; TF; TFRC; THRA; THRB; TLR2; TLR4; TLR9; TNF; TP53; TP73; TPI1; TRAPPC3; TRAPPC3L; TTR; TUFM; TYR; UTRN; VDR; VEGFA; VLDLR; VTN; XDH; YWHAZ
BATMAN-TCM	306	A1BG; A2M; ABHD15; ACADSB; ACHE; ACLY; ADH1C; ADH5; AGT; AHSG; AHSP; AKR1C2; AKR1D1; ALDH5A1; ALDOA; APCS; APLP1; APLP2; APOA1; APOA2; APOA4; APOBR; APOE; APOL1; APOM; APP; ASPA; ATP1A1; BCHE; BCL2; BDKRB1; BRCC3; C1QB; C1QC; C1R; C1S; C3; C4A; C4B; C4BPA; C4BPB; C4B_2; C5; C8A; C8B; C8G; CCS; CD274; CDKN1A; CES1; CFB; CFH; CFI; CHRFAM7A; CHRNA7; CLEC4E; CLU; COX1; COX2; COX3; COX4I1; COX5A; COX5B; COX6A2; COX6B1; COX6C; COX7A1; COX7B; COX7C; COX8A; CP; CPN1; CPN2; CRTC2; CYP2A13; CYP2A6; CYP2A7; CYP2B6; CYP2C18; CYP2C19; CYP2C8; CYP2C9; CYP2E1; CYP2F1; CYP2S1; DAND5; DCD; DSP; EEF1A1; EEF1A2; EFTUD1; EGLN1; ENO1; ESR1; ESRRG; F12; F13B; F2; FABP12; FABP3; FABP4; FABP5; FABP6; FABP7; FABP9; FCN3; FECH; FEN1; FGA; FN1; FTH1; FXN; GABRA1; GABRA2; GABRA3; GABRA4; GABRA5; GABRA6; GABRB1; GABRB2; GABRB3; GABRD; GABRE; GABRG1; GABRG2; GABRG3; GABRP; GABRQ; GAPDHS; GLRA1; GLTP; GM2A; GPBAR1; GPHN; GRIN1; GRIN2A; GRIN2B; GRIN2C; GRIN2D; GRIN3A; GRIN3B; GSN; GSTA1; GSTA2; GSTM1; GSTM2; GSTM3; GSTM4; GSTM5; GSTP1; GSTT1; GSTT2; GSTT2B; GUCA2A; HBA1; HBA2; HBB; HBD; HDAC1; HDAC10; HDAC11; HDAC2; HDAC3; HDAC4; HDAC5; HDAC6; HDAC7; HDAC8; HDAC9; HGF; HNF4A; HNF4G; HPGDS; HPR; HRNR; IDE; IGFALS; IL10; IL1A; IL3; INS; ITIH1; ITIH2; ITIH3; ITIH4; JCHAIN; JUP; KLK1; KLKB1; KNG1; KRT1; KRT10; KRT14; KRT16; KRT2; KRT5; KRT6A; KRT6B; KRT6C; KRT9; LALBA; LTF; LY96; MDM2; MGMT; MMP9; MPG; MT1A; MT1B; MT1E; MT1F; MT1G; MT1H; MT1X; MT2A; MT3; NEIL1; NEIL2; NME1; NR1H4; NR1I2; NR1I3; ODC1; OGDH; OPN1LW; OPN1MW; OPN1MW2; OPN1MW3; OPN1SW; ORM1; ORM2; P4HB; PAEP; PARP1; PCYT1A; PCYT1B; PDCD6; PDIA3; PGLYRP2; PHOSPHO1; PLA2G1B; PLA2G2A; PLA2G2D; PLD1; PLD2; PMP2; POLB; PON1; PPARA; PPARD; PPARG; PPT1; PRDX1; PSPH; PTEN; RHO; RORA; S100A2; S100A7; S100A7A; S100A8; S100A9; SCN10A; SCN11A; SCN1A; SCN1B; SCN2A; SCN2B; SCN3A; SCN3B; SCN4A; SCN4B; SCN5A; SCN7A; SCN8A; SCN9A; SEMG1; SEPP1; SERPINA1; SERPINA3; SERPINA4; SERPINA6; SERPIND1; SHBG; SIVA1; SOD1; SRD5A2; SUCNR1; TF; TFRC; TLR2; TLR4; TNF; TP53; TP73; TPI1; TRAPPC3; TRAPPC3L; TTR; TUFM; TYR; UTRN; VDR; VLDLR; VTN; YWHAZ
SEA	15	CA3; CBS; CYP1B1; ELAVL3; ESR1; ESR2; FUT4; FUT7; KLK2; PGAM1; PGD; PGF; ST3GAL3; TAS2R31; VEGFA
TargetNet	61	ACE; ALOX5; ANPEP; APOBEC3A; CA12; CA13; CA14; CA2; CA4; CA5A; CA5B; CA6; CA7; CDC25B; CES1; CES2; CHRM4; CHRM5; CNR2; CSNK2A1; CTSG; DUSP3; EPHX2; FAAH; FDPS; FFAR1; FOLH1; GALR3; GPR35; HCAR2; HDAC1; HDAC2; HDAC3; HDAC4; HDAC6; HDAC8; ITGA2B; LTB4R; MGLL; PLA2G1B; PLA2G2A; PLIN1; PTGER2; PTGER4; PTGES; PTGFR; PTGIR; PTPN1; PTPRC; S1PR1; S1PR2; S1PR3; S1PR4; S1PR5; SIRT2; SOAT1; TBXA2R; TBXAS1; THRA; THRB; TLR9
SwissTargetPrediction	73	ABCB1; ABCC1; ABCG2; ACHE; ADORA3; AKR1B1; AKR1C3; ALOX12; ALOX15; APP; AR; AXL; BACE1; BCHE; BCL2; CA1; CA12; CA2; CA3; CA4; CA5A; CA5B; CA6; CA7; CA9; CACNA1H; CBR1; CBS; CLK2; CYP19A1; CYP1B1; DPP4; ESR1; ESR2; FGFR1; FLT3; HDAC6; HSD17B1; KDR; KIT; KLK1; KLK2; MAOB; MET; MMP12; MMP13; MMP2; MMP9; PDE4D; PGF; PKM; PLA2G10; PLA2G2A; PLA2G5; PLK1; PPARG; PSMB5; PTGS1; PTGS2; PTPN1; RAD52; RPS6KA3; SERPINE1; SHBG; SNCA; SOST; SRC; SYNJ2; TAS2R31; TTR; TYR; VEGFA; XDH

6. Aromadendrin (PubChem CID 122850)

Target set	n	Gene symbols
Merged unique	195	ABCG2; ACE; ACER2; ACLY; ACP1; ADH1A; ADH1B; ADH7; ADRA2C; AHR; AKR1B1; AKR1B10; AKR1C3; ALOX15; APLNR; AR; ATG4B; BBOX1; BCL2A1; BHMT; CA1; CA12; CA13; CA14; CA2; CA4; CA5A; CA5B; CA6; CA7; CACNA2D1; CACNA2D2; CASP9; CDC25A; CDC25B; CDC25C; CDC45; CERT1; CES1; CES2; CISD1; CPT2; CYP19A1; CYP4A11; CYP4F2; DAGLA; DNM1; DNMT1; DUSP3; ENPEP; ENPP2; EPHX1; EPHX2; ESR1; ESR2; F10; F3; FAAH; FABP1; FABP2; FABP3; FABP4; FABP5; FDPS; FFAR1; FFAR4; FOLH1; G6PD; GABBR1; GABBR2; GABRQ; GABRR1; GBA; GGPS1; GNAI1; GNAI3; GNAO1; GPBAR1; GPR174; GPR18; GPR34; GPR35; GPR84; GRM5; GSR; GSTA1; GSTK1; GSTM1; HAO1; HCAR2; HDAC3; HMGCR; HNF4A; HSD11B1; HSD11B2; HSD17B1; HSD17B2; HSD17B3; IDO1; KDM2A; KDM4A; KDM4C; KDM4E; KDM5A; KDM5C; KDM7A; LAP3; LPAR1; LPAR2; LPAR3; LPAR4; LPAR6; LTA4H; LTB4R; MAOA; MAOB; MIF; MMP2; MPEG1; NAAA; NOD1; NPC1L1; NR1H4; NR3C1; NR3C2; NT5E; OXER1; P2RY10; PAM; PAOX; PGR; PHF8; PLA2G10; PLA2G1B; PLA2G2A; PLA2G2C; PLA2G4B; PLA2G5; PLG; POLB; POLH; POLK; POLL; POLM; PPARA; PPARD; PPARG; PRKCA; PTGER2; PTGER3; PTGER4; PTGES; PTGFR; PTGIR; PTGS1; PTGS2; PTPN1; PTPN2; PTPN7; PTPRC; RARB; RELA; RIPK2; RORC; S1PR1; S1PR2; S1PR3; S1PR4; S1PR5; SCD; SELP; SERPINA6; SHBG; SLC22A1; SLC22A6; SLC22A8; SLC25A20; SLC6A11; SLCO2A1; SPHK1; SPTLC2; STS; TBXA2R; TBXAS1; TERT; THRA; THRB; TLR2; TLR4; TLR9; TRPV1; UGT2B7; VDR; ZDHHC2; ZDHHC20
BATMAN-TCM	0	None retained at the stated threshold
SEA	111	ACER2; ACP1; ADH1A; ADH1B; ADH7; ATG4B; BBOX1; BHMT; CDC25A; CDC25B; CDC25C; CERT1; CES1; CES2; CPT2; CYP4F2; DAGLA; DNM1; ENPEP; ENPP2; EPHX1; EPHX2; FAAH; FABP3; FABP4; FDPS; FFAR4; FOLH1; GABBR1; GABBR2; GABRQ; GABRR1; GBA; GGPS1; GNAI1; GNAI3; GNAO1; GPR174; GPR18; GPR34; GPR84; GSR; GSTA1; GSTK1; GSTM1; HAO1; HMGCR; HSD17B3; KDM2A; KDM4A; KDM4C; KDM4E; KDM5A; KDM5C; KDM7A; LAP3; LPAR1; LPAR2; LPAR3; LPAR4; LPAR6; LTB4R; MPEG1; NAAA; NOD1; OXER1; P2RY10; PAM; PAOX; PHF8; PLA2G10; PLA2G1B; PLA2G2A; PLA2G2C; PLA2G4B; PLA2G5; POLB; POLH; POLK; POLL; POLM; PPARA; PPARD; PPARG; PRKCA; PTGER2; PTGER3; PTGER4; PTGFR; PTGIR; RARB; S1PR1; S1PR2; S1PR3; S1PR4; S1PR5; SELP; SLC22A1; SLC22A6; SLC22A8; SLC25A20; SLC6A11; SLCO2A1; SPHK1; SPTLC2; TBXA2R; TBXAS1; THRA; THRB; TLR2; TRPV1
TargetNet	38	ABCG2; ADRA2C; AHR; AKR1B1; ALOX15; BCL2A1; CA12; CA13; CA14; CA4; CA5A; CA5B; CA6; CA7; CASP9; CES2; CYP19A1; DNMT1; DUSP3; ESR1; ESR2; GPR35; HCAR2; HNF4A; HSD17B1; HSD17B2; HSD17B3; MAOA; MAOB; MIF; PTGS1; PTPN1; PTPN7; RELA; RIPK2; STS; TERT; TLR9
SwissTargetPrediction	79	ACE; ACLY; AKR1B1; AKR1B10; AKR1C3; APLNR; AR; CA1; CA2; CACNA2D1; CACNA2D2; CDC25A; CDC25B; CDC45; CES1; CES2; CISD1; CYP19A1; CYP4A11; CYP4F2; ENPP2; ESR1; ESR2; F10; F3; FAAH; FABP1; FABP2; FABP3; FABP4; FABP5; FFAR1; FOLH1; G6PD; GPBAR1; GPR84; GRM5; GSTK1; HAO1; HDAC3; HMGCR; HSD11B1; HSD11B2; HSD17B3; IDO1; KDM2A; KDM5C; LTA4H; MMP2; NPC1L1; NR1H4; NR3C1; NR3C2; NT5E; PGR; PHF8; PLG; PPARA; PPARD; PPARG; PTGER2; PTGES; PTGFR; PTGS1; PTGS2; PTPN1; PTPN2; PTPRC; RELA; RORC; SCD; SERPINA6; SHBG; TERT; TLR4; UGT2B7; VDR;

Target set	n	Gene symbols
		ZDHHC2; ZDHHC20

7. Kaempferol (PubChem CID 5280863)

Target set	n	Gene symbols
Merged unique	201	ABCB1; ABCC1; ABCG2; ACHE; ACP1; ADORA3; AHR; AKR1B1; AKR1B10; AKT1; ALDH2; ALK; ALKBH5; ALOX12; ALOX15; ALOX5; ALPI; ALPL; AMY1A; ANTXR2; APP; AURKB; AXL; BACE1; BAX; BCHE; BCL2; BCL2A1; BIRC2; BIRC3; BMPR2; BTK; CA1; CA12; CA13; CA14; CA2; CA3; CA4; CA5A; CA5B; CA6; CA7; CA9; CASP3; CAT; CBR1; CBS; CCR4; CDC25B; CDK1; CDK2; CDK6; CES1; CES2; CISD1; CLDN2; CREB1; CSNK2A1; CTDSP1; CTNNB1; CXCL8; CYP17A1; CYP19A1; CYP1A1; CYP1A2; CYP1B1; CYP2C8; CYP3A4; DAPK1; DIO1; DNMT1; DPP4; DUSP3; EGFR; ELAVL1; ELAVL3; ESR1; ESR2; ESRRA; ESRRB; FBP1; FLT3; FOXP3; FTO; GLO1; GPER1; GPR35; GRK6; GSK3A; GSK3B; GSTM1; GSTM2; GSTO1; HDAC6; HMOX1; HNF4A; HSD17B1; HSD17B2; HSD17B3; HSP90AA1; HTR1E; ICAM1; IGF1R; IL2; IP6K2; IPMK; ITGB3; ITPR3; JUN; KCND3; KDM4E; KDR; LYN; MAOA; MAOB; MAPK14; MAPK8; MAPT; MCL1; MCU; MET; MIF; MIR21; MKNK2; MMP1; MMP2; MMP9; MPG; MPO; MYLK; MYLK3; NEK2; NEK6; NFE2L2; NFKB1; NFKBIA; NMUR2; NOS2; NOX4; NQO1; NR1I2; NR1I3; NSFL1C; NUAK1; P4HB; PARK7; PDE7A; PGF; PIK3CA; PIM1; PKM; PKN1; PLA2G1B; PLEK; PLK1; PPARG; PSMB5; PTGS1; PTGS2; PTK2; PTPN1; PTPN22; PTPRC; PTPRS; RELA; RIPK2; RPS6KA1; RPS6KA2; RPS6KA3; SELE; SETD7; SIRT6; SLC11A2; SLC16A1; SLC22A12; SLC2A1; SLCO1B1; SLCO2B1; SNCA; SRC; ST6GAL1; STAT1; STAT3; STS; SYK; TERT; TJP1; TLR9; TNF; TNKS; TNKS2; TOP2A; TRPC5; TTR; TXN; TYR; VCAM1; VEGFA; XDH; YES1
BATMAN-TCM	62	AHR; AKT1; BAX; BCL2; BIRC2; BIRC3; BMPR2; BTK; CASP3; CAT; CDK1; CDK2; CLDN2; CXCL8; CYP1A1; CYP1A2; CYP1B1; CYP3A4; DIO1; ESRRA; FOXP3; GPER1; GSTM1; GSTM2; HMOX1; HSP90AA1; ICAM1; ITGB3; ITPR3; JUN; LYN; MAPK14; MAPK8; MCU; MIR21; MMP1; MMP9; MYLK3; NFE2L2; NFKB1; NFKBIA; NOS2; NOX4; NR1I2; NR1I3; NSFL1C; PARK7; PLEK; PPARG; PTGS2; RPS6KA3; SELE; SLC11A2; SLCO1B1; STAT1; STAT3; SYK; TJP1; TNF; TXN; VCAM1; XDH
SEA	62	ABCB1; ABCC1; ABCG2; ACP1; AKR1B1; AKR1B10; ALDH2; ALOX12; ALOX15; ALOX5; ALPI; AMY1A; ANTXR2; CA12; CA3; CA4; CA7; CBR1; CBS; CCR4; CREB1; CYP1A1; CYP1B1; CYP2C8; DAPK1; ELAVL1; ELAVL3; ESR1; ESR2; ESRRB; GLO1; GPR35; GRK6; GSTO1; HSD17B1; HSD17B2; HSD17B3; IL2; KCND3; KDM4E; MAOA; MAPT; MPG; MPO; MYLK; NEK6; NMUR2; NOX4; NQO1; NUAK1; P4HB; PGF; PKN1; PTPN1; PTPRS; RPS6KA3; ST6GAL1; TERT; TOP2A; TTR; VEGFA; XDH
TargetNet	53	ABCB1; ABCG2; AHR; AKR1B1; ALOX15; ALOX5; ALPL; BCL2A1; CA12; CA13; CA14; CA4; CA5A; CA5B; CA6; CA7; CA9; CDC25B; CES1; CES2; CTDSP1; CYP17A1; CYP19A1; CYP1A2; DNMT1; DUSP3; ESR1; ESR2; GPR35; GSK3A; HNF4A; HSD17B1; HSD17B2; HSD17B3; HTR1E; MAOA; MAOB; MCL1; MIF; PDE7A; PIM1; PLA2G1B; PTGS1; PTGS2; PTPN1; PTPN22; PTPRC; RELA; RIPK2; RPS6KA3; STS; TERT; TLR9
SwissTargetPrediction	97	ABCB1; ABCC1; ABCG2; ACHE; ADORA3; AHR; AKR1B1; AKR1B10; AKT1; ALK; ALKBH5; ALOX12; ALOX15; ALOX5; APP; AURKB; AXL; BACE1; BCHE; CA1; CA12; CA2; CA3; CA4; CA5A; CA6; CA7; CA9; CDK2; CDK6; CISD1; CSNK2A1; CTNNB1; CYP19A1; CYP1B1; DAPK1; DPP4; EGFR; ELAVL3; ESR2; ESRRA; FBP1; FLT3; FTO; GLO1; GPR35; GSK3B; HDAC6; HSD17B1; HSD17B2; IGF1R; IP6K2; IPMK; KDR; MAOA; MAOB; MAPT; MET; MKNK2; MMP2; MMP9; MPG; MPO; NEK2; NEK6; NOX4; NUAK1; P4HB; PIK3CA; PIM1; PKM; PKN1; PLK1; PPARG; PSMB5; PTK2; PTPN1; PTPRS; RPS6KA1; RPS6KA2; RPS6KA3; SETD7; SIRT6; SLC16A1; SLC22A12; SLC2A1; SLCO2B1; SNCA; SRC; SYK; TNKS; TNKS2; TRPC5; TTR; TYR; XDH; YES1

8. Physcion (PubChem CID 10639)

Target set	n	Gene symbols
Merged unique	101	ABCG2; ACLY; ACVRL1; ADORA2A; AHR; AKR1B1; ALKBH2; ALKBH3; ALKBH5; ALOX15; ALPL; APP; AR; BAD; BCL2; BCL2A1; CA12; CA13; CA14; CA4; CA5A; CA5B; CA6; CA7; CA9; CASP9; CBR1; CBS; CDC25B; CDC42; CDK2; CES1; CES2; CNR2; CSNK2A1; CYP19A1; CYP1A2; CYP1B1; DNMT1; DRD1; DUSP3; ELANE; ELAVL3; ESR1; ESR2; FASN; FTO; GPR35; HDAC6; HNF4A; HSD17B1; HSD17B2; HSD17B3; HTR1E; KCNA3; LCK; LIMK1; LIMK2; MAOA; MAOB; MAP2K3; MAP2K6; MAP3K7; MCL1; MIF; NR2F2; PARP1; PDGFRA; PGD; PGR; PIM1; PLAA; PLIN1; PLIN5; PPARG; PSMB1; PSMB5; PTGS1; PTGS2; PTP4A3; PTPN1; PTPN7; RAC1; RELA; RIPK2; RORA; SHBG; SLC22A6; SLC22A8; SLC37A4; SOAT1; SOAT2; STS; SYK; SYNJ2; TAS2R31; THRA; THRB; TLR9; TNF; TTR
BATMAN-TCM	6	AR; ESR1; ESR2; PGD; PGR; SHBG
SEA	16	ABCG2; ACLY; ACVRL1; BCL2A1; CBR1; CBS; CYP1B1; ELANE; ELAVL3; MAP2K6; MAP3K7; PDGFRA; PLAA; PTP4A3; SLC37A4; TAS2R31
TargetNet	54	ABCG2; AHR; ALOX15; ALPL; BCL2A1; CA12; CA13; CA14; CA4; CA5A; CA5B; CA6; CA7; CA9; CASP9; CDC25B; CDC42; CDK2; CES1; CES2; CYP19A1; CYP1A2; DNMT1; DRD1; DUSP3; ESR1; ESR2; GPR35; HNF4A; HSD17B1; HSD17B2; HSD17B3; HTR1E; MAOA; MAOB; MCL1; MIF; NR2F2; PDGFRA; PLIN1; PLIN5; PTGS1; PTGS2; PTPN7; RAC1; RELA; RIPK2; RORA; STS; SYK; THRA; THRB; TLR9; TNF
SwissTargetPrediction	43	ACLY; ADORA2A; AKR1B1; ALKBH2; ALKBH3; ALKBH5; APP; BAD; BCL2; CDC25B; CNR2; CSNK2A1; CYP19A1; DUSP3; ELANE; ESR1; ESR2; FASN; FTO; HDAC6; KCNA3; LCK; LIMK1; LIMK2; MAOA; MAOB; MAP2K3; MCL1; PARP1; PIM1; PPARG; PSMB1; PSMB5; PTP4A3; PTPN1; SLC22A6; SLC22A8; SOAT1; SOAT2; SYNJ2; THRA; THRB; TTR

9. Quercetin (PubChem CID 5280343)

Target set	n	Gene symbols
Merged unique	314	ABCA1; ABCB1; ABCC1; ABCC2; ABCG2; ACE2; ACHE; ADAMTS4; ADIPOQ; ADORA3; AHR; AKR1B1; AKT1; ALOX12; ALOX15; ALOX5; ALPL; APOE; APP; AR; ATF4; AURKB; AXIN1; AXIN2; AXL; BACE1; BAX; BCL2; BCL2A1; BCL2L1; BDNF; BMP2; BRCA1; CA1; CA12; CA13; CA14; CA2; CA3; CA4; CA5A; CA5B; CA6; CA7; CA9; CASP3; CASP8; CASP9; CAT; CAV1; CBR1; CBS; CCL2; CCNB1; CCND1; CD274; CD40LG; CD86; CDC25B; CDH1; CDK1; CDK2; CDKN1A; CDKN2A; CEBPB; CES1; CES2; CHEK2; CHUK; CLDN4; CLDN5; CLK1; CLK2; COL1A1; COMT; CPT1A; CREB1; CRH; CRP; CRTC1; CSF2; CSF3; CSNK2A1; CTDSP1; CTNNB1; CTSD; CXCL10; CXCL2; CXCL8; CYP17A1; CYP19A1; CYP1A1; CYP1A2; CYP1B1; CYP2C19; CYP2C8; CYP2E1; CYP3A4; DNAJC5; DNMT1; DPP4; DUSP3; E2F1; E2F2; EGF; EGFR; EGR1; EIF2S1; ELAVL1; ELAVL3; ELK1; ERBB2; ERBB3; ERN2; ESR1; ESR2; ESRRA; ESRRB; F2; F3; FASN; FGFR1; FLT3; FOS; FOSL1; FUT4; FUT7; GAA; GABRR3; GCK; GK; GLO1; GPR35; GSK3A; GSK3B; GSTM1; GSTM2; GSTP1; HDAC6; HIF1A; HK2; HMOX1; HNRNPA1; HSD17B1; HSD17B2; HSD17B3; HSF1; HSP90AA1; HSP90B2P; HSPA4; HSPA5; HSPB1; HTR1E; HVCN1; ICAM1; IDO1; IFNG; IGFBP3; IL10; IL17A; IL1A; IL1B; IL2; IL6; INSR; IRF1; JAK2; JUN; KDM1A; KDR; KHK; KIT; KLK1; KLK2; LDLR; LEF1; LGALS1; LPCAT1; LPCAT2; LYN; MAOA; MAOB; MAP3K5; MAPK1; MAPK10; MAPK14; MAPK3; MAPK8; MAPT; MCL1; MECP2; MET; MIF; MLXIPL; MMP1; MMP12; MMP13; MMP2; MMP9; MPO; MRC1; MTOR; MUC5AC; MYC; NCF1; NDUFV2; NEK6; NFE2L2; NFKB1; NFKBIA; NGF; NKX3-1; NLRP3; NOS2; NOS3; NPEPPS; NQO1; NR1H3; NR1I2; NR1I3; NRF1; ODC1; P2RX4; PARP1; PGAM1; PGD; PGF; PGP; PIK3CA; PIK3CB; PIM1; PKM; PLA2G1B; PLAT; PLAU; PLK1; PON1; POR; PPARA; PPARG; PRKAA1; PRKAA2; PRKCA; PRKCB; PRKCD; PSMB5; PTEN; PTGS1; PTGS2; PTK2B; PTPN1; PTPN11; PTPN22; PTPRC; PTX3; QDPR; RAC1; RAD52; RAF1; RASSF1; RB1; RELA; RIPK2; RPS6KA3; RUNX2; SCD; SELE; SERPINE1; SHBG; SIRT1; SIRT6; SLC16A1; SLC23A1; SLC2A1; SLC2A4; SLPI; SNCA; SOST; SPP1; SRC; ST3GAL3; STAT1; STAT3; SULT1E1; SYNJ2; TAS2R31; TBK1; TERT; TGFB1; THBS1; TJP1; TLR9; TNF; TNFRSF10B; TOP1; TOP2A; TP53; TTR; TXN; TXNIP; TYR; UGT1A1; UGT1A3; UGT1A9; UQCRC1; USP10; VCAM1; VDR; VEGFA; VIM; VIM2P; VTN; XBP1; XDH
BATMAN-TCM	217	ABCA1; ABCG2; ACE2; ACHE; ADIPOQ; AHR; AKT1; APOE; AR; ATF4; AURKB; AXIN1; AXIN2; BAX; BCL2; BCL2L1; BDNF; BMP2; BRCA1; CASP3; CASP8; CASP9; CAT; CAV1; CCL2; CCNB1; CCND1; CD274; CD40LG; CD86; CDH1; CDK1; CDKN1A; CDKN2A; CEBPB; CHEK2; CHUK; CLDN4; CLDN5; COL1A1; COMT; CPT1A; CREB1; CRH; CRP; CRTC1; CSF2; CSF3; CTNNB1; CTSD; CXCL10; CXCL2; CXCL8; CYP1A1; CYP1A2; CYP1B1; CYP2C19; CYP2C8; CYP2E1; CYP3A4; DNAJC5; E2F1; E2F2; EGF; EGFR; EGR1; EIF2S1; ELK1; ERBB2; ERBB3; ERN2; ESRRA; ESRRB; F2; F3; FASN; FOS; FOSL1; GAA; GABRR3; GCK; GK; GSK3B; GSTM1; GSTM2; GSTP1; HIF1A; HK2; HMOX1; HNRNPA1; HSF1; HSP90AA1; HSP90B2P; HSPA4; HSPA5; HSPB1; HVCN1; ICAM1; IFNG; IGFBP3; IL10; IL17A; IL1A; IL1B; IL2; IL6; INSR; IRF1; JAK2; JUN; KDM1A; KHK; LDLR; LEF1; LGALS1; LPCAT1; LPCAT2; LYN; MAOA; MAP3K5; MAPK1; MAPK14; MAPK3; MAPK8; MCL1; MECP2; MLXIPL; MMP1; MMP2; MMP9; MPO; MRC1; MTOR; MUC5AC; MYC; NCF1; NDUFV2; NFE2L2; NFKB1; NFKBIA; NGF; NKX3-1; NLRP3; NOS2; NOS3; NPEPPS; NQO1; NR1H3; NR1I2; NR1I3; NRF1; ODC1; P2RX4; PARP1; PGP; PIK3CA; PIK3CB; PLAT; PLAU; PON1; POR; PPARA; PRKAA1; PRKAA2; PRKCA; PRKCB; PRKCD; PTEN; PTGS1; PTGS2; PTK2B; PTPN11; PTX3; RAF1; RASSF1; RB1; RELA; RUNX2; SCD; SELE; SERPINE1; SIRT1; SLC16A1; SLC23A1; SLC2A1; SLC2A4; SLPI; SNCA; SPP1; STAT1; STAT3; SULT1E1; TBK1; TGFB1; THBS1; TJP1; TNF; TNFRSF10B; TOP1; TOP2A; TP53; TXN; TXNIP; TYR; UGT1A1; UGT1A3; UGT1A9; UQCRC1; USP10; VCAM1; VDR; VEGFA; VIM; VIM2P; VTN; XBP1; XDH
SEA	26	ABCC2; CA12; CA3; CA4; CA6; CA7; CBS; CYP1B1; CYP2C8; ELAVL1; ELAVL3; ESR1; ESR2; FUT4; FUT7; GLO1; KLK2; MMP12; NEK6; PGAM1; PGD; PGF; QDPR; ST3GAL3; TAS2R31; VEGFA
TargetNet	55	ABCB1; ABCG2; AHR; AKR1B1; ALOX15; ALOX5; ALPL; BCL2A1; CA12; CA13; CA14; CA4; CA5A; CA5B; CA6; CA7; CA9; CDC25B; CES1; CES2; CSNK2A1; CTDSP1; CYP17A1; CYP19A1; CYP1A2; DNMT1; DUSP3; ESR1; ESR2; FLT3; GPR35; GSK3A; GSK3B; HSD17B1; HSD17B2; HSD17B3; HTR1E; MAOA; MAOB; MAPK10; MCL1; MIF; PIM1; PLA2G1B; PTGS1; PTGS2; PTPN1; PTPN22; PTPRC; RAC1; RELA; RIPK2; RPS6KA3; TERT; TLR9
SwissTargetPrediction	71	ABCB1; ABCC1; ABCG2; ACHE; ADAMTS4; ADORA3; AKR1B1; AKT1; ALOX12; ALOX15; ALOX5; APP; AXL; BACE1; BCL2; CA1; CA12; CA2; CA3; CA4; CA5A; CA5B; CA6; CA7; CA9; CBR1; CDK2; CLK1;

Target set	n	Gene symbols
		CLK2; CYP19A1; CYP1B1; DPP4; ELAVL3; ESR1; ESR2; FGFR1; FLT3; GSK3B; HDAC6; HSD17B1; IDO1; KDR; KIT; KLK1; KLK2; MAOB; MAPT; MET; MMP12; MMP13; MMP2; MMP9; PIK3CA; PKM; PLK1; PPARG; PSMB5; PTGS1; RAD52; RPS6KA3; SERPINE1; SHBG; SIRT6; SNCA; SOST; SRC; SYNJ2; TERT; TTR; TYR; XDH

10. Taxifolin (PubChem CID 439533)

Target set	n	Gene symbols
Merged unique	148	ABCB1; ABCC1; ABCG2; ACE; ACHE; ACP1; ADORA3; ADRA2C; AHR; AKR1B1; AKR1B10; AKT1; ALDH2; ALK; ALKBH5; ALOX12; ALOX15; ALOX5; ALPI; ALPL; AMY1A; ANTXR2; APOE; APP; AR; AURKB; AXL; BACE1; BCHE; BCL2A1; CA1; CA12; CA13; CA14; CA2; CA3; CA4; CA5A; CA5B; CA6; CA7; CA9; CBR1; CBS; CCR4; CDC25B; CDK2; CDK6; CES2; CISD1; CSNK2A1; CTDSP1; CTNNB1; CYP19A1; CYP1A1; CYP1B1; CYP2C8; DAPK1; DNMT1; DPP4; DUSP3; EGFR; ELAVL1; ELAVL3; ESR1; ESR2; ESRRA; ESRRB; FBP1; FLT3; FTO; GLO1; GPR35; GRK6; GSK3B; GSTO1; HDAC6; HNF4A; HSD11B2; HSD17B1; HSD17B2; HSD17B3; IGF1R; IP6K2; IPMK; KCND3; KDM4E; KDR; MAOA; MAOB; MAPT; MET; MIF; MKNK2; MMP2; MMP9; MPG; MPO; NEK2; NEK6; NMUR2; NOX4; NQO1; NUAK1; P4HB; PGF; PIK3CA; PIK3CB; PIM1; PKM; PKN1; PLIN5; PLK1; PPARG; PSMB5; PTGS1; PTK2; PTPN1; PTPN7; PTPRS; RELA; RIPK2; RPS6KA1; RPS6KA2; RPS6KA3; SETD7; SIRT6; SLC16A1; SLC22A12; SLC2A1; SLCO2B1; SNCA; SRC; SRD5A1; ST6GAL1; SYK; TERT; TLR9; TNKS; TNKS2; TOP2A; TREM2; TRPC5; TTR; TYR; VEGFA; XDH; YES1
BATMAN-TCM	11	ACE; AHR; APOE; CYP1A1; EGFR; HSD11B2; PIK3CB; SRC; SRD5A1; TOP2A; TREM2
SEA	57	ABCB1; ABCC1; ABCG2; ACP1; AKR1B1; AKR1B10; ALDH2; ALOX12; ALOX15; ALOX5; ALPI; AMY1A; ANTXR2; CA12; CA4; CA7; CBR1; CBS; CCR4; CYP1A1; CYP1B1; CYP2C8; DAPK1; ELAVL1; ELAVL3; ESR1; ESR2; ESRRB; GLO1; GPR35; GRK6; GSTO1; HSD17B1; HSD17B2; HSD17B3; KCND3; KDM4E; MAOA; MPG; MPO; NEK6; NMUR2; NOX4; NQO1; NUAK1; P4HB; PGF; PKN1; PTPRS; RPS6KA3; ST6GAL1; TNKS; TNKS2; TOP2A; TTR; VEGFA; XDH
TargetNet	41	ABCG2; ADRA2C; AHR; AKR1B1; ALOX15; ALPL; AR; BCL2A1; CA12; CA13; CA14; CA4; CA5A; CA5B; CA6; CA7; CA9; CDC25B; CES2; CTDSP1; CYP19A1; DNMT1; DUSP3; ESR1; ESR2; GPR35; HNF4A; HSD17B1; HSD17B2; HSD17B3; MAOA; MAOB; MIF; PLIN5; PTGS1; PTPN1; PTPN7; RELA; RIPK2; TERT; TLR9
SwissTargetPrediction	97	ABCB1; ABCC1; ABCG2; ACHE; ADORA3; AHR; AKR1B1; AKR1B10; AKT1; ALK; ALKBH5; ALOX12; ALOX15; ALOX5; APP; AURKB; AXL; BACE1; BCHE; CA1; CA12; CA2; CA3; CA4; CA5A; CA6; CA7; CA9; CDK2; CDK6; CISD1; CSNK2A1; CTNNB1; CYP19A1; CYP1B1; DAPK1; DPP4; EGFR; ELAVL3; ESR2; ESRRA; FBP1; FLT3; FTO; GLO1; GPR35; GSK3B; HDAC6; HSD17B1; HSD17B2; IGF1R; IP6K2; IPMK; KDR; MAOA; MAOB; MAPT; MET; MKNK2; MMP2; MMP9; MPG; MPO; NEK2; NEK6; NOX4; NUAK1; P4HB; PIK3CA; PIM1; PKM; PKN1; PLK1; PPARG; PSMB5; PTK2; PTPN1; PTPRS; RPS6KA1; RPS6KA2; RPS6KA3; SETD7; SIRT6; SLC16A1; SLC22A12; SLC2A1; SLCO2B1; SNCA; SRC; SYK; TNKS; TNKS2; TRPC5; TTR; TYR; XDH; YES1

11. Kaempferol oxoanion (PubChem CID 25202062)

Target set	n	Gene symbols
Merged unique	154	ABCB1; ABCC1; ABCG2; ACHE; ACP1; AHR; AKR1B1; AKR1B10; AKT1; ALK; ALOX12; ALOX15; ALOX5; ALPI; ALPL; AMY1A; ANTXR2; APEX1; APP; AURKB; AVPR2; AXL; BACE1; BCL2A1; BRAF; CA1; CA12; CA13; CA14; CA2; CA3; CA4; CA5A; CA5B; CA6; CA7; CA9; CAMK2B; CASP9; CBR1; CBS; CCR4; CDC25B; CDK1; CES1; CES2; CSNK2A1; CTDSP1; CTNNB1; CXCR1; CYP19A1; CYP1A1; CYP1A2; CYP1B1; CYP2C8; CYP2C9; DAPK1; DNMT1; DPP4; DRD4; DUSP3; EGFR; ELAVL1; ELAVL3; ENPP1; ESR1; ESR2; F2; FBP1; FLT3; FTO; GLO1; GPR35; GRK6; GSK3A; GSK3B; GSTO1; HDAC6; HNF4A; HSD17B1; HSD17B2; HSD17B3; IGF1R; INSR; IP6K2; IPMK; ITGB3; KCND3; KDM1A; KDM4E; KDR; MAOA; MAOB; MAPT; MBNL1; MCL1; MET; MIF; MMP13; MMP2; MMP3; MMP9; MPG; MPO; MYLK; NEK2; NEK6; NMUR2; NOX4; NUAK1; P4HB; PGF; PIK3CA; PIK3CG; PIK3R1; PIM1; PKM; PKN1; PLA2G1B; PLK1; PNLIP; PSMB5; PTGS1; PTGS2; PTK2; PTPN1; PTPN22; PTPN7; PTPRC; PTPRS; PYGL; QDPR; RAD52; RELA; RPS6KA3; SIRT6; SLC11A2; SLC22A12; SLCO2B1; SNCA; SPHK1; SRC; ST6GAL1; STS; SYK; TERT; TLR9; TOP2A; TRPC5; TTR; VEGFA; XDH; YES1
BATMAN-TCM	2	ITGB3; SLC11A2
SEA	95	ABCB1; ABCC1; ABCG2; ACP1; AKR1B1; AKR1B10; AKT1; ALK; ALOX12; ALOX15; ALOX5; ALPI; AMY1A; ANTXR2; APP; AURKB; AVPR2; AXL; BACE1; BRAF; CA1; CA12; CA14; CA2; CA3; CA4; CA5A; CA6; CA7; CA9; CAMK2B; CBR1; CBS; CCR4; CDK1; CSNK2A1; CXCR1; CYP1A1; CYP1A2; CYP1B1; CYP2C8; CYP2C9; DAPK1; DPP4; DRD4; EGFR; ELAVL1; ELAVL3; ESR1; ESR2; F2; FLT3; GLO1; GPR35; GRK6; GSK3B; GSTO1; HSD17B2; IGF1R; IL2; KCND3; KDM4E; KDR; MAOA; MAPT; MET; MMP13; MMP2; MMP3; MMP9; MPG; MPO; MYLK; NEK2; NEK6; NMUR2; NOX4; NUAK1; P4HB; PGF; PIK3R1; PIM1; PKN1; PLA2G1B; PTK2; PTPRS; PYGL; QDPR; RPS6KA3; SNCA; ST6GAL1; TERT; TTR; VEGFA; XDH
TargetNet	51	ABCB1; ABCG2; AHR; AKR1B1; ALOX15; ALOX5; ALPL; BCL2A1; CA12; CA13; CA14; CA4; CA5A; CA5B; CA6; CA7; CA9; CASP9; CDC25B; CES1; CES2; CTDSP1; CYP19A1; CYP1A2; DNMT1; DUSP3; ESR1; ESR2; GPR35; GSK3A; HNF4A; HSD17B1; HSD17B2; HSD17B3; MAOA; MAOB; MCL1; MIF; PIM1; PLA2G1B; PTGS1; PTGS2; PTPN1; PTPN22; PTPN7; PTPRC; RELA; RPS6KA3; STS; TERT; TLR9
SwissTargetPrediction	98	ABCB1; ABCC1; ABCG2; ACHE; AKR1B1; AKT1; ALK; ALOX12; ALOX15; ALOX5; APEX1; APP; AURKB; AVPR2; AXL; BACE1; BRAF; CA1; CA12; CA14; CA2; CA3; CA4; CA5A; CA6; CA7; CA9; CAMK2B; CDK1; CSNK2A1; CTNNB1; CXCR1; CYP19A1; CYP1A1; CYP1B1; CYP2C8; DAPK1; DPP4; DRD4; EGFR; ELAVL1; ELAVL3; ENPP1; ESR2; F2; FBP1; FLT3; FTO; GLO1; GPR35; GSK3B; HDAC6; HSD17B2; IGF1R; INSR; IP6K2; IPMK; KDM1A; KDM4E; KDR; MAOA; MAPT; MBNL1; MET; MMP13; MMP2; MMP3; MMP9; MPO; MYLK; NEK2; NEK6; NOX4; NUAK1; PIK3CA; PIK3CG; PIK3R1; PIM1; PKM; PKN1; PLA2G1B; PLK1; PNLIP; PSMB5; PTK2; PYGL; RAD52; SIRT6; SLC22A12; SLCO2B1; SNCA; SPHK1; SRC; SYK; TOP2A; TRPC5; XDH; YES1

12. (1S,2R,8S)-2,6,6,8-tetramethyltricyclo[5.3.1.01,5]undecan-8-ol (PubChem CID 44123431)

Target set	n	Gene symbols
Merged unique	79	AKR1B1; AKR1B10; ALOX15; ALPL; APOBEC3A; AR; CA1; CA2; CA4; CA5A; CA5B; CA6; CACNA1A; CACNA1C; CACNA1D; CACNA1F; CACNA1S; CACNB1; CACNB2; CACNB3; CACNB4; CASP9; CDC25A; CDC25B; CES1; CES2; CHRM1; CHRM2; CHRM4; CYP17A1; CYP19A1; CYP1A2; CYP8B1; DYRK1A; ENPP2; EPHX2; ESR1; ESR2; F10; G6PD; GPBAR1; GRIN2A; HSD11B1; HSD11B2; HSD17B3; HTR1E; HTR5A; IDO1; MGLL; NOS3; NPC1L1; NR1H3; NR1H4; NR1I3; NR2F2; NR3C1; NR3C2; OPRK1; OSBP; OSBP2; PGR; POLA1; PTPN1; PTPN2; RELA; RIPK2; S1PR2; SERPINA6; SHBG; SLC10A1; SLC6A3; SRD5A1; SRD5A2; TLR9; TRPA1; TRPM8; TRPV3; UGT2B7; VDR
BATMAN-TCM	13	CACNA1A; CACNA1C; CACNA1D; CACNA1F; CACNA1S; CACNB1; CACNB2; CACNB3; CACNB4; OPRK1; TRPA1; TRPM8; TRPV3
SEA	0	None retained at the stated threshold
TargetNet	29	ALOX15; ALPL; APOBEC3A; AR; CA5A; CA5B; CA6; CASP9; CDC25B; CES2; CHRM1; CHRM2; CHRM4; CYP17A1; CYP1A2; DYRK1A; HSD11B1; HSD11B2; HTR1E; HTR5A; MGLL; NOS3; NR2F2; RIPK2; S1PR2; SLC6A3; SRD5A1; SRD5A2; TLR9
SwissTargetPrediction	43	AKR1B1; AKR1B10; AR; CA1; CA2; CA4; CDC25A; CDC25B; CES1; CES2; CYP19A1; CYP8B1; ENPP2; EPHX2; ESR1; ESR2; F10; G6PD; GPBAR1; GRIN2A; HSD11B1; HSD11B2; HSD17B3; IDO1; NPC1L1; NR1H3; NR1H4; NR1I3; NR3C1; NR3C2; OSBP; OSBP2; PGR; POLA1; PTPN1; PTPN2; RELA; SERPINA6; SHBG; SLC10A1; TRPM8; UGT2B7; VDR

13. Quercetin-7-olate (PubChem CID 46906036)

Target set	n	Gene symbols
Merged unique	111	ABCB1; ABCC1; ABCG2; ACHE; ADORA3; AHR; AKR1B1; AKR1C3; ALOX12; ALOX15; ALOX5; ALPL; APP; AR; AXL; BACE1; BCHE; BCL2; BCL2A1; CA1; CA12; CA13; CA14; CA2; CA3; CA4; CA5A; CA5B; CA6; CA7; CA9; CACNA1H; CASP9; CBR1; CBS; CDC25B; CES1; CES2; CLK2; CTDSP1; CYP17A1; CYP19A1; CYP1A2; CYP1B1; DNMT1; DPP4; DUSP3; ELAVL3; ESR1; ESR2; FGFR1; FLT3; FUT4; FUT7; GPR35; GSK3A; HDAC6; HNF4A; HSD17B1; HSD17B2; HSD17B3; HTR1E; KDR; KIT; KLK1; KLK2; MAOA; MAOB; MCL1; MET; MIF; MMP12; MMP13; MMP2; MMP9; PDE4D; PGAM1; PGD; PGF; PIM1; PKM; PLA2G10; PLA2G1B; PLA2G2A; PLA2G5; PLK1; PPARG; PSMB5; PTGS1; PTGS2; PTPN1; PTPN22; PTPN7; PTPRC; RAD52; RELA; RPS6KA3; SERPINE1; SHBG; SNCA; SOST; SRC; ST3GAL3; SYNJ2; TAS2R31; TERT; TLR9; TTR; TYR; VEGFA; XDH
BATMAN-TCM	0	None retained at the stated threshold
SEA	15	CA3; CBS; CYP1B1; ELAVL3; ESR1; ESR2; FUT4; FUT7; KLK2; PGAM1; PGD; PGF; ST3GAL3; TAS2R31; VEGFA

Target set	n	Gene symbols
TargetNet	53	ABCB1; ABCG2; AHR; AKR1B1; ALOX15; ALOX5; ALPL; BCL2A1; CA12; CA13; CA14; CA4; CA5A; CA5B; CA6; CA7; CA9; CASP9; CDC25B; CES1; CES2; CTDSP1; CYP17A1; CYP19A1; CYP1A2; DNMT1; DUSP3; ESR1; ESR2; GPR35; GSK3A; HNF4A; HSD17B1; HSD17B2; HSD17B3; HTR1E; MAOA; MAOB; MCL1; MIF; MMP12; PIM1; PLA2G1B; PTGS1; PTGS2; PTPN1; PTPN22; PTPN7; PTPRC; RELA; RPS6KA3; TERT; TLR9
SwissTargetPrediction	73	ABCB1; ABCC1; ABCG2; ACHE; ADORA3; AKR1B1; AKR1C3; ALOX12; ALOX15; APP; AR; AXL; BACE1; BCHE; BCL2; CA1; CA12; CA2; CA3; CA4; CA5A; CA5B; CA6; CA7; CA9; CACNA1H; CBR1; CBS; CLK2; CYP19A1; CYP1B1; DPP4; ESR1; ESR2; FGFR1; FLT3; HDAC6; HSD17B1; KDR; KIT; KLK1; KLK2; MAOB; MET; MMP12; MMP13; MMP2; MMP9; PDE4D; PGF; PKM; PLA2G10; PLA2G2A; PLA2G5; PLK1; PPARG; PSMB5; PTGS1; PTGS2; PTPN1; RAD52; RPS6KA3; SERPINE1; SHBG; SNCA; SOST; SRC; SYNJ2; TAS2R31; TTR; TYR; VEGFA; XDH

14. (3S)-3,5,7-trihydroxy-2-(4-hydroxyphenyl)-2,3-dihydrochromen-4-one (PubChem CID 118855455)

Target set	n	Gene symbols
Merged unique	145	ABCB1; ABCC1; ABCG2; ACHE; ACP1; ADRA2C; AHR; AKR1B1; AKR1B10; AKT1; ALK; ALOX12; ALOX15; ALOX5; ALPI; AMY1A; ANTXR2; APEX1; APP; AURKB; AVPR2; AXL; BACE1; BCL2A1; BRAF; CA1; CA12; CA13; CA14; CA2; CA3; CA4; CA5A; CA5B; CA6; CA7; CA9; CAMK2B; CASP9; CBR1; CBS; CCR4; CDK1; CES2; CSNK2A1; CTNNB1; CXCR1; CYP19A1; CYP1A1; CYP1B1; CYP2C8; DAPK1; DNMT1; DPP4; DRD4; DUSP3; EGFR; ELAVL1; ELAVL3; ENPP1; ERN1; ESR1; ESR2; F2; FBP1; FLT3; FTO; GLO1; GPR35; GRK6; GSK3B; GSTO1; HCAR2; HDAC6; HNF4A; HSD17B1; HSD17B2; HSD17B3; IGF1R; IL2; INSR; IP6K2; IPMK; KCND3; KDM1A; KDM4E; KDR; MAOA; MAOB; MAPT; MBNL1; MET; MIF; MMP13; MMP2; MMP3; MMP9; MPG; MPO; MYLK; NEK2; NEK6; NMUR2; NOX4; NUAK1; P4HB; PGF; PIK3CA; PIK3CG; PIK3R1; PIM1; PKM; PKN1; PLA2G1B; PLK1; PNLIP; PSMB5; PTGS1; PTK2; PTPN1; PTPN7; PTPRS; PYGL; RAD52; RELA; RIPK2; RPS6KA3; SIRT6; SLC22A12; SLCO2B1; SNCA; SPHK1; SRC; ST6GAL1; STS; SYK; TERT; TLR9; TNKS2; TOP2A; TRPC5; TTR; VEGFA; XDH; YES1
BATMAN-TCM	0	None retained at the stated threshold
SEA	58	ABCB1; ABCC1; ABCG2; ACP1; AKR1B1; AKR1B10; ALOX12; ALOX15; ALOX5; ALPI; AMY1A; ANTXR2; CA12; CA3; CA4; CA7; CBR1; CBS; CCR4; CYP1A1; CYP1B1; CYP2C8; DAPK1; ELAVL1; ELAVL3; ERN1; ESR1; ESR2; GLO1; GPR35; GRK6; GSTO1; HSD17B1; HSD17B2; IL2; KCND3; KDM4E; MAOA; MAPT; MPG; MPO; NEK6; NMUR2; NOX4; NUAK1; P4HB; PGF; PKN1; PTPRS; RPS6KA3; SNCA; ST6GAL1; TERT; TNKS2; TOP2A; TTR; VEGFA; XDH
TargetNet	38	ABCG2; ADRA2C; AHR; AKR1B1; ALOX15; BCL2A1; CA12; CA13; CA14; CA4; CA5A; CA5B; CA6; CA7; CASP9; CES2; CYP19A1; DNMT1; DUSP3; ESR1; ESR2; GPR35; HCAR2; HNF4A; HSD17B1; HSD17B2; HSD17B3; MAOA; MAOB; MIF; PTGS1; PTPN1; PTPN7; RELA; RIPK2; STS; TERT; TLR9
SwissTargetPrediction	98	ABCB1; ABCC1; ABCG2; ACHE; AKR1B1; AKT1; ALK; ALOX12; ALOX15; ALOX5; APEX1; APP; AURKB; AVPR2; AXL; BACE1; BRAF; CA1; CA12; CA14; CA2; CA3; CA4; CA5A; CA6; CA7; CA9; CAMK2B; CDK1; CSNK2A1; CTNNB1; CXCR1; CYP19A1; CYP1A1; CYP1B1; CYP2C8; DAPK1; DPP4; DRD4; EGFR; ELAVL1; ELAVL3; ENPP1; ESR2; F2; FBP1; FLT3; FTO; GLO1; GPR35; GSK3B; HDAC6; HSD17B2; IGF1R; INSR; IP6K2; IPMK; KDM1A; KDM4E; KDR; MAOA; MAPT; MBNL1; MET; MMP13; MMP2; MMP3; MMP9; MPO; MYLK; NEK2; NEK6; NOX4; NUAK1; PIK3CA; PIK3CG; PIK3R1; PIM1; PKM; PKN1; PLA2G1B; PLK1; PNLIP; PSMB5; PTK2; PYGL; RAD52; SIRT6; SLC22A12; SLCO2B1; SNCA; SPHK1; SRC; SYK; TOP2A; TRPC5; XDH; YES1

15. (2R)-2-(4-hydroxyphenyl)-3,4-dihydro-2H-chromene-3,5,7-triol (PubChem CID 9943440)

Target set	n	Gene symbols
Merged unique	115	ABCB1; ABCC1; ABCG2; ACHE; ADAMTS4; ADAMTS5; ADORA3; ADRA2C; AHR; AKT1; ALOX15; ALOX5; APP; AR; AXL; BACE1; BCL2; CA1; CA12; CA14; CA2; CA3; CA4; CA5A; CA5B; CA6; CA7; CA9; CACNA1B; CACNA1H; CBR1; CBS; CDK2; CES2; CLK1; CLK2; CLK3; CLK4; CSNK1E; CYP19A1; CYP1B1; DNMT1; DPP4; DRD1; DRD5; DYRK1A; DYRK1B; DYRK2; DYRK3; DYRK4; EGFR; ELAVL3; ERBB2; ESR1; ESR2; FGFR1; FLT3; FUT4; FUT7; FYN; GALR3; GSK3B; HDAC6; HIF1A; HSD17B1; HSD17B2; HSD17B3; KCNH2; KDR; KIT; KLK1; KLK2; MAOA; MAOB; MAPK14; MAPKAPK5; MAPT; MET; MIF; MMP1; MMP13; MMP14; MMP2; MMP9; MTOR; OPRD1; PGAM1; PGD; PGF; PIK3CA; PKM; PLK1; PRKAA2; PSMB5; PTGES; PTGS1; PTPN1; RAD52; RELA; RPS6KA3; SERPINE1; SHBG; SIRT6; SNCA; SOST; SRC; ST3GAL3; STAT1; SYNJ2; TAS2R31; TERT; TTR; TYR; VEGFA; XDH
BATMAN-TCM	0	None retained at the stated threshold
SEA	16	CA3; CBS; CYP1B1; ELAVL3; ESR1; ESR2; FUT4; FUT7; KLK2; PGAM1; PGD; PGF; PTGS1; ST3GAL3; TAS2R31; VEGFA
TargetNet	27	ADRA2C; AHR; ALOX15; AR; CA12; CA14; CA4; CA5A; CA5B; CA6; CA7; CACNA1B; CYP19A1; DRD1; DRD5; ESR1; ESR2; GALR3; HSD17B1; HSD17B2; HSD17B3; MAOA; MAOB; MIF; OPRD1; PTGS1; RELA
SwissTargetPrediction	96	ABCB1; ABCC1; ABCG2; ACHE; ADAMTS4; ADAMTS5; ADORA3; AKT1; ALOX5; APP; AXL; BACE1; BCL2; CA1; CA12; CA2; CA3; CA4; CA5A; CA5B; CA6; CA7; CA9; CACNA1H; CBR1; CBS; CDK2; CES2; CLK1; CLK2; CLK3; CLK4; CSNK1E; CYP19A1; CYP1B1; DNMT1; DPP4; DYRK1A; DYRK1B; DYRK2; DYRK3; DYRK4; EGFR; ELAVL3; ERBB2; ESR1; ESR2; FGFR1; FLT3; FYN; GSK3B; HDAC6; HIF1A; HSD17B1; KCNH2; KDR; KIT; KLK1; KLK2; MAOB; MAPK14; MAPKAPK5; MAPT; MET; MMP1; MMP13; MMP14; MMP2; MMP9; MTOR; PGAM1; PGF; PIK3CA; PKM; PLK1; PRKAA2; PSMB5; PTGES; PTGS1; PTPN1; RAD52; RPS6KA3; SERPINE1; SHBG; SIRT6; SNCA; SOST; SRC; STAT1; SYNJ2; TAS2R31; TERT; TTR; TYR; VEGFA; XDH

16. Gallic Acid (PubChem CID 370)

Target set	n	Gene symbols
Merged unique	163	ABCB1; ABCG2; ACE2; ACHE; AHR; AKR1B1; AKR1C1; AKR1C2; AKR1C3; ALK; ALOX15; ALOX5; ALPL; AMY1A; ANTXR2; APOBEC3G; APP; ATG4B; BACE1; BCL2A1; BCL2L1; CA1; CA12; CA13; CA14; CA2; CA3; CA4; CA5A; CA5B; CA6; CA7; CA9; CASP3; CASP9; CAT; CDC25A; CDC25B; CES1; CES2; CSNK2A1; CTDSP1; CTRC; DDO; DGAT1; DGKA; DHODH; DNMT1; DPP4; DRD5; DUSP3; ERN1; ESR1; ESR2; F2; FASLG; FOLH1; FTO; FUT7; FYN; GALNT2; GLRA3; GLRB; GPR35; GRIN2A; GRIN2B; GSK3A; HCAR2; HIF1AN; HMGB1; HSD17B1; HSD17B2; HSD17B3; HTR1E; IGF1R; IGFBP1; IGFBP2; IGFBP3; IGFBP4; IGFBP5; IGFBP6; IL33; KDM2A; KDM3A; KDM4D; KDM4E; KDM5C; KIF11; LDHA; LDHB; LPO; MAOA; MAOB; MARK4; MCL1; MGLL; MGST1; MIF; MMP2; MMP9; MYD88; NAPRT; NFE2L2; NFKB1; NOS2; NR1I2; NR4A1; P2RX7; P4HA1; P4HTM; PIK3CG; PLAU; PLIN1; PLIN5; POLA1; POLB; PPP2CA; PPP2CB; PRKCA; PRKCB; PRKCD; PRKCE; PTGER2; PTGR1; PTGS1; PTGS2; PTPN1; PTPN11; PTPN22; PTPN7; PTPRC; RAB13; RAC1; RARA; RARB; RARG; RELA; RIPK2; RXRA; RXRB; RXRG; SEC14L2; SEC14L3; SEC14L4; SELE; SELL; SELP; SERPINE1; SIRT1; SNCA; SRD5A2; STS; SULT1A1; THRA; THRB; TLR9; TOP1; TP53; TPMT; TTR; TYMS; TYR; VTN
BATMAN-TCM	42	ABCB1; ACE2; ACHE; ALOX5; BACE1; CASP3; CASP9; CAT; CDC25A; DGAT1; DGKA; DPP4; F2; FASLG; IL33; LPO; MAOA; MARK4; MGST1; MMP2; MMP9; MYD88; NFE2L2; NFKB1; NOS2; NR1I2; P2RX7; PIK3CG; PPP2CA; PPP2CB; PRKCA; PRKCB; PRKCD; PTGR1; RAB13; SEC14L2; SEC14L3; SEC14L4; SNCA; SULT1A1; TP53; VTN
SEA	55	AKR1C1; AKR1C2; AKR1C3; AMY1A; ANTXR2; CA1; CA12; CA14; CA2; CA3; CA4; CA5A; CA5B; CA6; CA7; CA9; CES1; CES2; CTRC; DDO; DPP4; ERN1; FTO; FUT7; GLRA3; GLRB; GPR35; HIF1AN; IGFBP1; IGFBP2; IGFBP3; IGFBP4; IGFBP5; IGFBP6; KDM2A; KDM3A; KDM4D; KDM4E; KDM5C; NR4A1; P4HA1; P4HTM; POLA1; POLB; RARB; RARG; SELE; SELL; SELP; SERPINE1; SRD5A2; TOP1; TPMT; TTR; TYR
TargetNet	72	ABCG2; AHR; AKR1B1; AKR1C3; ALOX15; ALPL; APOBEC3G; APP; BCL2A1; CA1; CA12; CA13; CA14; CA2; CA4; CA5A; CA5B; CA6; CA7; CA9; CASP9; CDC25B; CES1; CES2; CSNK2A1; CTDSP1; DHODH; DNMT1; DRD5; DUSP3; ESR1; ESR2; FOLH1; GPR35; GSK3A; HCAR2; HSD17B1; HSD17B2; HSD17B3; HTR1E; KIF11; MAOA; MAOB; MCL1; MGLL; MIF; PLAU; PLIN1; PLIN5; PRKCE; PTGER2; PTGS1; PTGS2; PTPN1; PTPN22; PTPN7; PTPRC; RAC1; RARA; RARB; RARG; RELA; RIPK2; RXRA; RXRB; RXRG; SERPINE1; SIRT1; STS; THRA; THRB; TLR9
SwissTargetPrediction	35	ALK; ATG4B; BCL2L1; CA1; CA12; CA14; CA2; CA3; CA4; CA5A; CA5B; CA6; CA7; CA9; DPP4; ESR2; FUT7; FYN; GALNT2; GPR35; GRIN2A; GRIN2B; HMGB1; HSD17B2; IGF1R; LDHA; LDHB; MAOB; NAPRT; PTPN11; SERPINE1; SNCA; TTR; TYMS; TYR

17. Cianidanol (PubChem CID 9064)

Target set	n	Gene symbols
Merged unique	122	ABCB1; ABCC1; ABCG2; ADAMTS4; ADAMTS5; ADRA2B; ADRA2C; AHR; AKT1; ALOX12; ALOX15; ALPL; APP; AR; AXL; BACE1; BCL2; BCL2A1; CA1; CA12; CA14; CA2; CA3; CA4; CA5A; CA5B; CA6; CA7; CA9; CACNA1B; CBS; CDC25B; CDK2; CLK1; CLK2; CLK3; CLK4; CSNK1E; CYP19A1; CYP1B1; DHFR; DNMT1; DPP4; DRD1; DRD5; DYRK1A; DYRK1B; DYRK2; DYRK3; DYRK4; EGFR; ELAVL3;

Target set	n	Gene symbols
		ERBB2; ESR1; ESR2; FASN; FGB; FGFR1; FLT1; FLT3; FUT4; FUT7; FYN; GSK3B; HDAC6; HSD17B1; HSD17B2; HSD17B3; HSP90AA1; KCNH2; KDR; KIT; KLF7; KLK1; KLK2; MAOA; MAOB; MAPK14; MAPKAPK5; MAPT; MET; MIF; MMP1; MMP12; MMP13; MMP14; MMP2; MMP9; MTOR; NADK; OPRM1; PGAM1; PGD; PGF; PIK3CA; PKM; PLK1; PPARG; PRKAA2; PSMB5; PTGES; PTGS1; PTPN1; QDPR; RAD52; RELA; RPS6KA3; RPSA; SIRT6; SNCA; SOST; SRC; ST3GAL3; STAT1; SYNJ2; TAS2R31; TERT; TTR; TYR; VEGFA; XDH; YWHAG
BATMAN-TCM	4	DHFR; FASN; KLF7; PPARG
SEA	27	ALPL; CA1; CA12; CA2; CA3; CA4; CA5A; CA5B; CA6; CA7; CA9; CBS; CYP1B1; ELAVL3; ESR1; ESR2; FUT4; FUT7; KLK2; PGAM1; PGD; PGF; QDPR; ST3GAL3; TAS2R31; VEGFA; YWHAG
TargetNet	32	ADRA2B; ADRA2C; AHR; ALOX15; ALPL; AR; BCL2A1; CA12; CA14; CA4; CA5A; CA5B; CA6; CA7; CACNA1B; CDC25B; CYP19A1; DRD1; DRD5; ESR1; ESR2; HSD17B1; HSD17B2; HSD17B3; HSP90AA1; MAOA; MAOB; MIF; OPRM1; PTGS1; RELA; TERT
SwissTargetPrediction	96	ABCB1; ABCC1; ABCG2; ADAMTS4; ADAMTS5; AKT1; ALOX12; ALOX15; APP; AXL; BACE1; BCL2; CA1; CA12; CA2; CA3; CA4; CA5A; CA5B; CA6; CA7; CA9; CDK2; CLK1; CLK2; CLK3; CLK4; CSNK1E; CYP19A1; CYP1B1; DNMT1; DPP4; DYRK1A; DYRK1B; DYRK2; DYRK3; DYRK4; EGFR; ELAVL3; ERBB2; ESR1; ESR2; FGB; FGFR1; FLT1; FLT3; FUT4; FUT7; FYN; GSK3B; HDAC6; HSD17B1; KCNH2; KDR; KIT; KLK1; KLK2; MAOB; MAPK14; MAPKAPK5; MAPT; MET; MMP1; MMP12; MMP13; MMP14; MMP2; MMP9; MTOR; NADK; PGAM1; PGD; PGF; PIK3CA; PKM; PLK1; PRKAA2; PSMB5; PTGES; PTGS1; PTPN1; RAD52; RPS6KA3; RPSA; SIRT6; SNCA; SOST; SRC; ST3GAL3; STAT1; SYNJ2; TERT; TTR; TYR; VEGFA; XDH

Note. A target appearing in more than one prediction source is listed under each contributing database but counted only once in the merged unique-target count for that compound. Prediction thresholds were TargetNet probability ≥0.90, SEA probability ≥0.30, BATMAN-TCM score ≥80, and SwissTargetPrediction probability >0.10.

Table S1E. Global nonredundant predicted-target set after merging all 17 retained compounds (n = 966).

Target 1	Target 2	Target 3	Target 4	Target 5	Target 6	Target 7	Target 8
A1BG	CA12	CYP19A1	GABBR1	IGFBP1	MMP12	PLA2G2C	SEMG1
A2M	CA13	CYP1A1	GABBR2	IGFBP2	MMP13	PLA2G2D	SEPP1
ABCA1	CA14	CYP1A2	GABRA1	IGFBP3	MMP14	PLA2G2E	SERPINA1
ABCB1	CA2	CYP1B1	GABRA2	IGFBP4	MMP2	PLA2G4B	SERPINA3
ABCB11	CA3	CYP2A13	GABRA3	IGFBP5	MMP3	PLA2G5	SERPINA4
ABCC1	CA4	CYP2A6	GABRA4	IGFBP6	MMP9	PLAA	SERPINA6
ABCC2	CA5A	CYP2A7	GABRA5	IL10	MPEG1	PLAT	SERPIND1
ABCG2	CA5B	CYP2B6	GABRA6	IL17A	MPG	PLAU	SERPINE1
ABHD15	CA6	CYP2C18	GABRB1	IL1A	MPO	PLD1	SETD7
ACADSB	CA7	CYP2C19	GABRB2	IL1B	MRC1	PLD2	SHBG
ACE	CA9	CYP2C8	GABRB3	IL2	MT1A	PLEC	SIRT1
ACE2	CACNA1A	CYP2C9	GABRD	IL3	MT1B	PLEK	SIRT2
ACER2	CACNA1B	CYP2E1	GABRE	IL33	MT1E	PLG	SIRT6
ACHE	CACNA1C	CYP2F1	GABRG1	IL6	MT1F	PLIN1	SIVA1
ACLY	CACNA1D	CYP2S1	GABRG2	INS	MT1G	PLIN5	SLC10A1
ACP1	CACNA1F	CYP3A4	GABRG3	INSR	MT1H	PLK1	SLC11A2
ACVRL1	CACNA1H	CYP4A11	GABRP	IP6K2	MT1X	PMP2	SLC16A1
ADAMTS4	CACNA1S	CYP4F2	GABRQ	IPMK	MT2A	PNLIP	SLC16A3
ADAMTS5	CACNA2D1	CYP8B1	GABRR1	IRF1	MT3	POLA1	SLC22A1
ADH1A	CACNA2D2	DAGLA	GABRR3	ITGA2B	MTA3	POLB	SLC22A12
ADH1B	CACNB1	DAND5	GALNT2	ITGB3	MTOR	POLH	SLC22A6
ADH1C	CACNB2	DAPK1	GALR3	ITIH1	MUC5AC	POLK	SLC22A8
ADH5	CACNB3	DCD	GAPDHS	ITIH2	MYC	POLL	SLC23A1
ADH7	CACNB4	DDIT3	GBA	ITIH3	MYD88	POLM	SLC25A20
ADIPOQ	CALM1	DDO	GCK	ITIH4	MYLK	PON1	SLC2A1
ADORA2A	CAMK2B	DGAT1	GGPS1	ITPR3	MYLK3	POR	SLC2A4
ADORA3	CASP3	DGKA	GK	JAK1	NAAA	PPARA	SLC37A4
ADRA2B	CASP8	DHFR	GLO1	JAK2	NADK	PPARD	SLC6A11
ADRA2C	CASP9	DHODH	GLRA1	JCHAIN	NAPRT	PPARG	SLC6A3
AGT	CAT	DIO1	GLRA3	JUN	NAT10	PPP1R14A	SLCO1B1
AHR	CAV1	DNAJC5	GLRB	JUP	NCF1	PPP2CA	SLCO2A1
AHSG	CBR1	DNM1	GLTP	KCNA3	NDUFV2	PPP2CB	SLCO2B1
AHSP	CBS	DNMT1	GM2A	KCND3	NEIL1	PPT1	SLPI
AKR1B1	CCL2	DPP4	GNAI1	KCNH2	NEIL2	PRDX1	SMAD3
AKR1B10	CCL27	DRD1	GNAI3	KDM1A	NEK2	PRKAA1	SNCA
AKR1C1	CCNB1	DRD4	GNAO1	KDM2A	NEK6	PRKAA2	SOAT1

Target 1	Target 2	Target 3	Target 4	Target 5	Target 6	Target 7	Target 8
AKR1C2	CCND1	DRD5	GPBAR1	KDM3A	NFATC1	PRKCA	SOAT2
AKR1C3	CCR4	DSP	GPER1	KDM4A	NFE2L2	PRKCB	SOD1
AKR1D1	CCS	DUSP3	GPHN	KDM4C	NFKB1	PRKCD	SOST
AKT1	CD274	DYRK1A	GPR174	KDM4D	NFKBIA	PRKCE	SPHK1
ALDH2	CD40LG	DYRK1B	GPR18	KDM4E	NGF	PSMB1	SPP1
ALDH5A1	CD86	DYRK2	GPR34	KDM5A	NKX3-1	PSMB5	SPTLC2
ALDOA	CDC25A	DYRK3	GPR35	KDM5C	NLRP3	PSPH	SRC
ALK	CDC25B	DYRK4	GPR84	KDM7A	NME1	PTEN	SRD5A1
ALKBH2	CDC25C	E2F1	GRIN1	KDR	NMUR2	PTGER2	SRD5A2
ALKBH3	CDC42	E2F2	GRIN2A	KHK	NOD1	PTGER3	ST3GAL3
ALKBH5	CDC45	EDN1	GRIN2B	KIF11	NOS2	PTGER4	ST6GAL1
ALOX12	CDH1	EED	GRIN2C	KIT	NOS3	PTGES	STAT1
ALOX15	CDK1	EEF1A1	GRIN2D	KLF7	NOX4	PTGFR	STAT3
ALOX5	CDK2	EEF1A2	GRIN3A	KLK1	NPC1L1	PTGIR	STS
ALPI	CDK6	EFTUD1	GRIN3B	KLK2	NPEPPS	PTGR1	SUCNR1
ALPL	CDKN1A	EGF	GRK6	KLK7	NQO1	PTGS1	SULT1A1
ALPP	CDKN2A	EGFR	GRM5	KLKB1	NR1H3	PTGS2	SULT1E1
AMY1A	CEBPB	EGLN1	GSK3A	KNG1	NR1H4	PTK2	SYK
ANPEP	CELA1	EGR1	GSK3B	KRT1	NR1I2	PTK2B	SYNJ2
ANTXR2	CERT1	EIF2S1	GSN	KRT10	NR1I3	PTP4A3	TAS2R31
APCS	CES1	ELANE	GSR	KRT12	NR2E3	PTPN1	TBK1
APEX1	CES2	ELAVL1	GSTA1	KRT14	NR2F2	PTPN11	TBXA2R
APLNR	CFB	ELAVL3	GSTA2	KRT16	NR3C1	PTPN2	TBXAS1
APLP1	CFH	ELK1	GSTK1	KRT2	NR3C2	PTPN22	TERT
APLP2	CFI	ENO1	GSTM1	KRT5	NR4A1	PTPN7	TF
APOA1	CHEK2	ENPEP	GSTM2	KRT6A	NRF1	PTPRC	TFRC
APOA2	CHRFAM7A	ENPP1	GSTM3	KRT6B	NSFL1C	PTPRS	TGFB1
APOA4	CHRM1	ENPP2	GSTM4	KRT6C	NT5E	PTX3	TGFB2
APOBEC3A	CHRM2	EPHX1	GSTM5	KRT9	NUAK1	PYGL	THBS1
APOBEC3G	CHRM4	EPHX2	GSTO1	LALBA	OCLN	QDPR	THRA
APOBR	CHRM5	ERAP1	GSTP1	LAP3	ODC1	RAB13	THRB
APOE	CHRNA7	ERBB2	GSTT1	LCK	OGDH	RAC1	TJP1
APOL1	CHUK	ERBB3	GSTT2	LDHA	OPN1LW	RAD52	TLR2
APOM	CISD1	ERN1	GSTT2B	LDHB	OPN1MW	RAF1	TLR4
APP	CLDN2	ERN2	GUCA2A	LDLR	OPN1MW2	RARA	TLR9
AQP1	CLDN4	ESR1	HAO1	LEF1	OPN1MW3	RARB	TNF
AQP5	CLDN5	ESR2	HBA1	LGALS1	OPN1SW	RARG	TNFRSF10B
AR	CLEC4E	ESRRA	HBA2	LIMK1	OPRD1	RASSF1	TNFSF10
ASPA	CLK1	ESRRB	HBB	LIMK2	OPRK1	RB1	TNFSF11
ASRGL1	CLK2	ESRRG	HBD	LPAR1	OPRM1	RELA	TNKS
ATF4	CLK3	F10	HCAR2	LPAR2	ORM1	RHO	TNKS2
ATG4B	CLK4	F12	HDAC1	LPAR3	ORM2	RIPK2	TOP1
ATHS	CLU	F13B	HDAC10	LPAR4	OSBP	RORA	TOP2A
ATP1A1	CNR2	F2	HDAC11	LPAR6	OSBP2	RORC	TP53
ATRNL1	COL1A1	F3	HDAC2	LPCAT1	OXER1	ROS1	TP73
AURKB	COMT	FAAH	HDAC3	LPCAT2	P2RX4	RPS6KA1	TPI1
AVPR2	COX1	FABP1	HDAC4	LPO	P2RX7	RPS6KA2	TPMT
AXIN1	COX2	FABP12	HDAC5	LTA4H	P2RY10	RPS6KA3	TRAPPC3
AXIN2	COX3	FABP2	HDAC6	LTB4R	P4HA1	RPSA	TRAPPC3L
AXL	COX4I1	FABP3	HDAC7	LTF	P4HB	RUNX2	TREM2

Target 1	Target 2	Target 3	Target 4	Target 5	Target 6	Target 7	Target 8
BACE1	COX5A	FABP4	HDAC8	LY96	P4HTM	RXRA	TRPA1
BAD	COX5B	FABP5	HDAC9	LYN	PAEP	RXRB	TRPC5
BAX	COX6A2	FABP6	HGF	MAOA	PAM	RXRG	TRPM8
BBOX1	COX6B1	FABP7	HIF1A	MAOB	PAOX	S100A2	TRPV1
BCHE	COX6C	FABP9	HIF1AN	MAP2K3	PARK7	S100A7	TRPV3
BCL2	COX7A1	FASLG	HK2	MAP2K6	PARP1	S100A7A	TTR
BCL2A1	COX7B	FASN	HMGB1	MAP3K5	PCYT1A	S100A8	TUFM
BCL2L1	COX7C	FBP1	HMGCR	MAP3K7	PCYT1B	S100A9	TWIST1
BDKRB1	COX8A	FCN3	HMOX1	MAPK1	PDCD6	S1PR1	TXN
BDNF	CP	FDPS	HNF4A	MAPK10	PDE4D	S1PR2	TXNIP
BGLAP	CPN1	FECH	HNF4G	MAPK14	PDE7A	S1PR3	TYMS
BHMT	CPN2	FEN1	HNRNPA1	MAPK3	PDGFRA	S1PR4	TYR
BIRC2	CPT1A	FFAR1	HPGDS	MAPK8	PDIA3	S1PR5	UGT1A1
BIRC3	CPT2	FFAR4	HPR	MAPK8IP1	PDK3	SARS	UGT1A3
BMP2	CREB1	FGA	HRNR	MAPKAPK5	PDK4	SCD	UGT1A9
BMPR2	CRH	FGB	HSD11B1	MAPT	PDLIM3	SCN10A	UGT2B7
BRAF	CRP	FGFR1	HSD11B2	MARK4	PGAM1	SCN11A	UQCRC1
BRCA1	CRTC1	FLT1	HSD17B1	MBNL1	PGD	SCN1A	USP10
BRCC3	CRTC2	FLT3	HSD17B2	MCL1	PGF	SCN1B	UTRN
BTK	CSF2	FLT4	HSD17B3	MCU	PGLYRP2	SCN2A	VCAM1
C1QB	CSF3	FN1	HSF1	MDH1	PGP	SCN2B	VDR
C1QC	CSNK1E	FOLH1	HSP90AA1	MDM2	PGR	SCN3A	VEGFA
C1R	CSNK2A1	FOS	HSP90B2P	MECP2	PHF8	SCN3B	VIM
C1S	CSNK2A2	FOSL1	HSPA4	MET	PHOSPHO1	SCN4A	VIM2P
C3	CSNK2B	FOXD3	HSPA5	MGAM	PIK3CA	SCN4B	VLDLR
C4A	CTDSP1	FOXP3	HSPB1	MGLL	PIK3CB	SCN5A	VTN
C4B	CTNNB1	FTH1	HTR1E	MGMT	PIK3CG	SCN7A	XBP1
C4BPA	CTRC	FTO	HTR5A	MGST1	PIK3R1	SCN8A	XDH
C4BPB	CTSD	FUT4	HVCN1	MIF	PIM1	SCN9A	YES1
C4B_2	CTSG	FUT7	ICAM1	MIR145	PKLR	SEC14L2	YWHAG
C5	CXCL10	FXN	IDE	MIR21	PKM	SEC14L3	YWHAZ
C8A	CXCL2	FXR1	IDO1	MIR371A	PKN1	SEC14L4	ZDHHC2
C8B	CXCL8	FYN	IFNG	MKNK2	PLA2G10	SELE	ZDHHC20
C8G	CXCR1	G6PD	IGF1R	MLXIPL	PLA2G1B	SELL	
CA1	CYP17A1	GAA	IGFALS	MMP1	PLA2G2A	SELP	

Global source coverage in the archived merged workflow: BATMAN-TCM = 607 nonredundant targets, SwissTargetPrediction = 296; SEA = 278; TargetNet = 149. After merging across all databases and compounds, 966 unique target symbols remained.

Table S1F. Molecular docking receptor structures, grid definitions, and AutoDock Vina parameters.

Target	Structure source	Structure/UniProt ID	Center X	Center Y	Center Z	Size X	Size Y	Size Z	Grid spacing (Å)	Vina version	Exhaustiveness	CPU	Seed	Output poses
CASP3	AlphaFold DB	Q8MJU1	-10.527	8.038	-4.033	80.25	56.25	81.75	0.375	1.2.7	50	4	1000	9
IL1B	AlphaFold DB	P41687	8.098	-6.022	-29.912	74.25	85.5	101.25	0.375	1.2.7	50	4	1000	9
IL6	AlphaFold DB	P41683	-18.934	10.705	-23.222	75.0	67.5	97.5	0.375	1.2.7	50	4	1000	9
STAT3	Predicted structure supplied in docking archive	fold_a0abi7zck2_model_0	-3.841	-1.377	-7.126	96.0	123.75	122.25	0.375	1.2.7	50	4	1000	9
TLR4	AlphaFold DB	P58727	22.29	-1.06	-39.739	147.75	87.75	172.5	0.375	1.2.7	50	4	1000	9
TNF	AlphaFold DB	A0A0U5EMB8	14.908	13.953	-4.48	142.5	84.75	126.0	0.375	1.2.7	50	4	1000	9

Note. The configuration files show a grid spacing of 0.375 Å and nine ranked output modes per run. For the reported binding-energy matrix, the best-ranked pose (mode 1) was used. The supplied archive supports AlphaFold DB structures for CASP3, IL1B, IL6, TLR4, and TNF; the STAT3 file is retained here under its archived predicted-structure identifier rather than being relabelled as an experimental PDB structure.

Table S1G. Complete molecular docking results for all 60 compound–target combinations (10 representative compounds × 6 core targets).

No.	Target	PubChem CID	Compound	Best binding energy (kcal/mol)	Interpretation
1	CASP3	3220	Emodin	-7.420	Favorable (≤ −5.0)
2	CASP3	439533	Taxifolin	-7.399	Favorable (≤ −5.0)
3	CASP3	122850	Aromadendrin	-7.304	Favorable (≤ −5.0)
4	CASP3	5280343	Quercetin	-7.129	Favorable (≤ −5.0)
5	CASP3	9064	Cianidanol [(+)-catechin]	-7.104	Favorable (≤ −5.0)
6	CASP3	25202062	Kaempferol oxoanion	-7.096	Favorable (≤ −5.0)
7	CASP3	5280863	Kaempferol	-7.045	Favorable (≤ −5.0)
8	CASP3	65575	Cedrol	-6.524	Favorable (≤ −5.0)
9	CASP3	370	Gallic acid	-5.715	Favorable (≤ −5.0)
10	CASP3	985	Palmitic acid	-4.673	Weak (> −5.0)
11	IL1B	5280343	Quercetin	-7.613	Strong (≤ −7.5)
12	IL1B	439533	Taxifolin	-7.534	Strong (≤ −7.5)
13	IL1B	25202062	Kaempferol oxoanion	-7.251	Favorable (≤ −5.0)
14	IL1B	3220	Emodin	-7.116	Favorable (≤ −5.0)
15	IL1B	5280863	Kaempferol	-6.953	Favorable (≤ −5.0)
16	IL1B	122850	Aromadendrin	-6.924	Favorable (≤ −5.0)
17	IL1B	9064	Cianidanol [(+)-catechin]	-6.535	Favorable (≤ −5.0)
18	IL1B	65575	Cedrol	-5.894	Favorable (≤ −5.0)
19	IL1B	370	Gallic acid	-5.493	Favorable (≤ −5.0)
20	IL1B	985	Palmitic acid	-3.751	Weak (> −5.0)
21	IL6	9064	Cianidanol [(+)-catechin]	-8.658	Strong (≤ −7.5)
22	IL6	439533	Taxifolin	-8.492	Strong (≤ −7.5)
23	IL6	122850	Aromadendrin	-8.429	Strong (≤ −7.5)
24	IL6	5280343	Quercetin	-8.344	Strong (≤ −7.5)
25	IL6	5280863	Kaempferol	-8.235	Strong (≤ −7.5)
26	IL6	25202062	Kaempferol oxoanion	-8.140	Strong (≤ −7.5)
27	IL6	3220	Emodin	-7.833	Strong (≤ −7.5)
28	IL6	65575	Cedrol	-7.007	Favorable (≤ −5.0)
29	IL6	370	Gallic acid	-5.858	Favorable (≤ −5.0)
30	IL6	985	Palmitic acid	-3.959	Weak (> −5.0)
31	STAT3	9064	Cianidanol [(+)-catechin]	-7.905	Strong (≤ −7.5)
32	STAT3	5280343	Quercetin	-7.652	Strong (≤ −7.5)
33	STAT3	439533	Taxifolin	-7.568	Strong (≤ −7.5)
34	STAT3	25202062	Kaempferol oxoanion	-7.478	Favorable (≤ −5.0)
35	STAT3	5280863	Kaempferol	-7.327	Favorable (≤ −5.0)
36	STAT3	122850	Aromadendrin	-7.306	Favorable (≤ −5.0)
37	STAT3	3220	Emodin	-6.935	Favorable (≤ −5.0)
38	STAT3	65575	Cedrol	-6.357	Favorable (≤ −5.0)
39	STAT3	370	Gallic acid	-5.921	Favorable (≤ −5.0)
40	STAT3	985	Palmitic acid	-4.173	Weak (> −5.0)
41	TLR4	5280343	Quercetin	-7.829	Strong (≤ −7.5)
42	TLR4	439533	Taxifolin	-7.327	Favorable (≤ −5.0)
43	TLR4	25202062	Kaempferol oxoanion	-7.079	Favorable (≤ −5.0)
44	TLR4	5280863	Kaempferol	-6.891	Favorable (≤ −5.0)
45	TLR4	3220	Emodin	-6.821	Favorable (≤ −5.0)
46	TLR4	9064	Cianidanol [(+)-catechin]	-6.682	Favorable (≤ −5.0)
47	TLR4	122850	Aromadendrin	-6.574	Favorable (≤ −5.0)
48	TLR4	65575	Cedrol	-6.367	Favorable (≤ −5.0)
49	TLR4	370	Gallic acid	-6.153	Favorable (≤ −5.0)

No.	Target	PubChem CID	Compound	Best binding energy (kcal/mol)	Interpretation
50	TLR4	985	Palmitic acid	-4.174	Weak (> −5.0)
51	TNF	25202062	Kaempferol oxoanion	-7.243	Favorable (≤ −5.0)
52	TNF	122850	Aromadendrin	-6.872	Favorable (≤ −5.0)
53	TNF	5280863	Kaempferol	-6.866	Favorable (≤ −5.0)
54	TNF	439533	Taxifolin	-6.702	Favorable (≤ −5.0)
55	TNF	3220	Emodin	-6.609	Favorable (≤ −5.0)
56	TNF	9064	Cianidanol [(+)-catechin]	-6.590	Favorable (≤ −5.0)
57	TNF	5280343	Quercetin	-6.567	Favorable (≤ −5.0)
58	TNF	370	Gallic acid	-5.594	Favorable (≤ −5.0)
59	TNF	65575	Cedrol	-5.238	Favorable (≤ −5.0)
60	TNF	985	Palmitic acid	-3.879	Weak (> −5.0)

Note. Among the 60 docking combinations, 54/60 had binding energies ≤ −5.0 kcal/mol and 13/60 had binding energies ≤ −7.5 kcal/mol. The lowest recorded energy was IL6–Cianidanol [(+)-catechin] at -8.658 kcal/mol. Docking energies are computational scores and should not be interpreted as experimentally measured binding constants or evidence of target engagement in cats.

Data-transparency statement. Supplementary Data S1 contains the complete archived compound-side screening, provenance audit, database-specific target-prediction lists, global nonredundant target set, docking setup, and all 60 docking scores. Supplementary Data S2 contains the complete disease-gene audit, archived feline mapping evidence for the 102 shared targets, and the complete GO and KEGG enrichment outputs.
